# Supplementary material for: A multi-objective genetic algorithm to find active modules in multiplex biological networks
Source: PLoS Comput Biol. 2021 Aug 30;17(8):e1009263. doi: 10.1371/journal.pcbi.1009263 (PMC8452006; doi:10.1371/journal.pcbi.1009263)
Supplement: S1 File — 1. Supplementary Figs: Fig S1: Sizes of the subnetworks identified by PinnacleZ in the experiment using the network PPI_1 and the simulated data with normal distribution. Fig S2: Sizes of the subnetworks identified by PinnacleZ in the experiment using the network PPI_2 and the sampled data from RNA-Seq TCGA breast cancer dataset. Fig S3: Sizes of the subnetworks identified by COSINE in the experiment using the network PPI_1 and the simulated data with normal distribution. Fig S4: Sizes of the subnetworks identified by COSINE in the experiment using the network PPI_2 and the sampled data from RNA-Seq TCGA breast cancer dataset. Fig S5: Sizes of the subnetworks identified by jActiveModules in the experiment using the network PPI_1 and the simulated data with normal distribution. Fig S6: Sizes of the subnetworks identified by jActiveModules in the experiment using the network PPI_2 and the sampled data from RNA-Seq TCGA breast cancer dataset. Fig S7: Sizes of the subnetworks identified by all the methods in the experiment using the network PPI_1 and the simulated data with normal distribution. Fig S8: Sizes of the subnetworks identified by all the methods in the experiment using the network PPI_2 and the sampled data from RNA-Seq TCGA breast cancer dataset. Fig S9: Yao’s dataset, biopsies: Active modules 1–18. Fig S10: Yao’s dataset, myoblasts: Active modules 1–10. Fig S11: Yao’s dataset, myotubes: Active modules 1–23. Fig S12: Banerji’s 2017 dataset: Active modules 1–23. Fig S13: Banerji’s 2019 dataset: Active modules 1–17. 2. Supplementary Tables. Table S1: Samples from Yao’s datasets. Downloaded from https://www.ncbi.nlm.nih.gov/geo/query/acc.cgi?acc=GSE56787. Table S2: Samples from Banerji’s 2017 dataset. Downloaded from https://www.ncbi.nlm.nih.gov/geo/query/acc.cgi?acc=GSE102812. Table S3: Samples from Banerji’s 2019 dataset. Downloaded from https://www.ncbi.nlm.nih.gov/geo/query/acc.cgi?acc=GSE123468. 3. Non-dominated Sorting Genetic Algorithm II (NSGA-II). Algo [file pcbi.1009263.s001.pdf]

A Multi-Objective Genetic Algorithm to Find Active Modules in Multiplex Biological Networks: Supplementary material

Elva María Novoa-del-Toro[1,*] Efrén Mezura-Montes[2] Matthieu Vignes[3]
Morgane Térézol [1] Frédérique Magdinier[1] Laurent Tichit[4] Anaïs Baudot[1,5,*]

[1]Aix Marseille Univ, INSERM, Marseille Medical Genetics (MMG), Marseille, France
[2]University of Veracruz, Artificial Intelligence Research Center, Mexico
[3]School of Fundamental Sciences, Massey University, Palmerston North, New Zealand
[4]Aix Marseille Univ, CNRS, Centale Marseille, I2M UMR 7373, Marseille, France
[5]Barcelona Supercomputing Center, Barcelona, Spain
[*]To whom correspondence should be addressed

Contents

1 Supplementary Figs **2**
Fig S1 3
Fig S2 3
Fig S3 4
Fig S4 4
Fig S5 5
Fig S6 5
Fig S7 6
Fig S8 6
Fig S9 6
Fig S10 14
Fig S11 18
Fig S12 30
Fig S13 38

2 Supplementary Tables **48**
Table S1 49
Table S2 50
Table S3 50

3 Non-dominated Sorting Genetic Algorithm II (NSGA-II) **50**
Algorithm S1 50
Fig S14 52
Algorithm S2 52
Algorithm S3 52

4 MOGAMUN Genetic Algorithm parameter tuning **53**
Fig S15 54
Fig S16 55

5 Application to Facio-Scapulo-Humeral muscular Dystrophy type 1 (FSHD1) 55
5.1 Distribution of size, density and average nodes score obtained by the different methods 56
5.1.1 Analyses of Yao's dataset, biopsies 56
Fig S17 57
Fig S18 58
Fig S19 59
5.1.2 Analyses of Yao's dataset, myotubes 59
Fig S20 60
Fig S21 61
Fig S22 62
5.1.3 Analyses of Yao's dataset, myoblasts 62
Fig S23 63
Fig S24 63
Fig S25 64
5.2 Active modules nodes statistics 64
Table S4 65
Table S5 65

1 Supplementary Figs

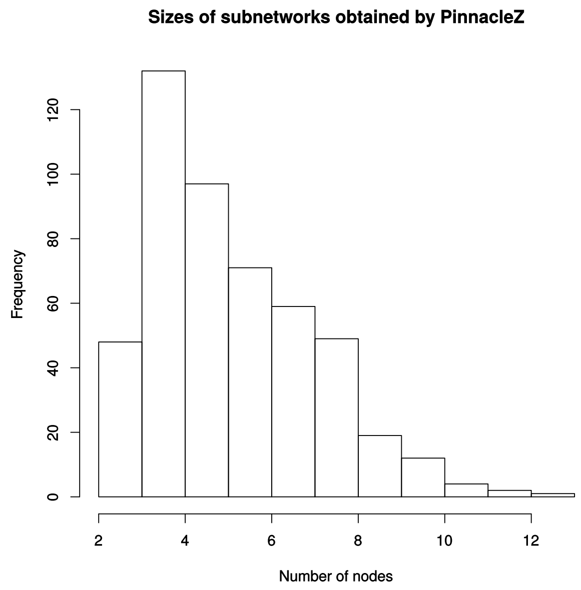

Fig S1: Sizes of the subnetworks identified by PinnacleZ in the experiment using the network *PPI_1* and the simulated data with normal distribution

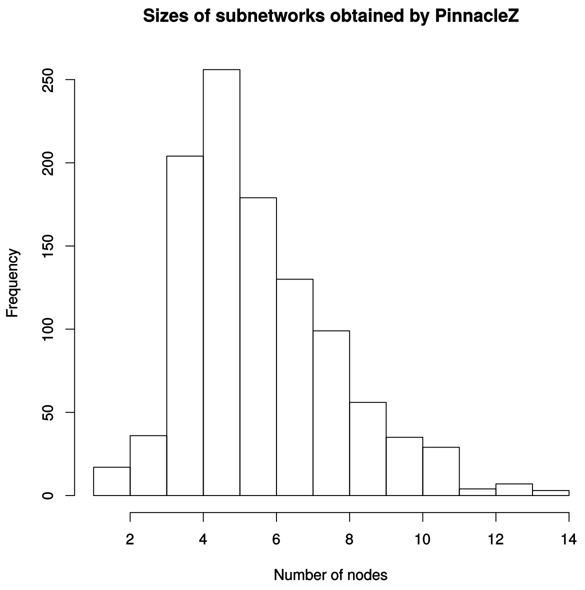

Fig S2: Sizes of the subnetworks identified by PinnacleZ in the experiment using the network *PPI_2* and the sampled data from RNA-Seq TCGA breast cancer dataset

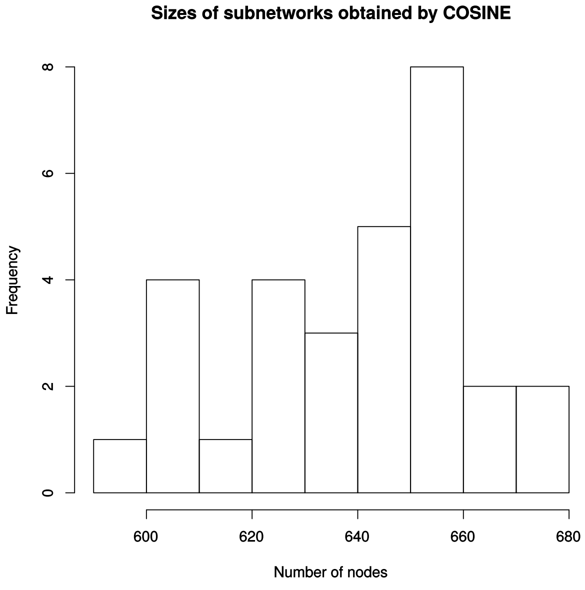

Fig S3: Sizes of the subnetworks identified by COSINE in the experiment using the network *PPI_1* and the simulated data with normal distribution

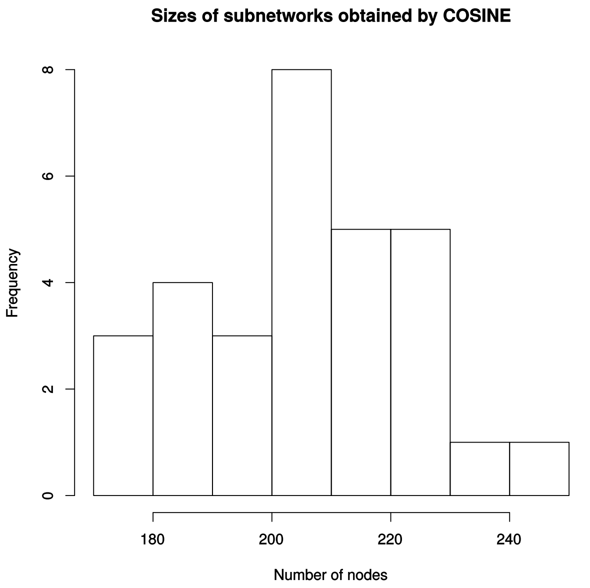

Fig S4: Sizes of the subnetworks identified by COSINE in the experiment using the network *PPI_2* and the sampled data from RNA-Seq TCGA breast cancer dataset

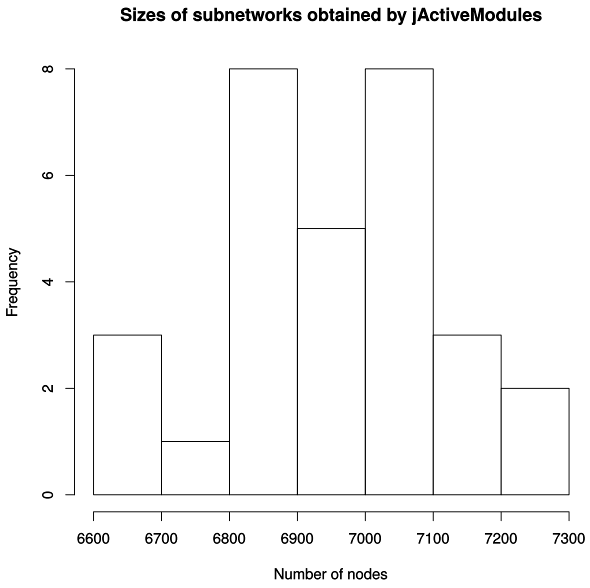

Fig S5: Sizes of the subnetworks identified by jActiveModules in the experiment using the network *PPI_1* and the simulated data with normal distribution

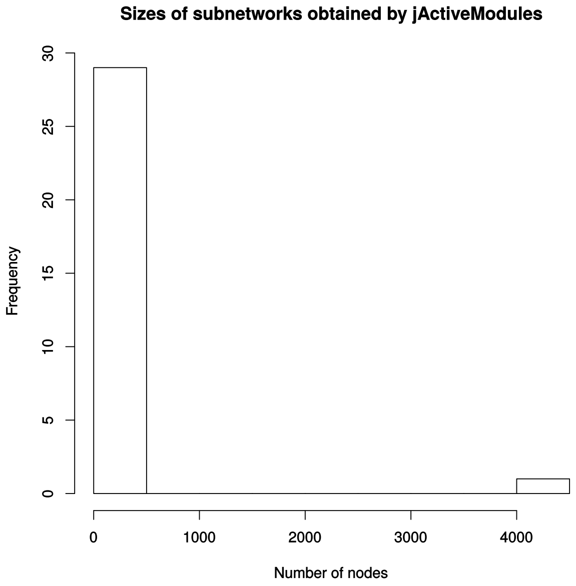

Fig S6: Sizes of the subnetworks identified by jActiveModules in the experiment using the network *PPI_2* and the sampled data from RNA-Seq TCGA breast cancer dataset

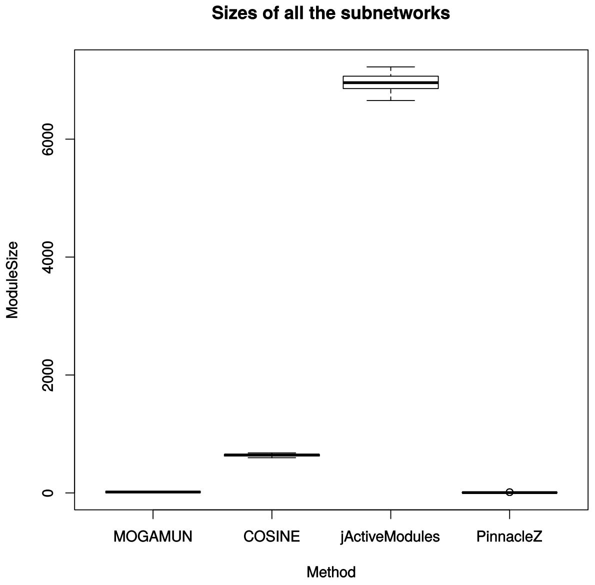

Fig S7: Sizes of the subnetworks identified by all the methods in the experiment using the network *PPI_1* and the simulated data with normal distribution

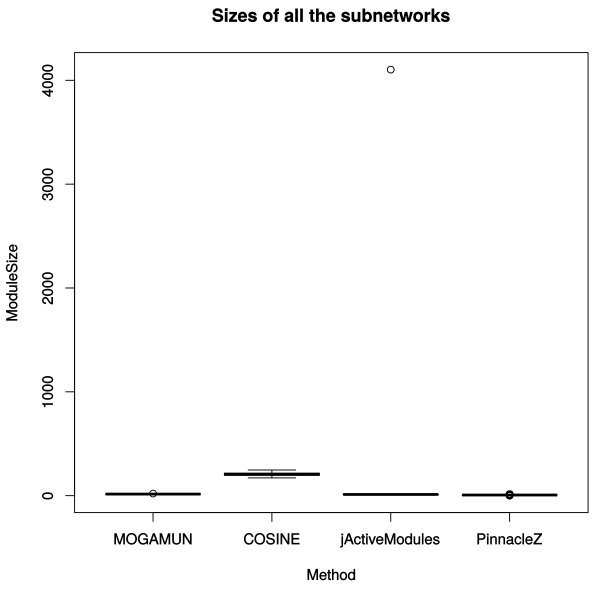

Fig S8: Sizes of the subnetworks identified by all the methods in the experiment using the network *PPI_2* and the sampled data from RNA-Seq TCGA breast cancer dataset

Fig S9

We obtained eighteen active modules by applying MOGAMUN to the Yao's dataset, biopsies [1] (see Table S1 for the list of samples). The color of the nodes encodes the fold-change, where green and red nodes correspond to under- and over-expressed genes, respectively. The background node color intensity denotes the strength of the deregulation. Nodes with bold black border correspond to genes significantly differentially expressed (FDR < 0.05 and absolute log_2 fold-change > 1). Blue and white nodes correspond to genes with no associated transcriptomics data and no deregulation, respectively. The color of the edges represents the layer of the multiplex network, where blue, orange, and yellow correspond to PPI, Pathways, and Co-expression, respectively.

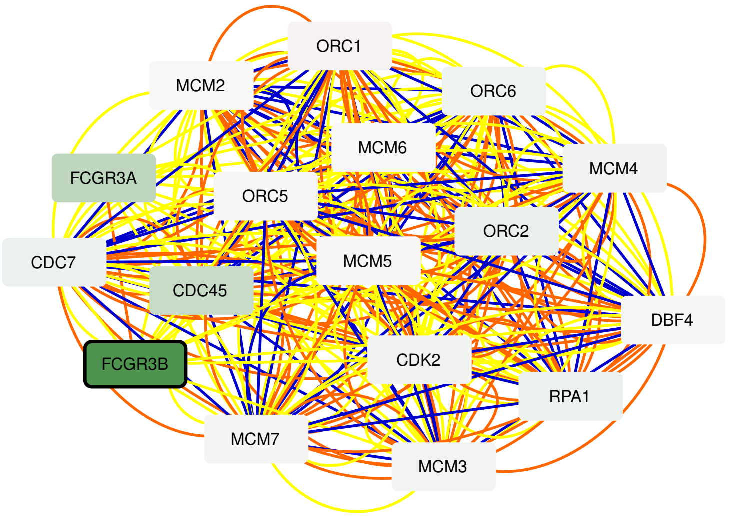

Fig S9.1: Yao's dataset, biopsies: Active module 1

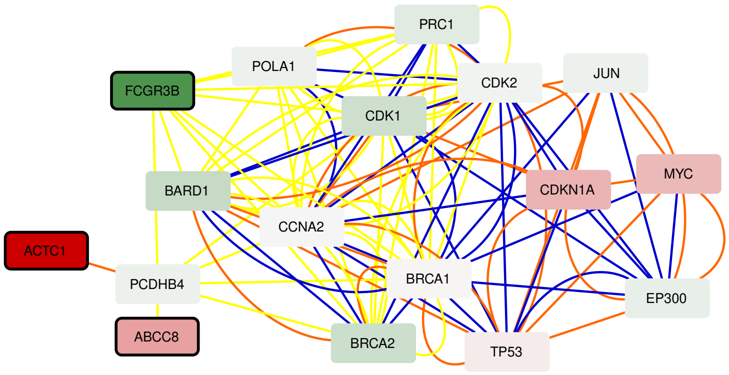

Fig S9.2: Yao's dataset, biopsies: Active module 2

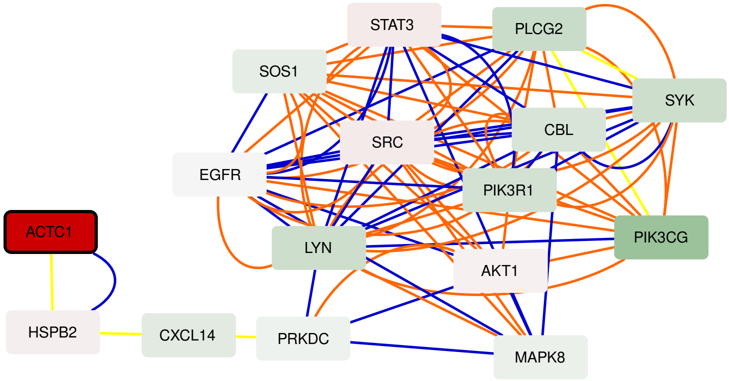

Fig S9.3: Yao's dataset, biopsies: Active module 3

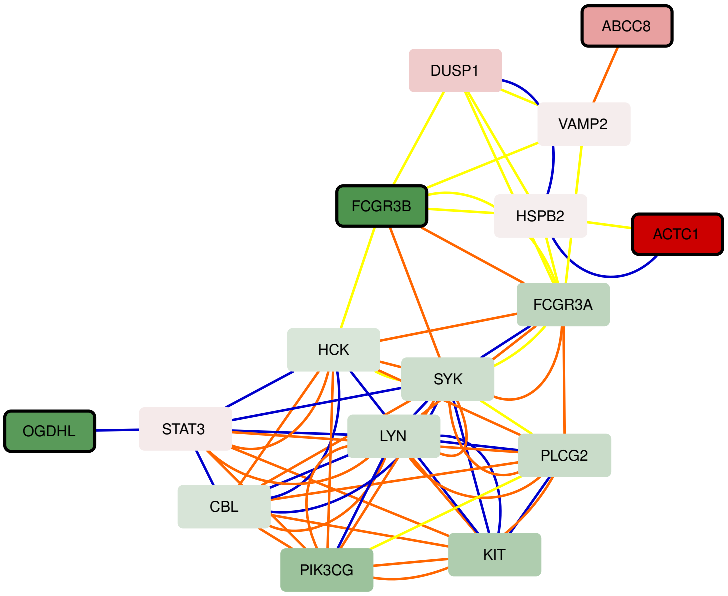

Fig S9.4: Yao's dataset, biopsies: Active module 4

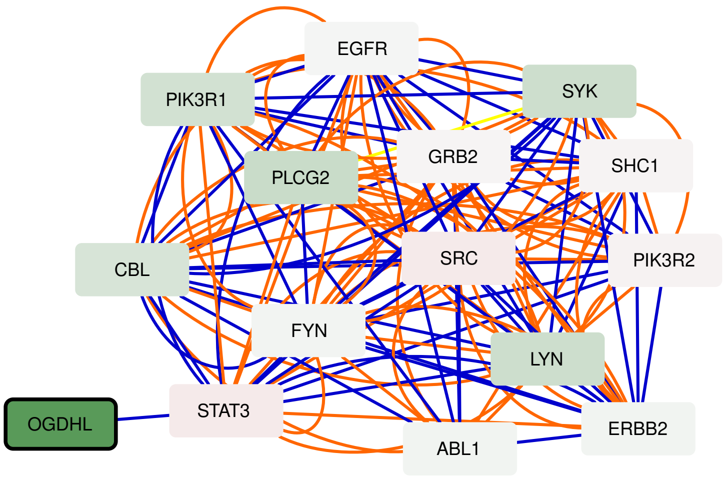

Fig S9.5: Yao's dataset, biopsies: Active module 5

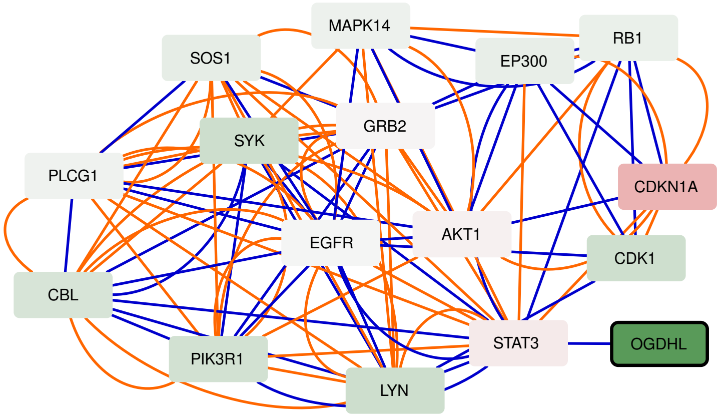

Fig S9.6: Yao's dataset, biopsies: Active module 6

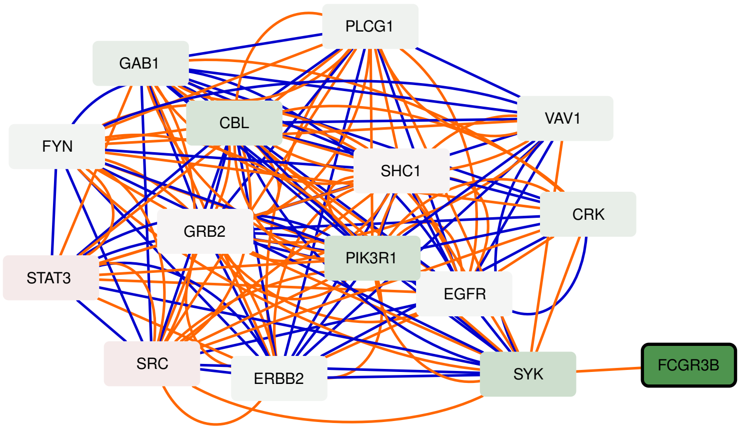

Fig S9.7: Yao's dataset, biopsies: Active module 7

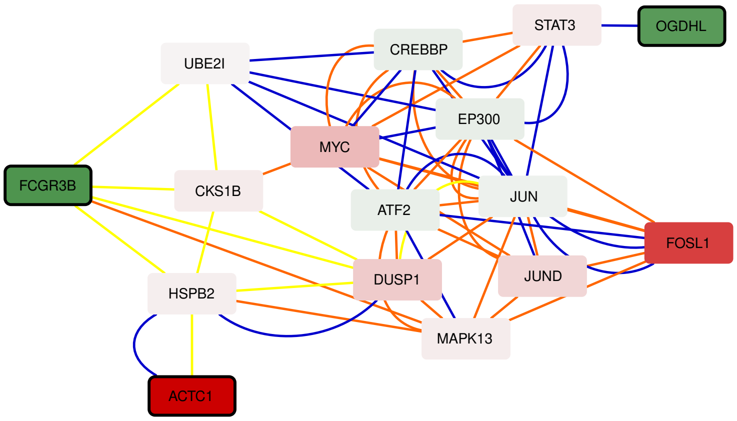

Fig S9.8: Yao's dataset, biopsies: Active module 8

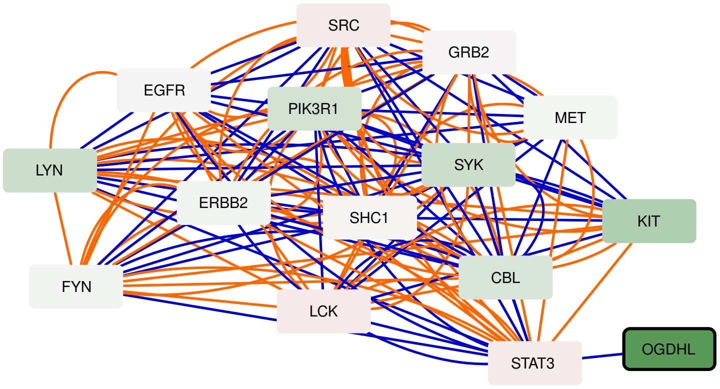

Fig S9.9: Yao's dataset, biopsies: Active module 9

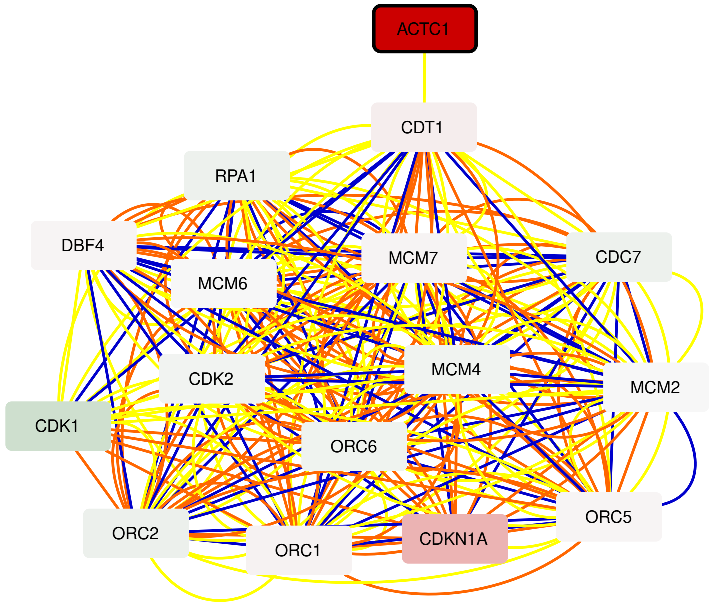

Fig S9.10: Yao's dataset, biopsies: Active module 10

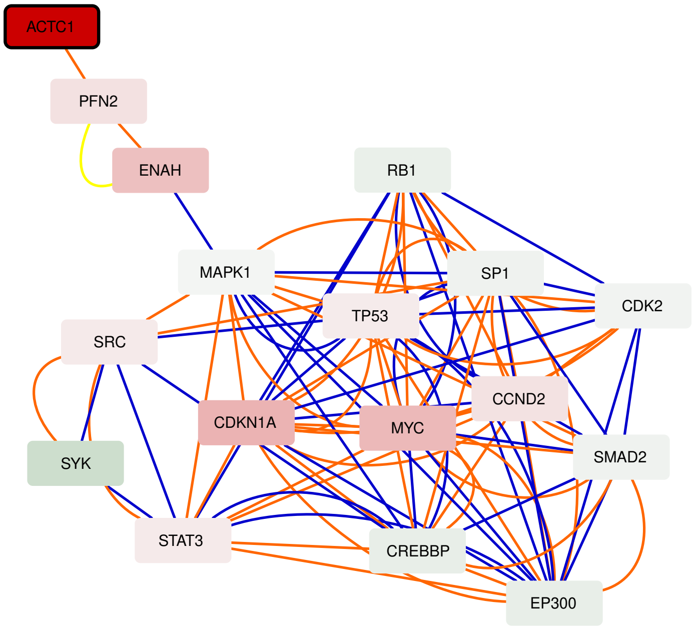

Fig S9.11: Yao's dataset, biopsies: Active module 11

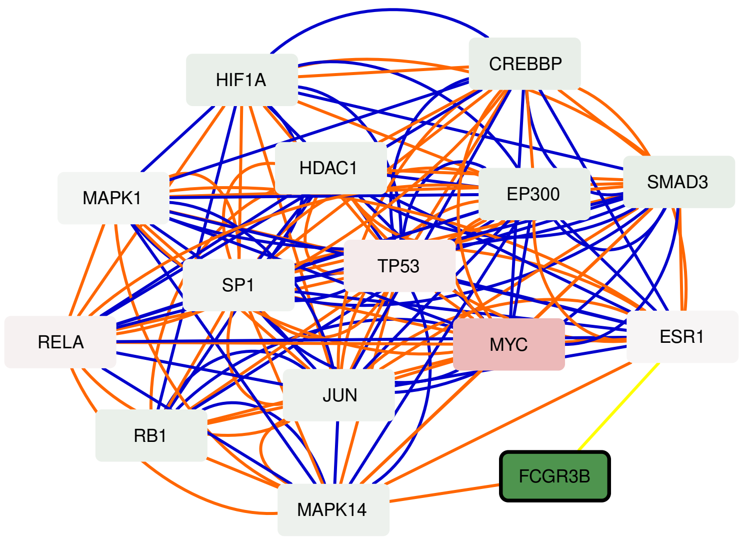

Fig S9.12: Yao's dataset, biopsies: Active module 12

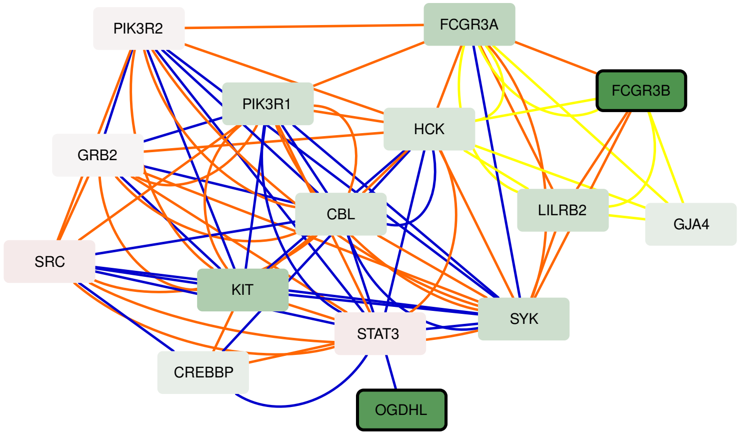

Fig S9.13: Yao's dataset, biopsies: Active module 13

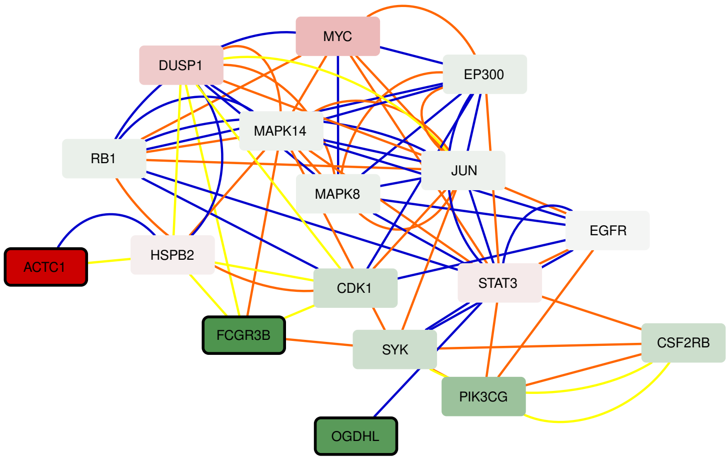

Fig S9.14: Yao's dataset, biopsies: Active module 14

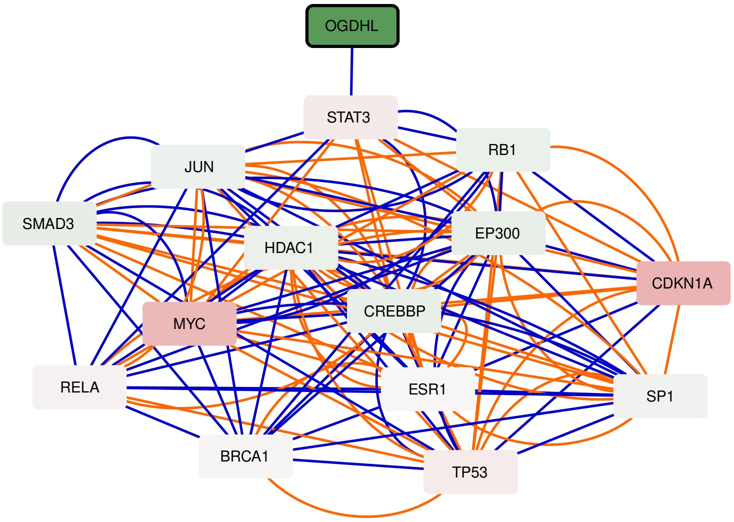

Fig S9.15: Yao's dataset, biopsies: Active module 15

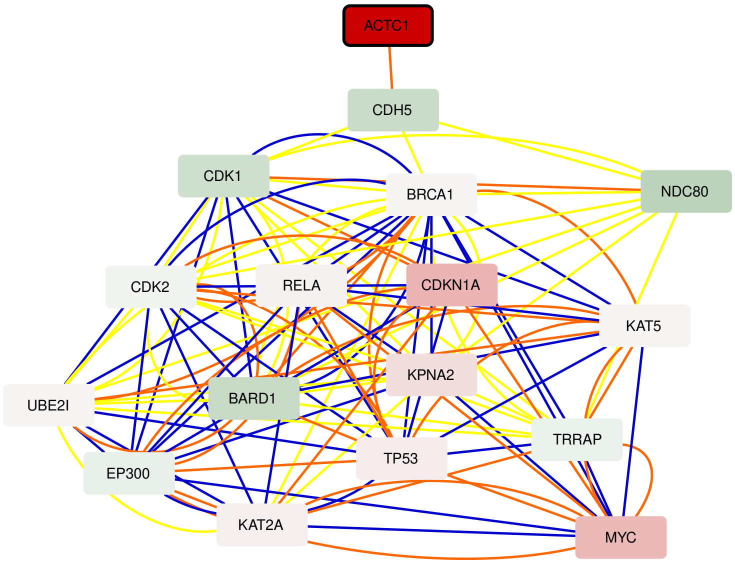

Fig S9.16: Yao's dataset, biopsies: Active module 16

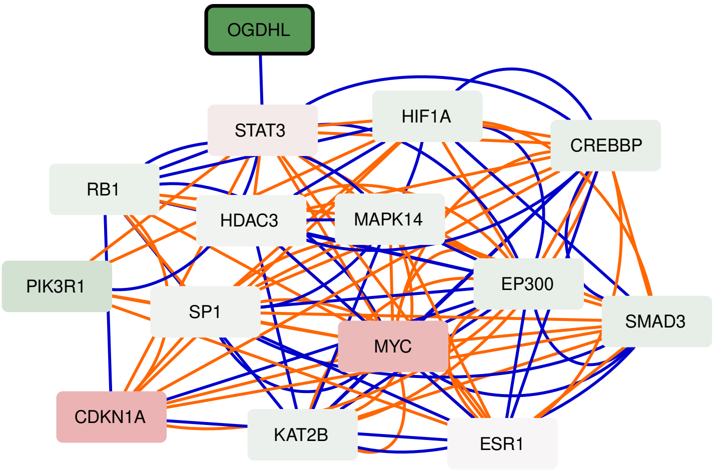

Fig S9.17: Yao's dataset, biopsies: Active module 17

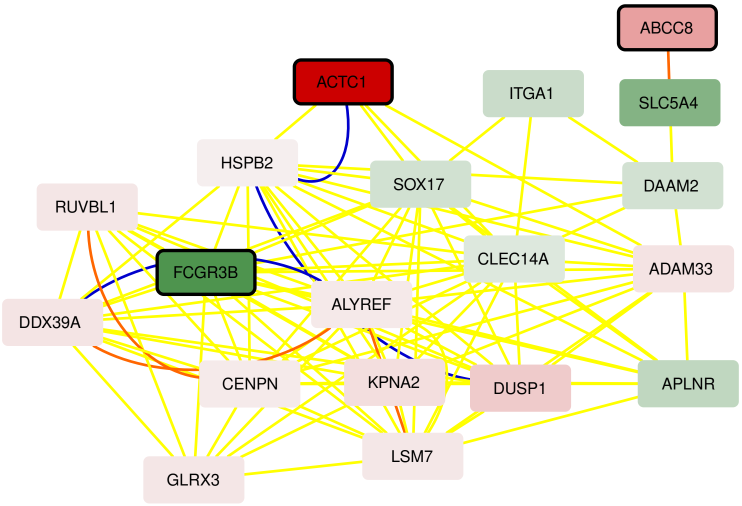

Fig S9.18: Yao's dataset, biopsies: Active module 18

Fig S10

We obtained ten active modules by applying MOGAMUN to the Yao's dataset, myoblasts [1] (see Table S1 for the list of samples). The color of the nodes represents the fold-change, where green and red nodes correspond to under- and over-expressed genes, respectively. The background node color intensity denotes the strength of the deregulation. Nodes with bold black border correspond to genes significantly differentially expressed (FDR < 0.05 and absolute log_2 fold-change > 1). Blue and white nodes correspond to genes with no associated transcriptomics data and no deregulation, respectively. The color of the edges represents the layer of the multiplex network, where blue, orange, and yellow correspond to PPI, Pathways, and Co-expression, respectively.

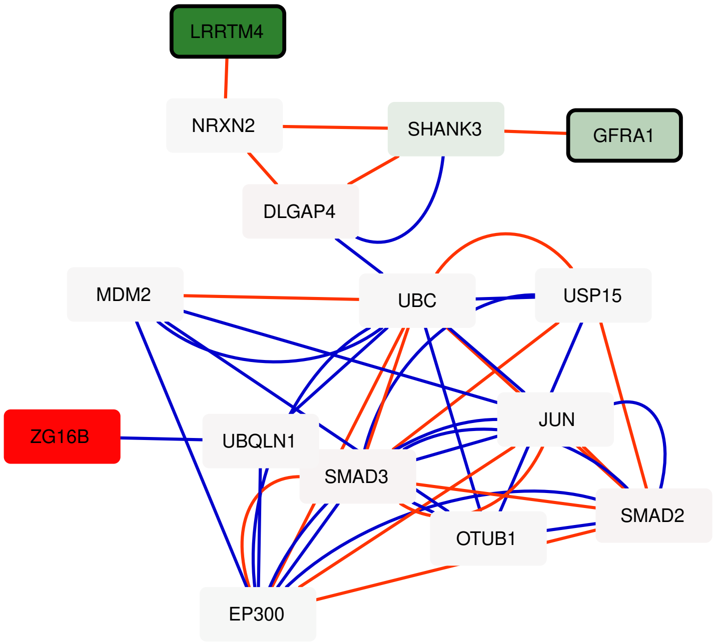

Fig S10.1: Yao's dataset, myoblasts: Active module 1

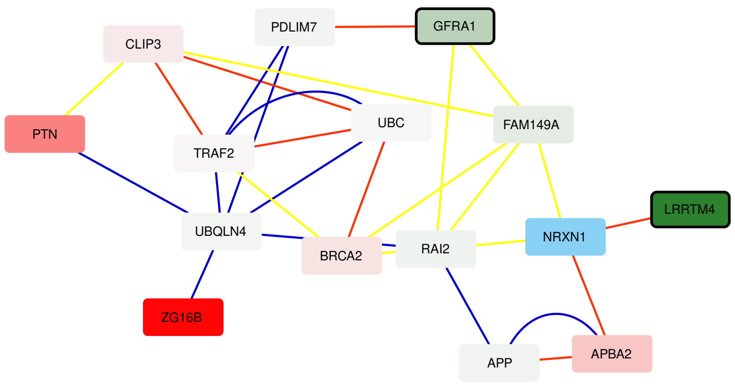

Fig S10.2: Yao's dataset, myoblasts: Active module 2

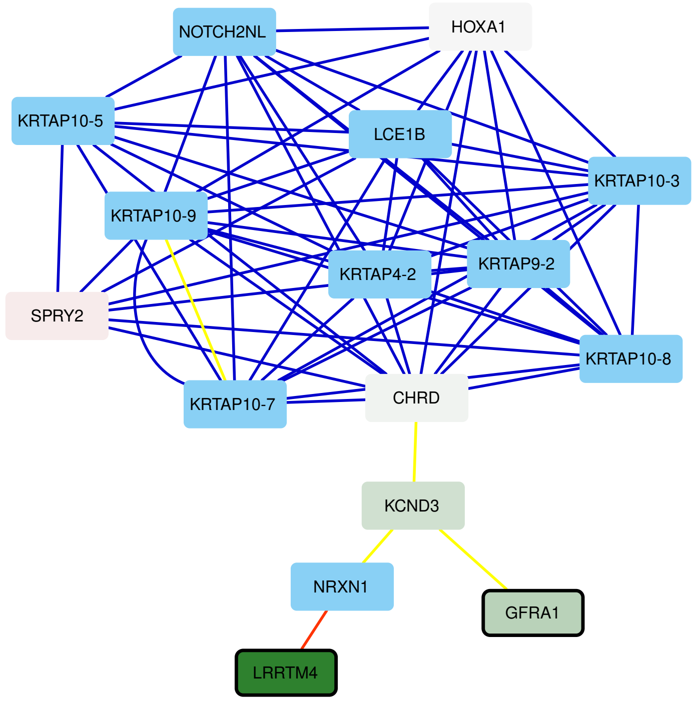

Fig S10.3: Yao's dataset, myoblasts: Active module 3

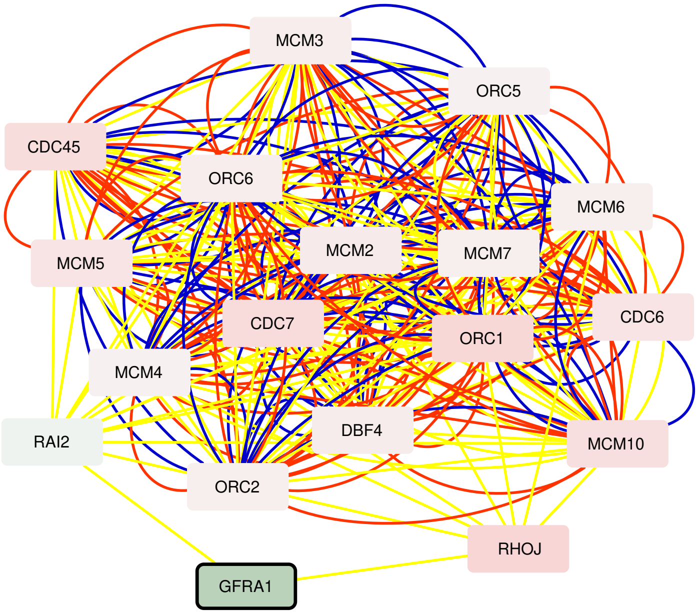

Fig S10.4: Yao's dataset, myoblasts: Active module 4

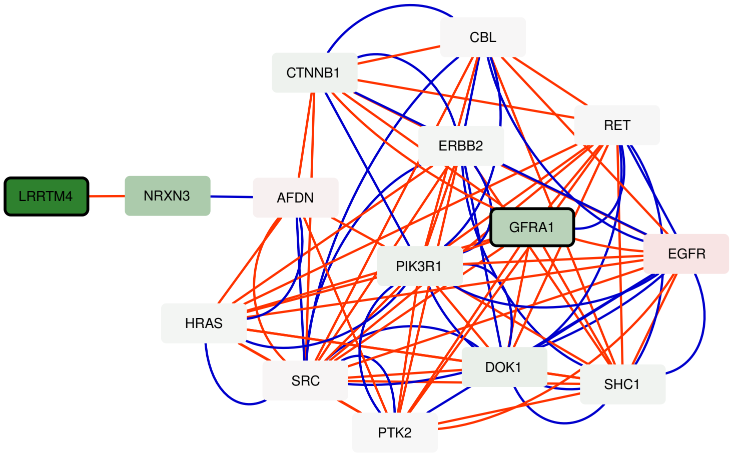

Fig S10.5: Yao's dataset, myoblasts: Active module 5

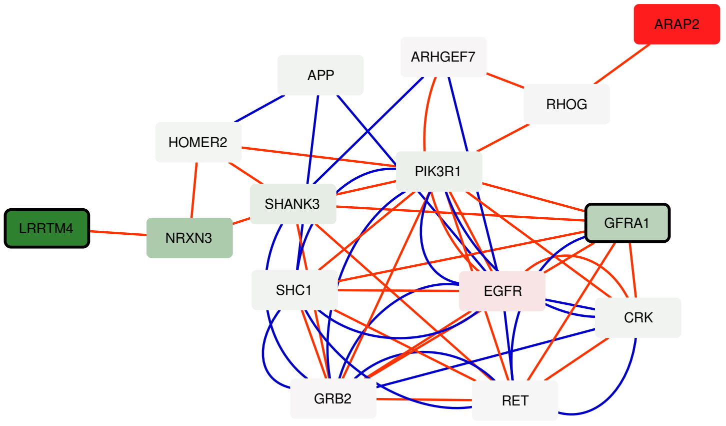

Fig S10.6: Yao's dataset, myoblasts: Active module 6

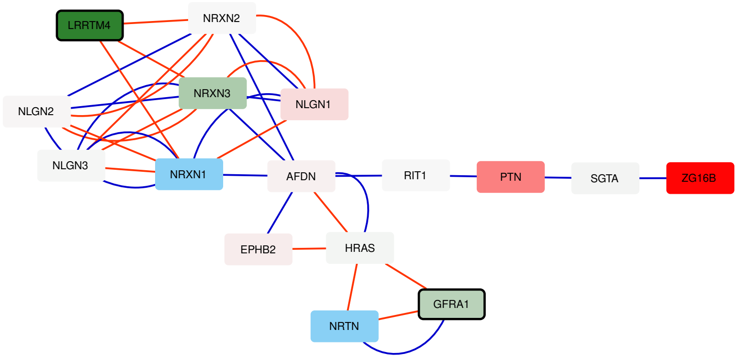

Fig S10.7: Yao's dataset, myoblasts: Active module 7

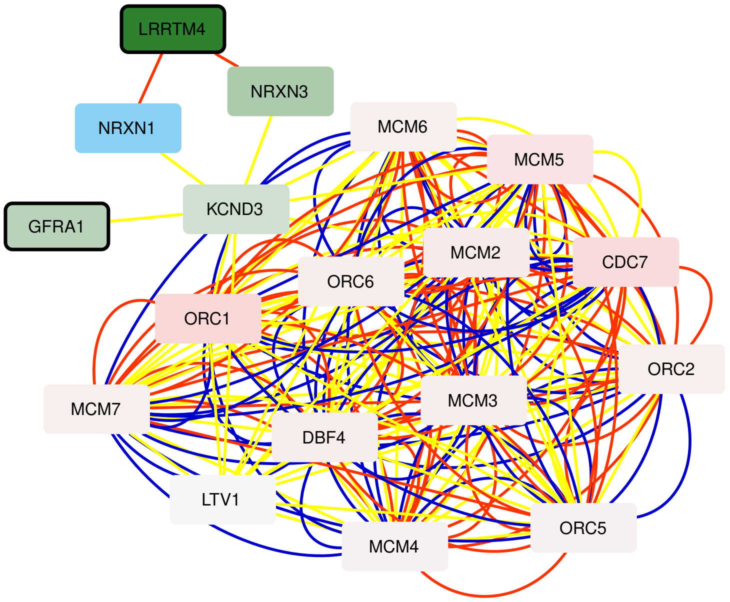

Fig S10.8: Yao's dataset, myoblasts: Active module 8

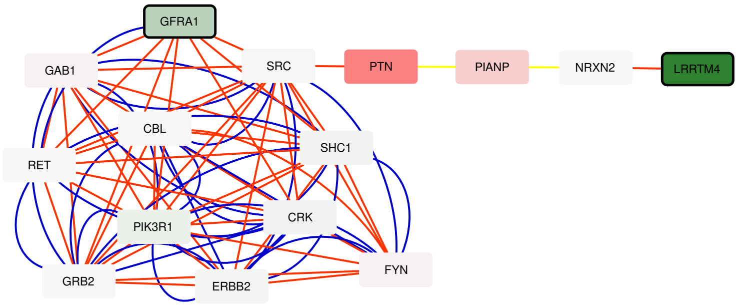

Fig S10.9: Yao's dataset, myoblasts: Active module 9

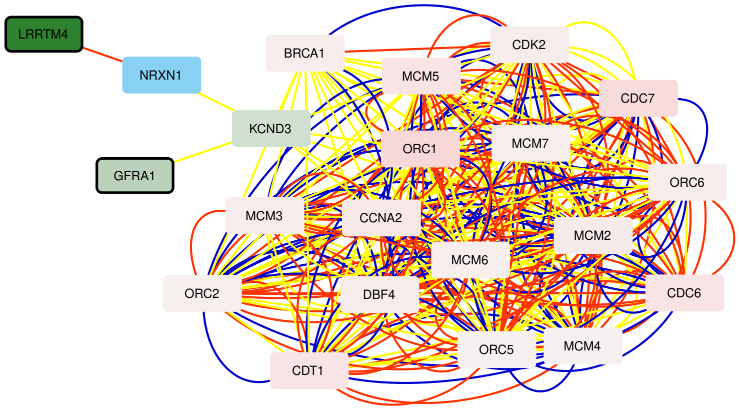

Fig S10.10:. Yao's dataset, myoblasts: Active module 10

Fig S11

We obtained twenty three active modules by applying MOGAMUN to the Yao's dataset, myotubes [1] (see Table S1 for the list of samples). The color of the nodes represents the fold-change, where green and red nodes correspond to under- and over-expressed genes, respectively. The background node color intensity denotes the strength of the deregulation. Nodes with bold black border correspond to genes significantly differentially expressed (FDR < 0.05 and absolute log_2 fold-change > 1). Blue and white nodes correspond to genes with no associated transcriptomics data and no deregulation, respectively. The color of the edges represents the layer of the multiplex network, where blue, orange, and yellow correspond to PPI, Pathways, and Co-expression, respectively.

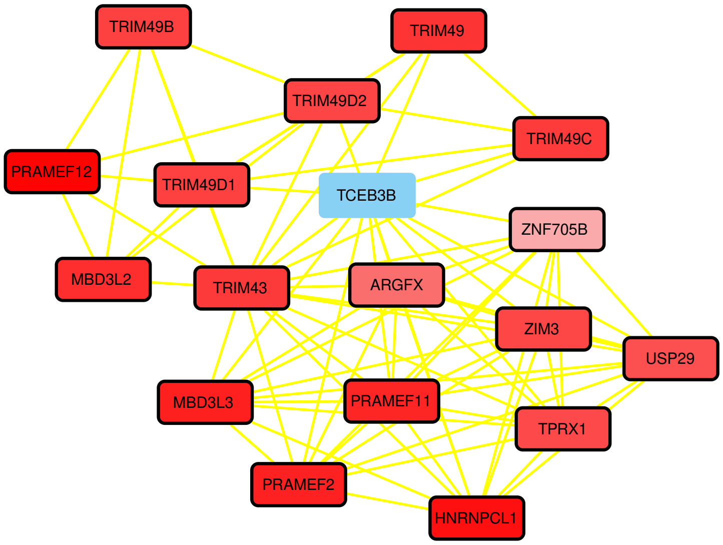

Fig S11.1: Yao's dataset, myotubes: Active module 1

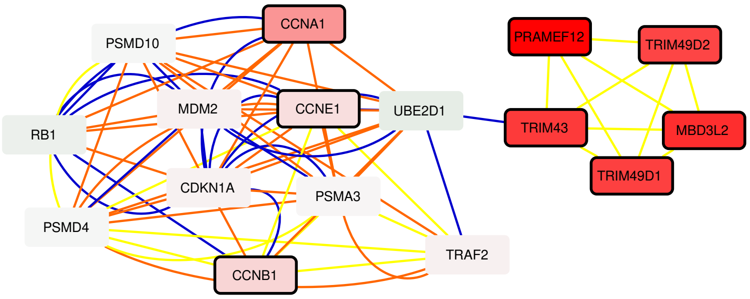

Fig S11.2: Yao's dataset, myotubes: Active module 2

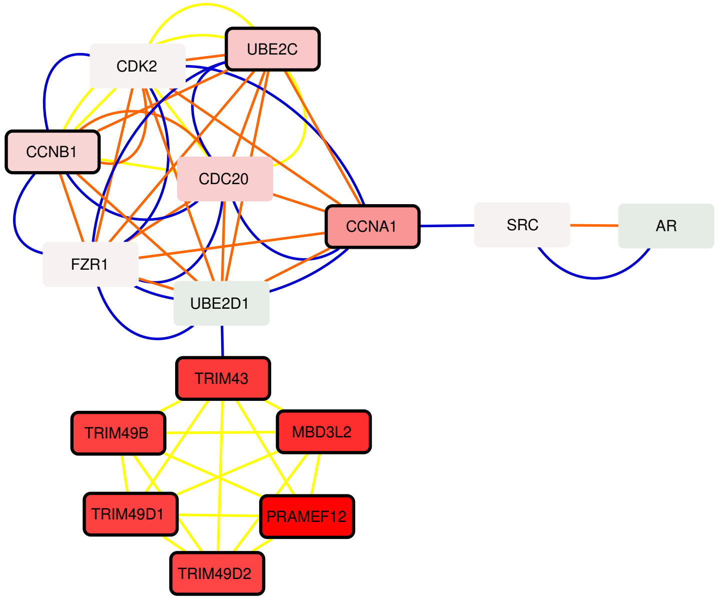

Fig S11.3: Yao's dataset, myotubes: Active module 3

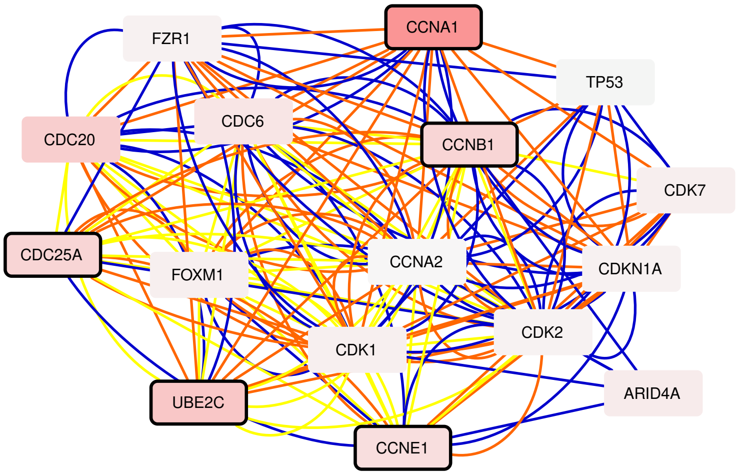

Fig S11.4: Yao's dataset, myotubes: Active module 4

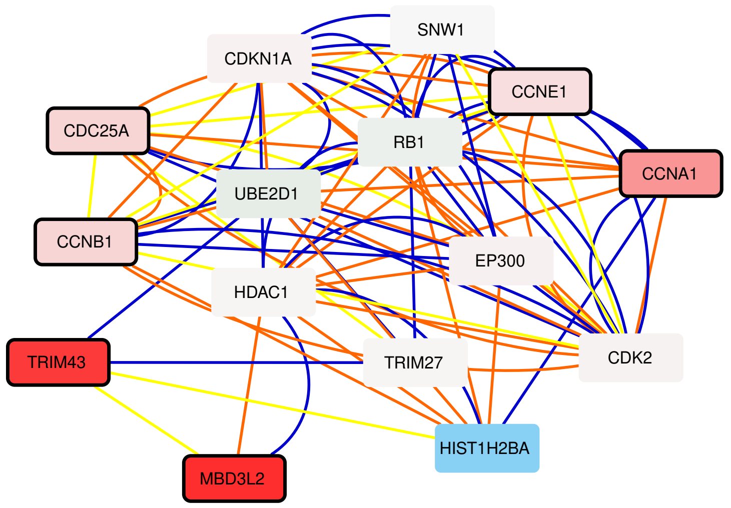

Fig S11.5: Yao's dataset, myotubes: Active module 5

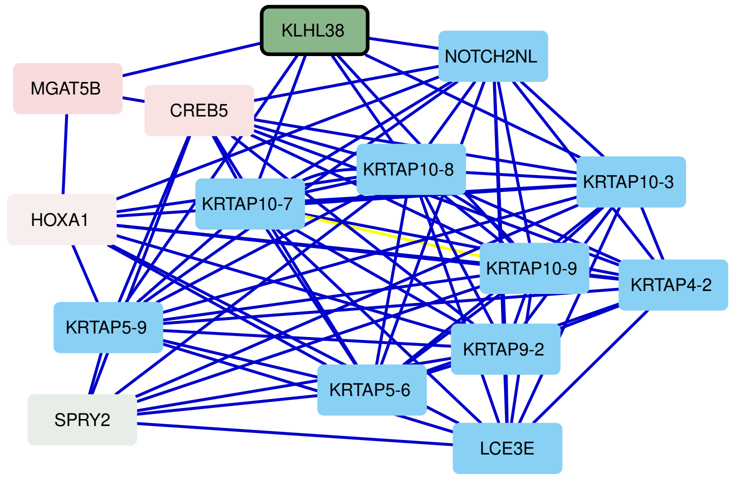

Fig S11.6: Yao's dataset, myotubes: Active module 6

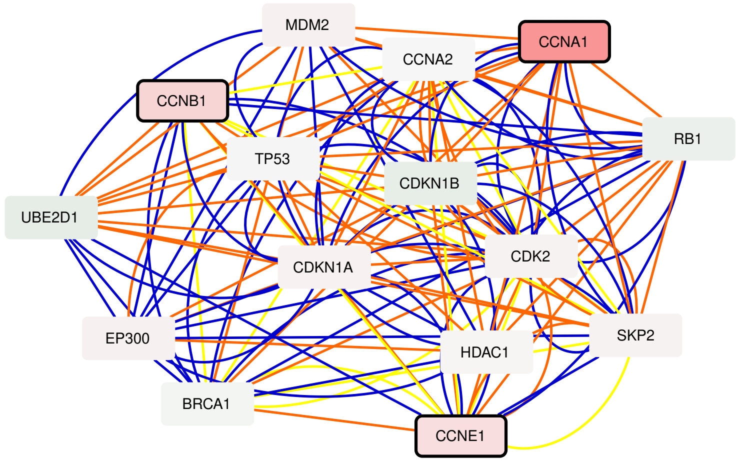

Fig S11.7: Yao's dataset, myotubes: Active module 7

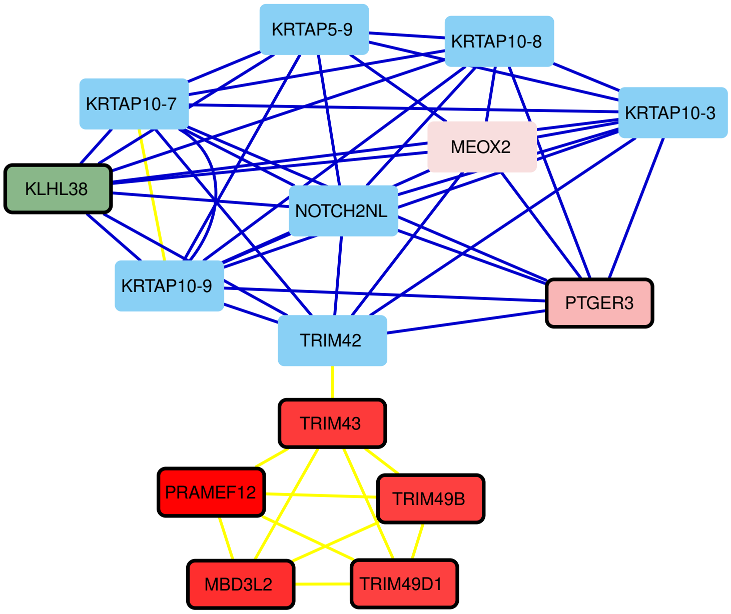

Fig S11.8: Yao's dataset, myotubes: Active module 8

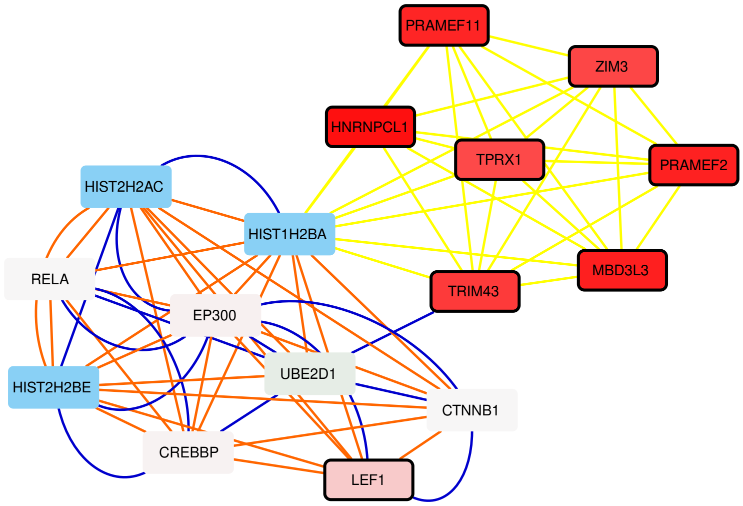

Fig S11.9: Yao's dataset, myotubes: Active module 9

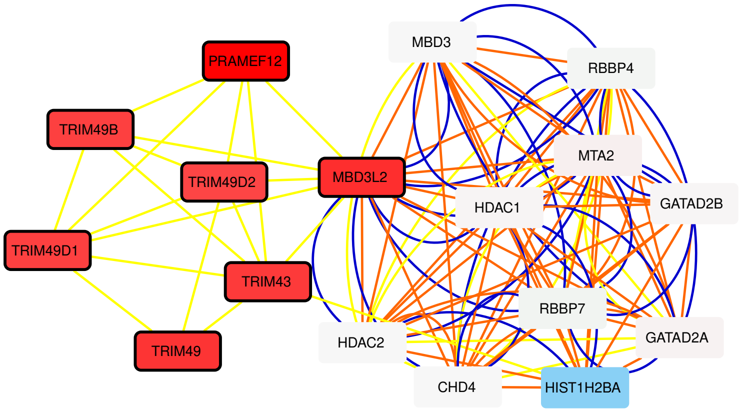

Fig S11.10: Yao's dataset, myotubes: Active module 10

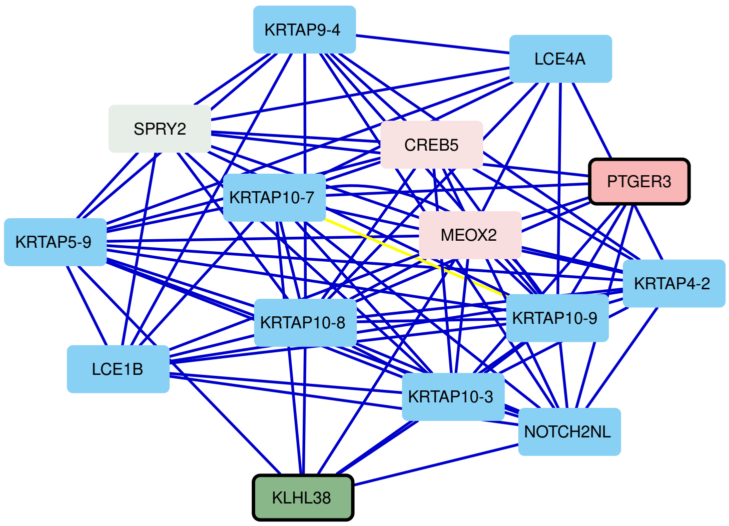

Fig S11.11: Yao's dataset, myotubes: Active module 11

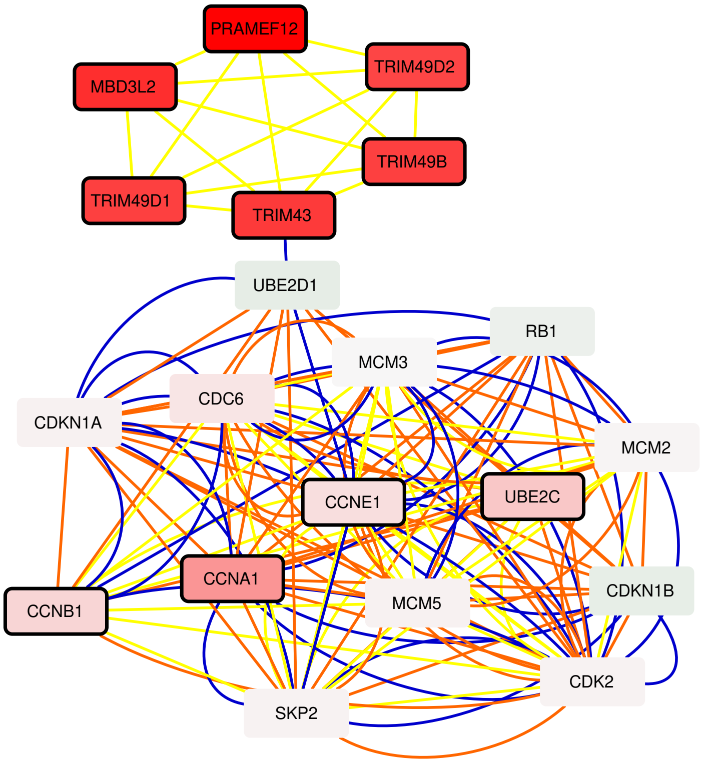

Fig S11.12: Yao's dataset, myotubes: Active module 12

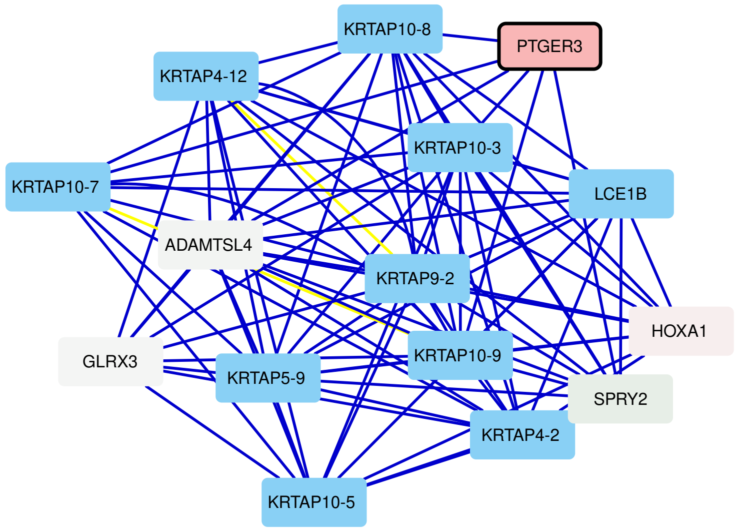

Fig S11.13: Yao's dataset, myotubes: Active module 13

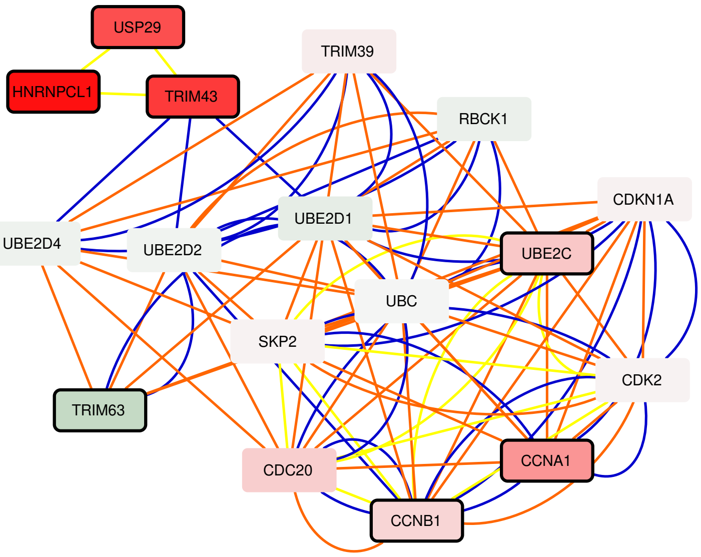

Fig S11.14: Yao's dataset, myotubes: Active module 14

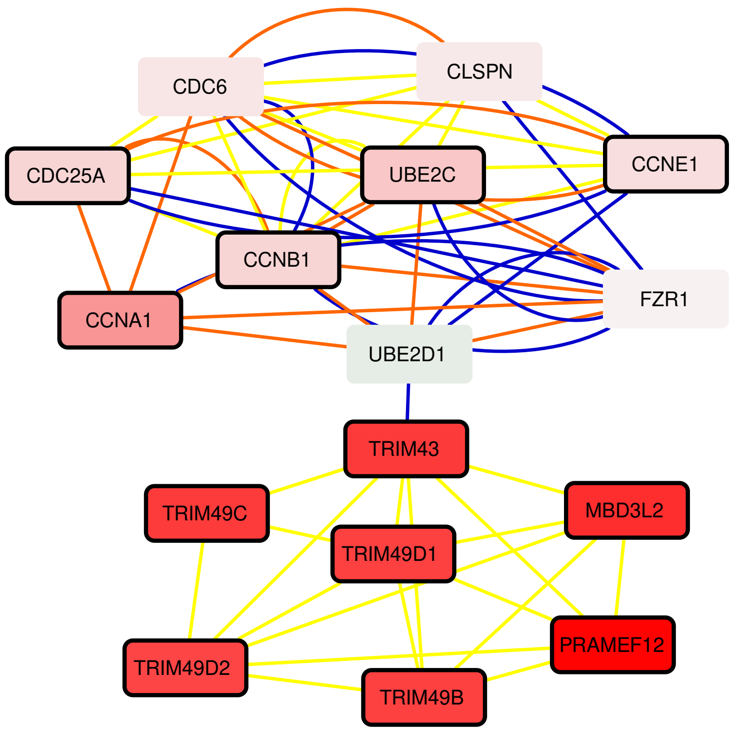

Fig S11.15: Yao's dataset, myotubes: Active module 15

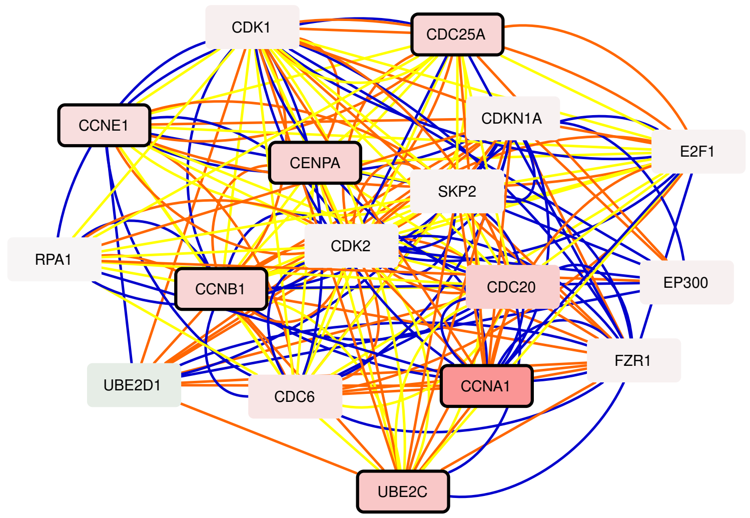

Fig S11.16: Yao's dataset, myotubes: Active module 16

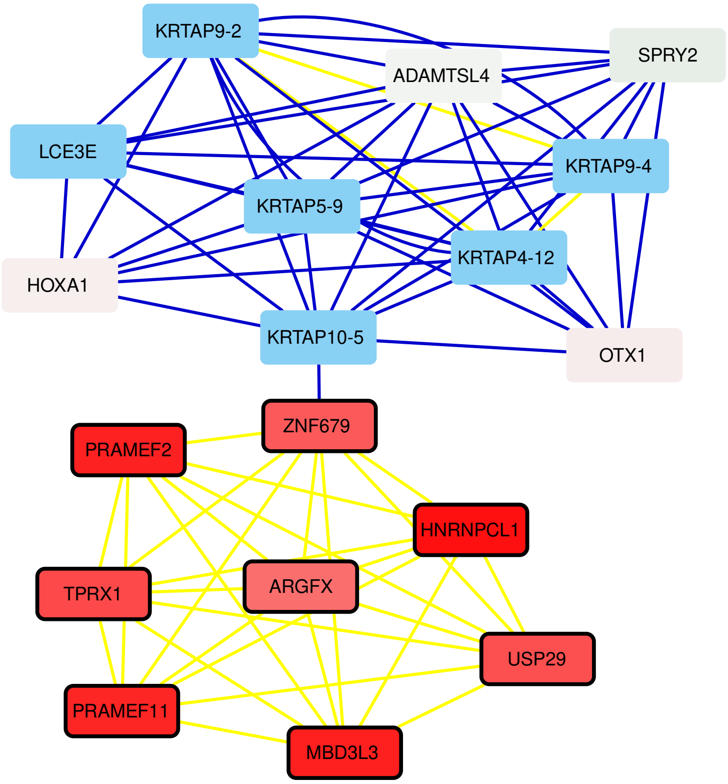

Fig S11.17: Yao's dataset, myotubes: Active module 17

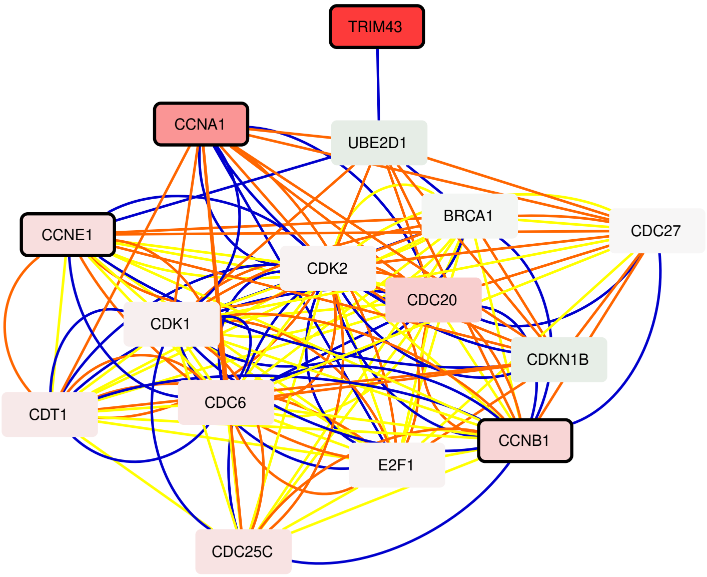

Fig S11.18: Yao's dataset, myotubes: Active module 18

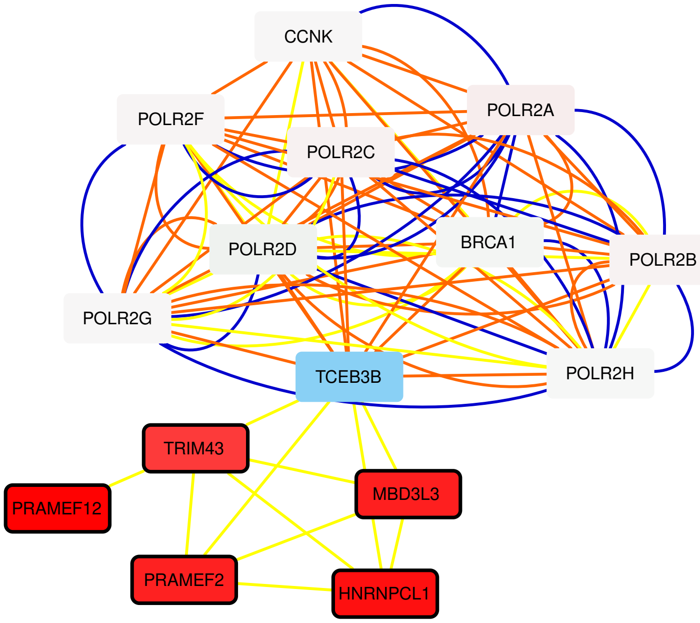

Fig S11.19: Yao's dataset, myotubes: Active module 19

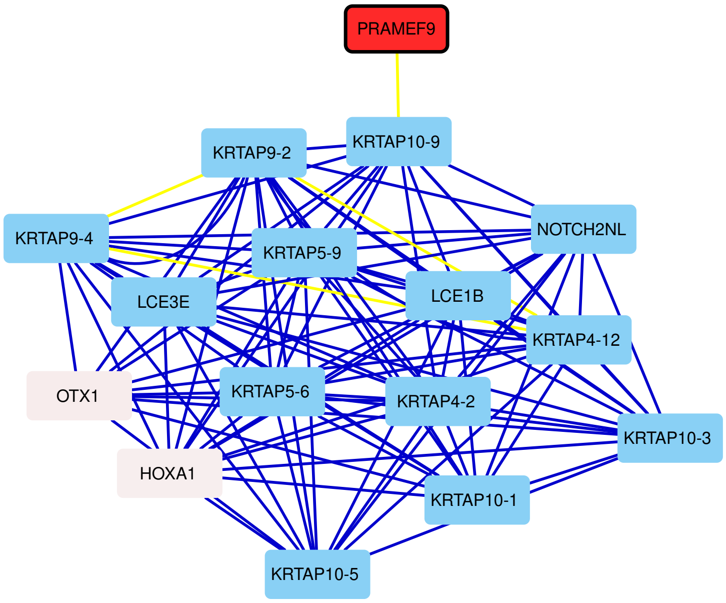

Fig S11.20: Yao's dataset, myotubes: Active module 20

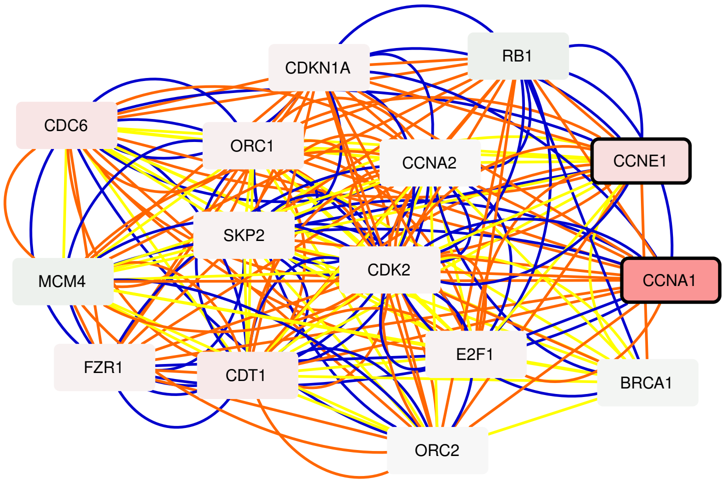

Fig S11.21: Yao's dataset, myotubes: Active module 21

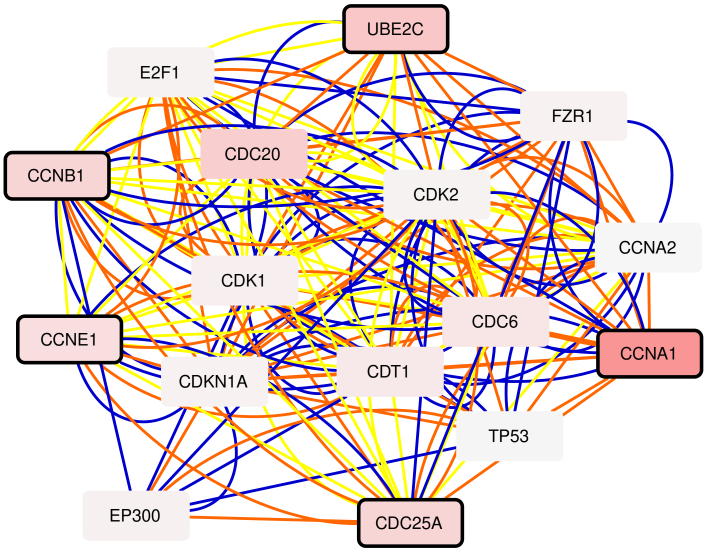

Fig S11.22: Yao's dataset, myotubes: Active module 22

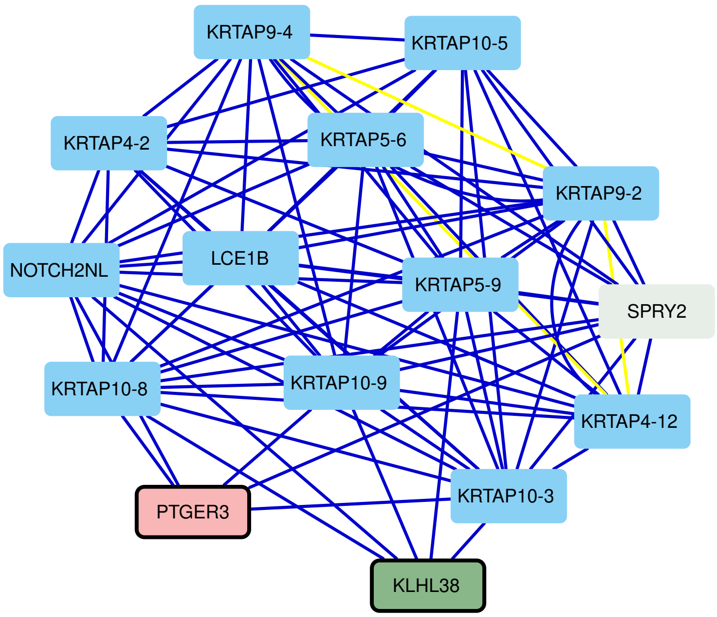

Fig S11.23: Yao's dataset, myotubes: Active module 23

Fig S12

We obtained twenty three active modules obtained by applying MOGAMUN to the Banerji's 2017 dataset [2] (see Table S2 for the list of samples). The color of the nodes represents the fold-change, where green and red nodes correspond to under- and over-expressed genes, respectively. The background node color intensity denotes the strength of the deregulation. Nodes with bold black border correspond to genes significantly differentially expressed (FDR < 0.05 and absolute log_2 fold-change > 1). Blue and white nodes correspond to genes with no associated transcriptomics data and no deregulation, respectively. The color of the edges represents the layer of the multiplex network, where blue, orange, and yellow correspond to PPI, Pathways, and Co-expression, respectively.

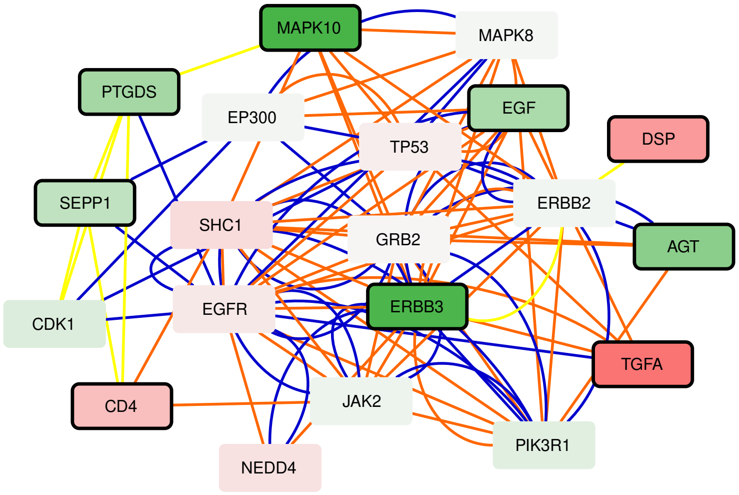

Fig S12.1: Banerji's 2017 dataset: Active module 1

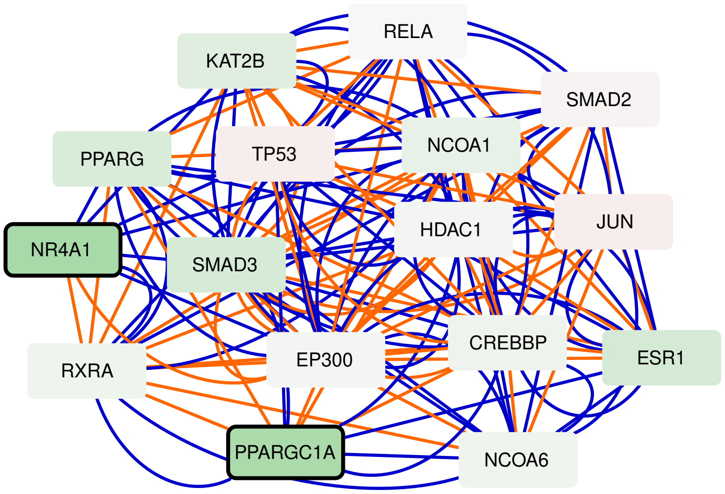

Fig S12.2: Banerji's 2017 dataset: Active module 2

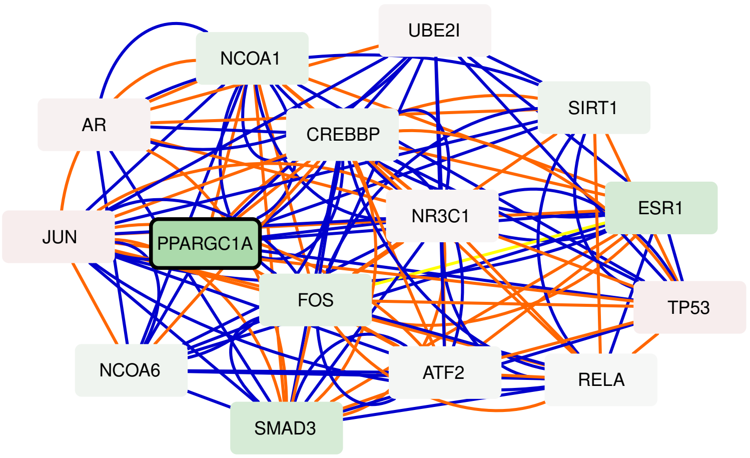

Fig S12.3: Banerji's 2017 dataset: Active module 3

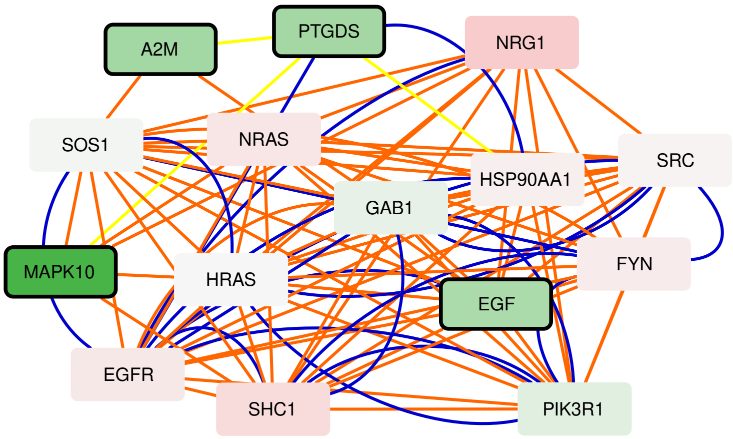

Fig S12.4: Banerji's 2017 dataset: Active module 4

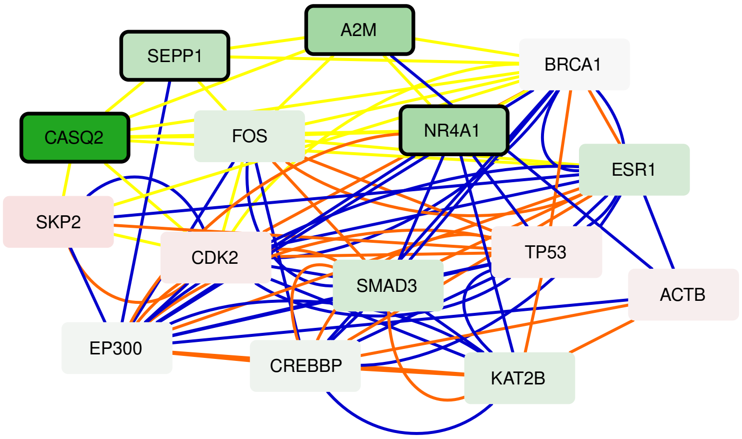

Fig S12.5: Banerji's 2017 dataset: Active module 5

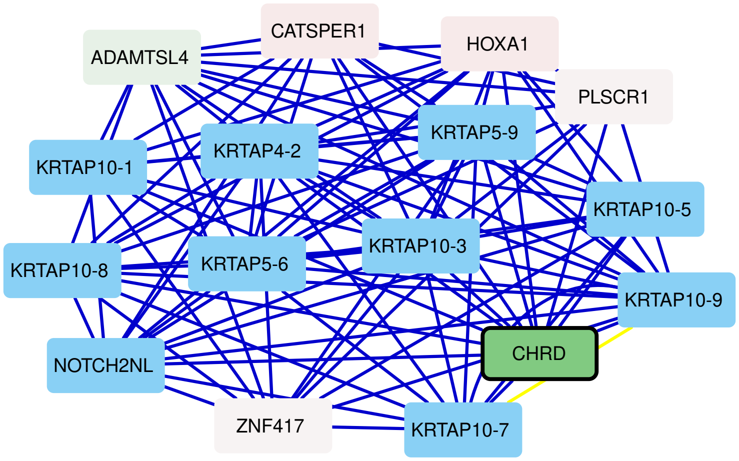

Fig S12.6: Banerji's 2017 dataset: Active module 6

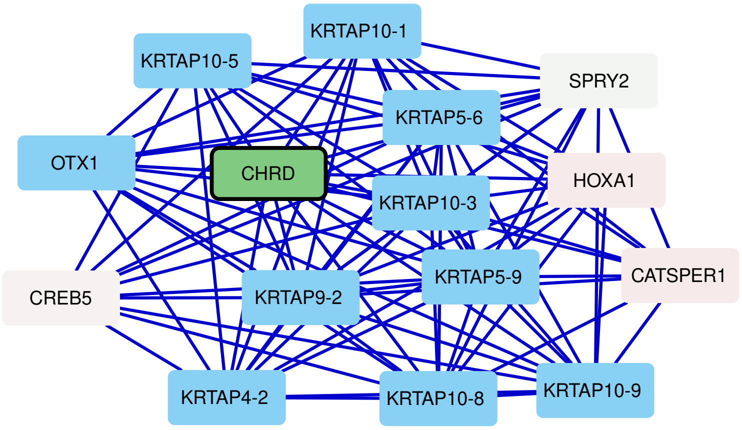

Fig S12.7: Banerji's 2017 dataset: Active module 7

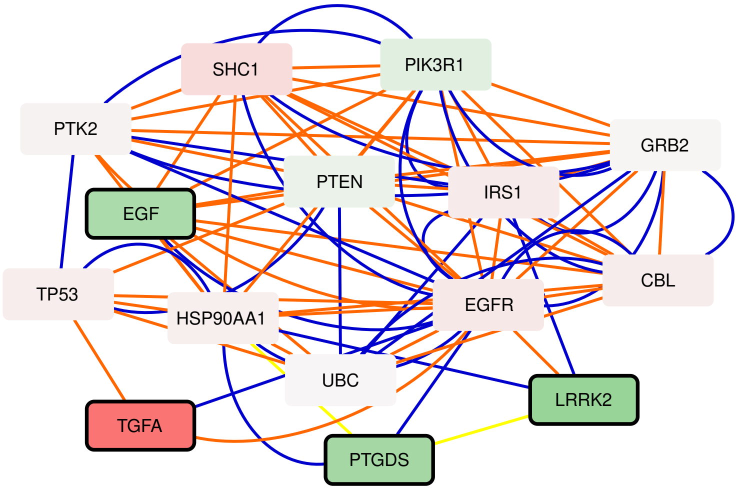

Fig S12.8: Banerji's 2017 dataset: Active module 8

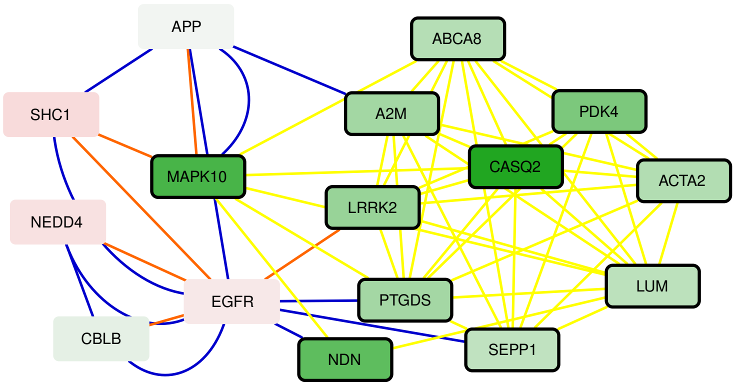

Fig S12.9: Banerji's 2017 dataset: Active module 9

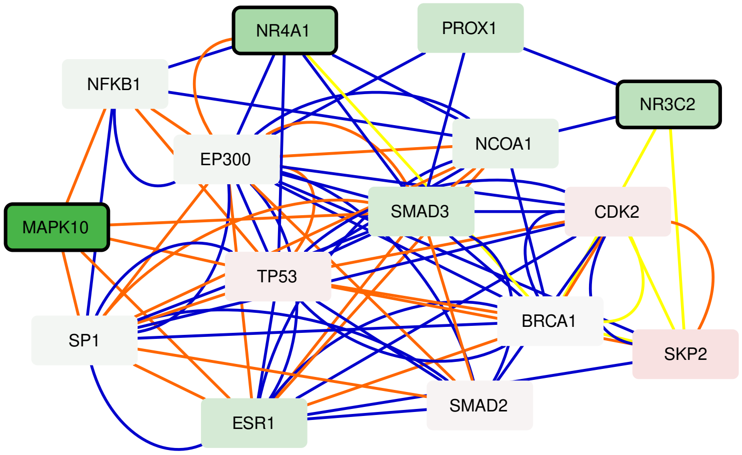

Fig S12.10: Banerji's 2017 dataset: Active module 10

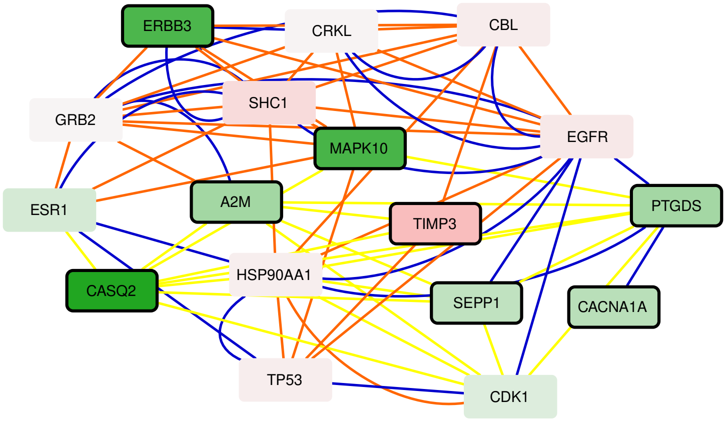

Fig S12.11: Banerji's 2017 dataset: Active module 11

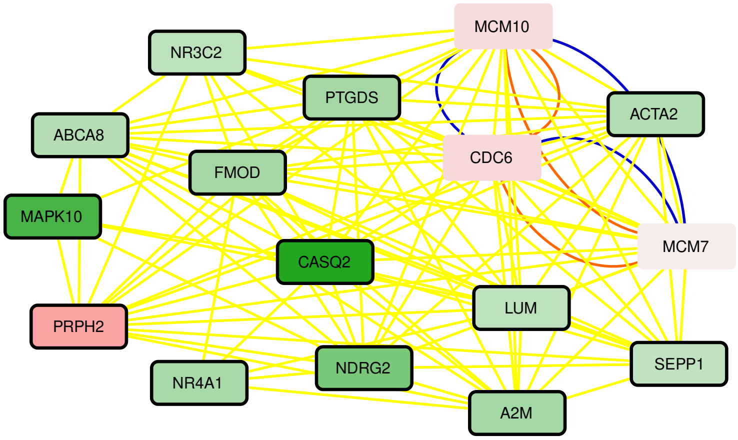

Fig S12.12: Banerji's 2017 dataset: Active module 12

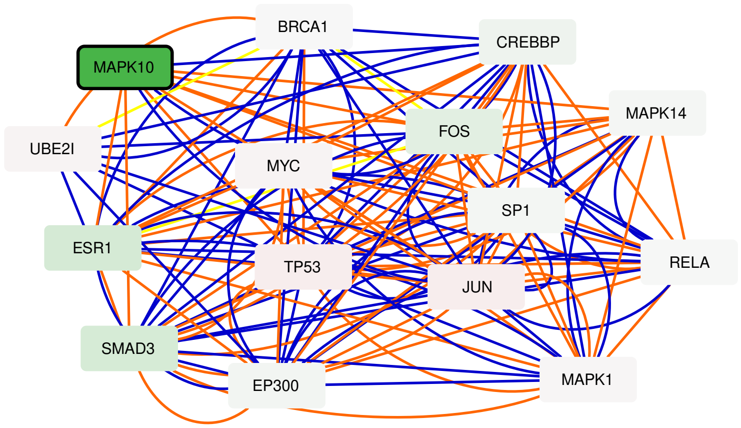

Fig S12.13: Banerji's 2017 dataset: Active module 13

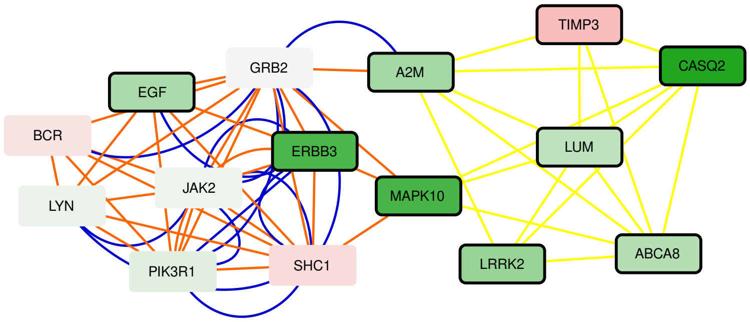

Fig S12.14: Banerji's 2017 dataset: Active module 14

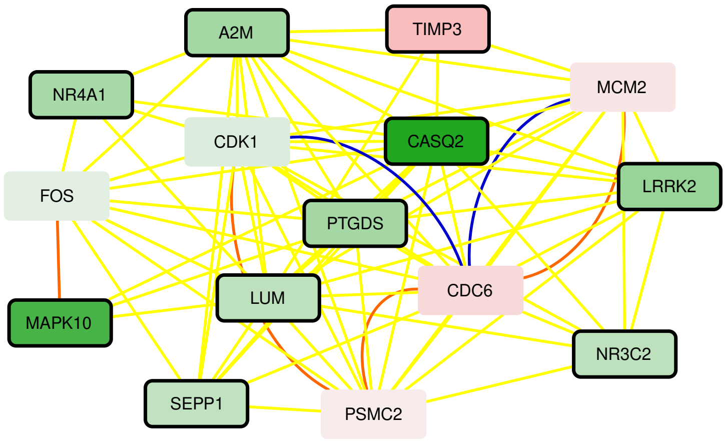

Fig S12.15: Banerji's 2017 dataset: Active module 15

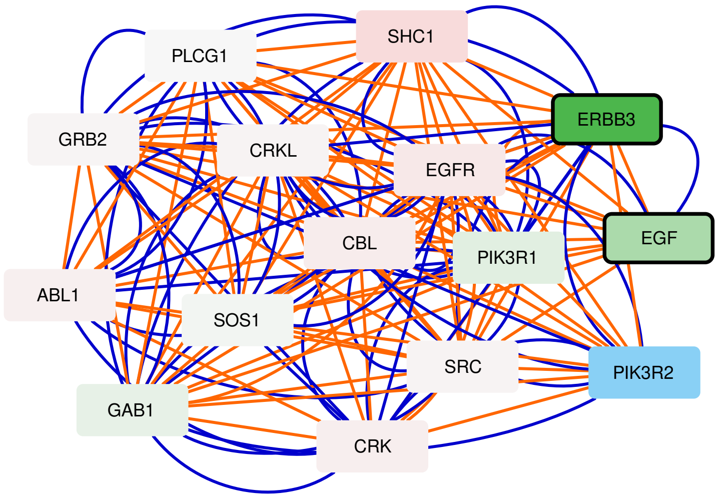

Fig S12.16: Banerji's 2017 dataset: Active module 16

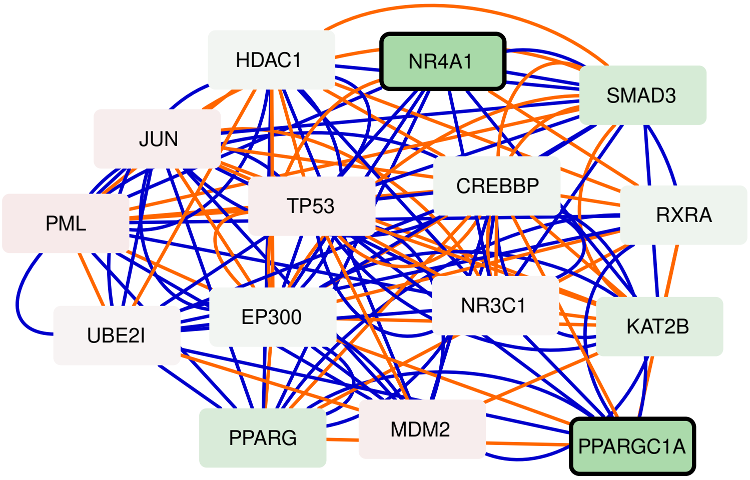

Fig S12.17: Banerji's 2017 dataset: Active module 17

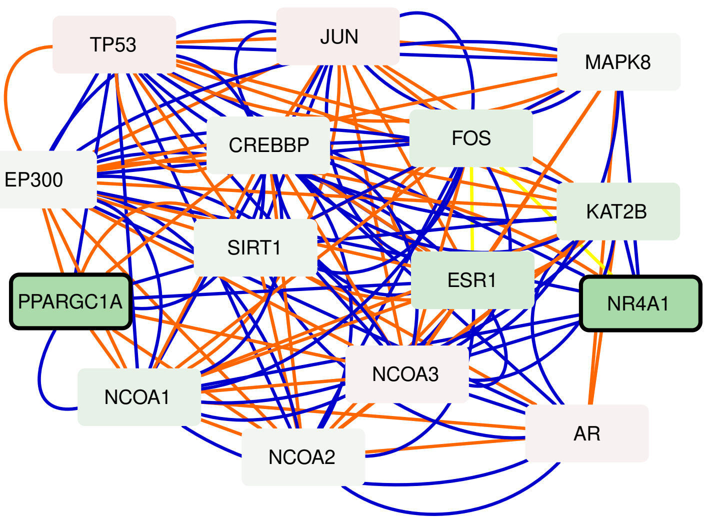

Fig S12.18: Banerji's 2017 dataset: Active module 18

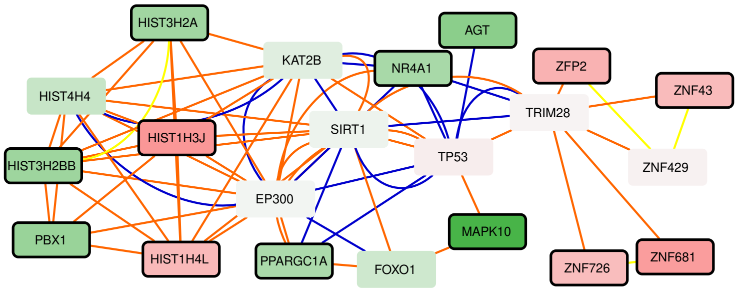

Fig S12.19: Banerji's 2017 dataset: Active module 19

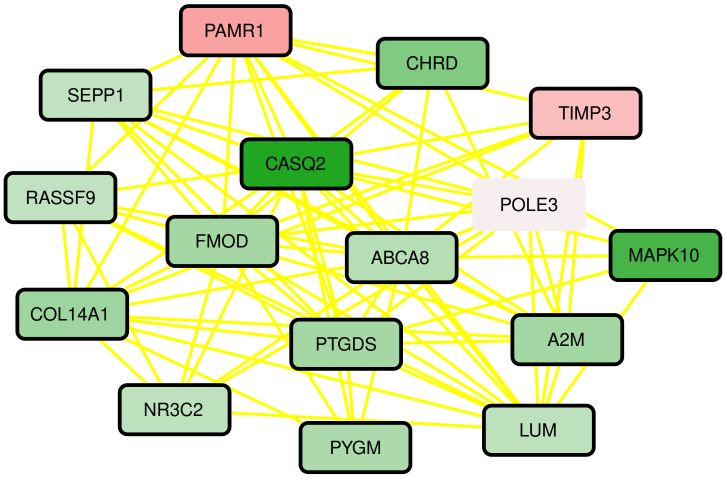

Fig S12.20: Banerji's 2017 dataset: Active module 20

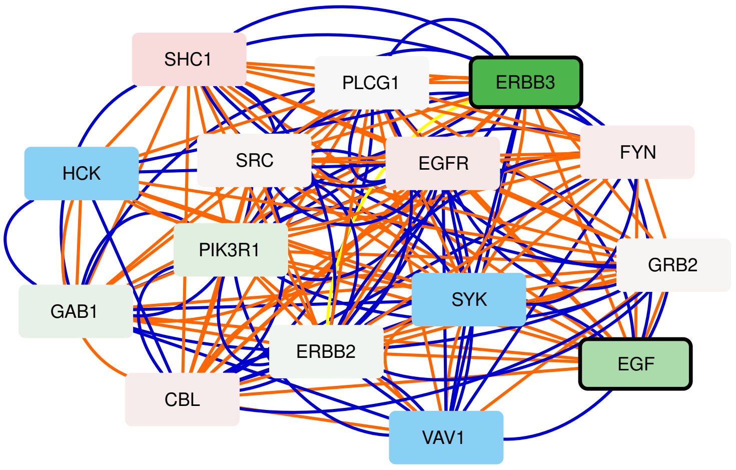

Fig S12.21: Banerji's 2017 dataset: Active module 21

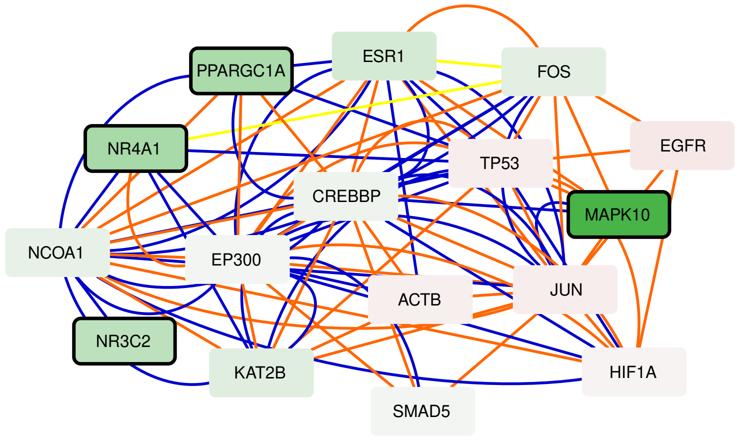

Fig S12.22: Banerji's 2017 dataset: Active module 22

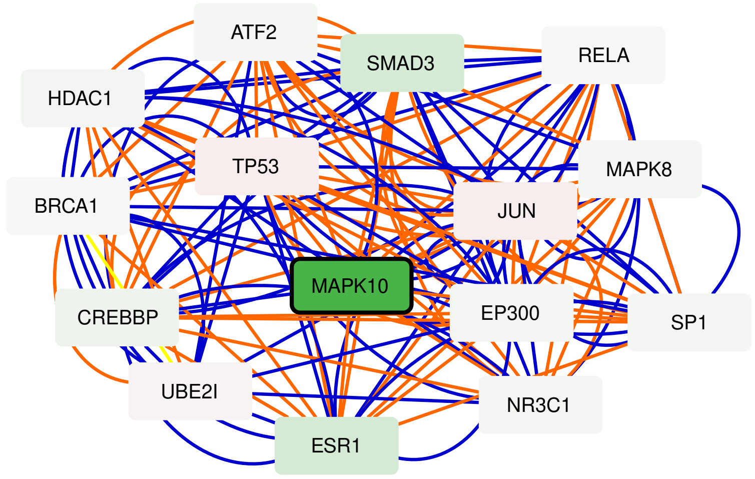

Fig S12.23: Banerji's 2017 dataset: Active module 23

Fig S13

We obtained seventeen active modules obtained by applying MOGAMUN to the Banerji's 2019 dataset [3] (see Table S3 for the list of samples). The color of the nodes represents the fold-change, where green and red nodes correspond to under- and over-expressed genes, respectively. The background node color intensity denotes the strength of the deregulation. Nodes with bold black border correspond to genes significantly differentially expressed (FDR < 0.05 and absolute log_2 fold-change > 1). Blue and white nodes correspond to genes with no associated transcriptomics data and no deregulation, respectively. The color of the edges represents the layer of the multiplex network, where blue, orange, and yellow correspond to PPI, Pathways, and Co-expression, respectively.

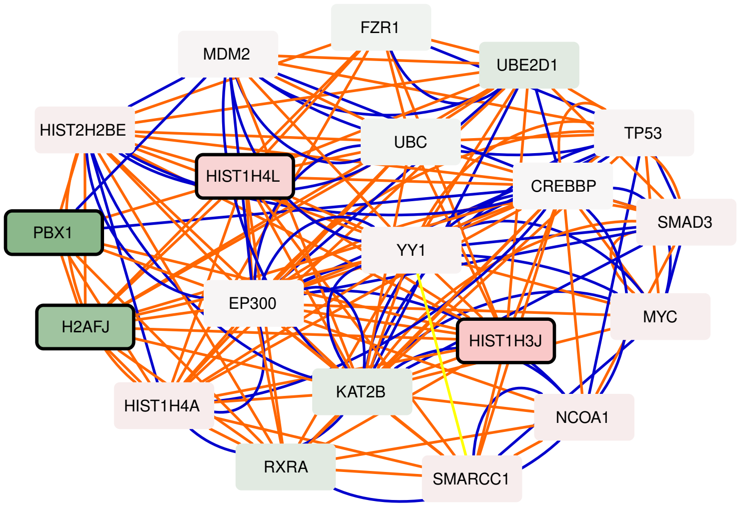

Fig S13.1: Banerji's 2019 dataset: Active module 1

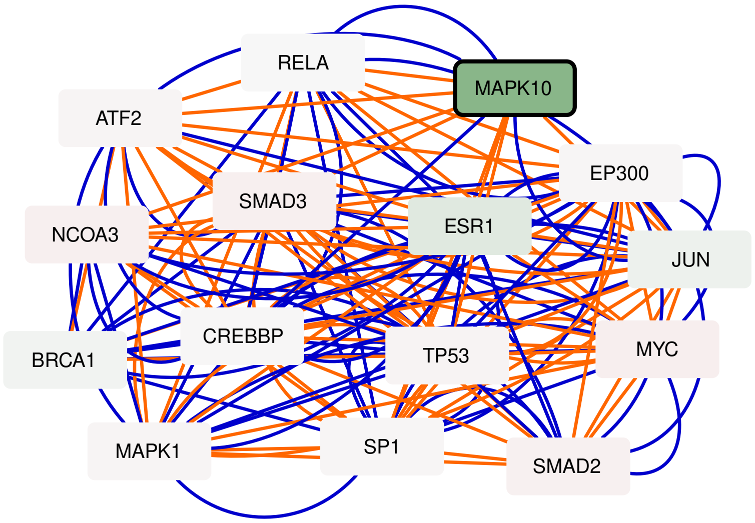

Fig S13.2: Banerji's 2019 dataset: Active module 2

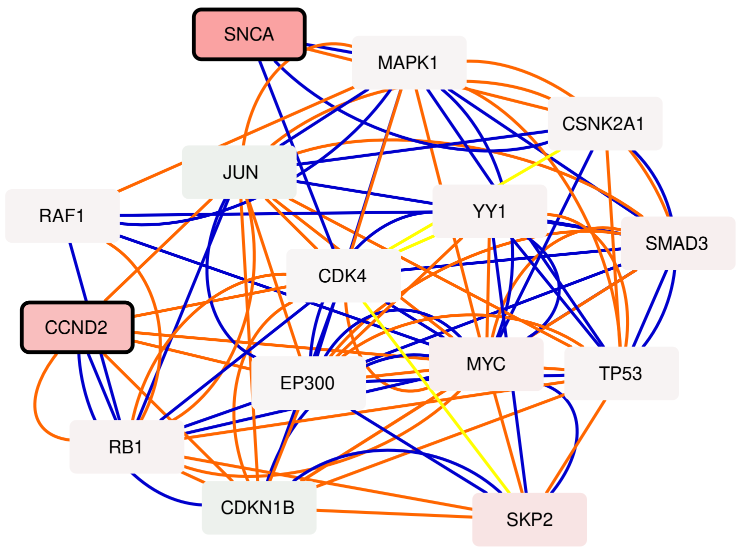

Fig S13.3: Banerji's 2019 dataset: Active module 3

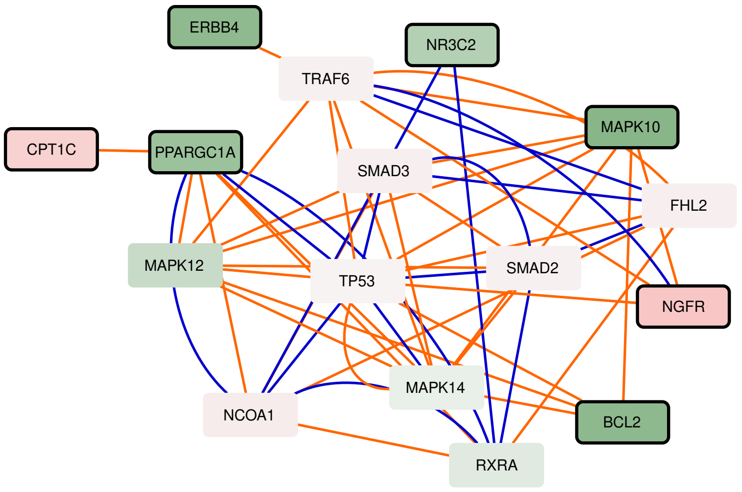

Fig S13.4: Banerji's 2019 dataset: Active module 4

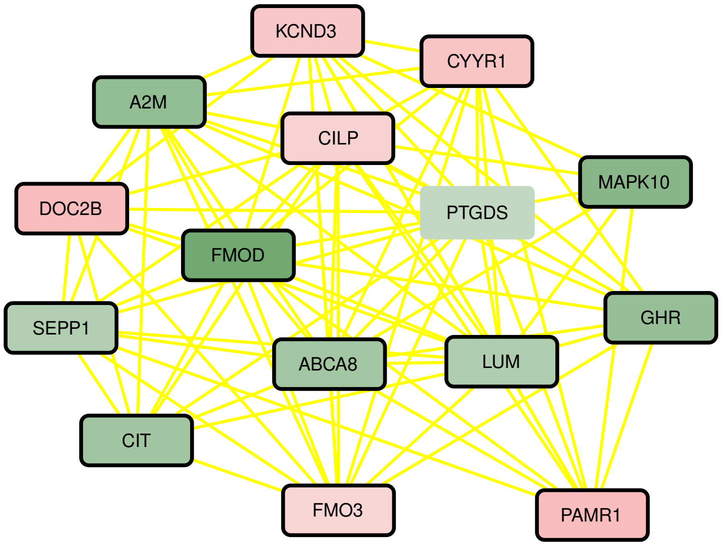

Fig S13.5: Banerji's 2019 dataset: Active module 5

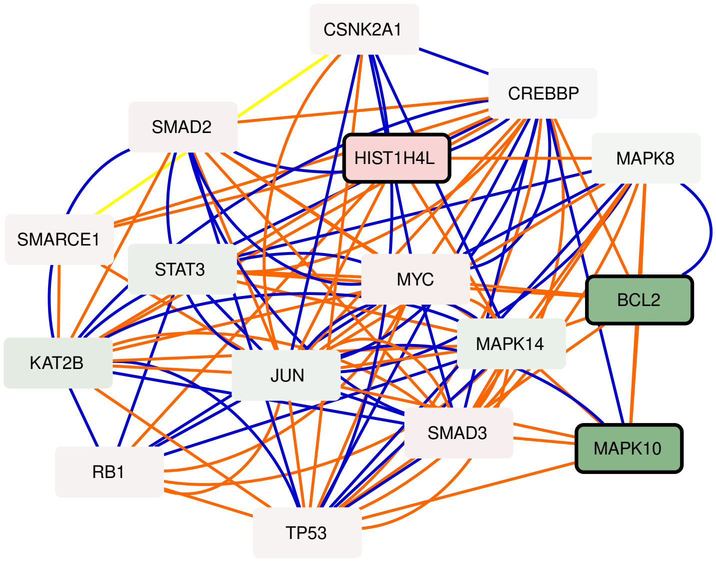

Fig S13.6: Banerji's 2019 dataset: Active module 6

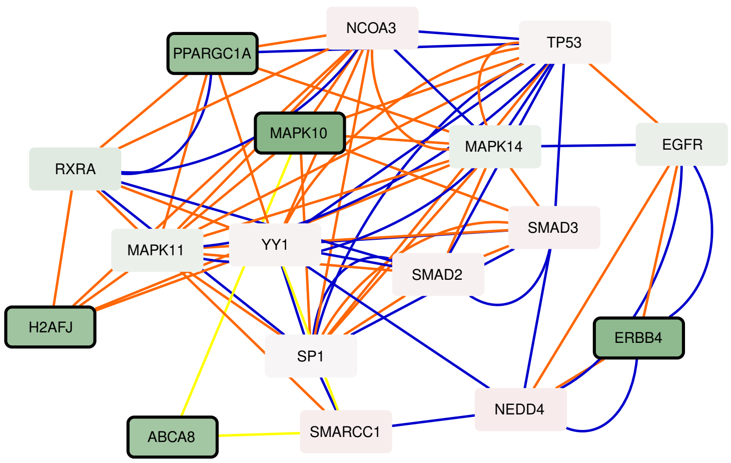

Fig S13.7: Banerji's 2019 dataset: Active module 7

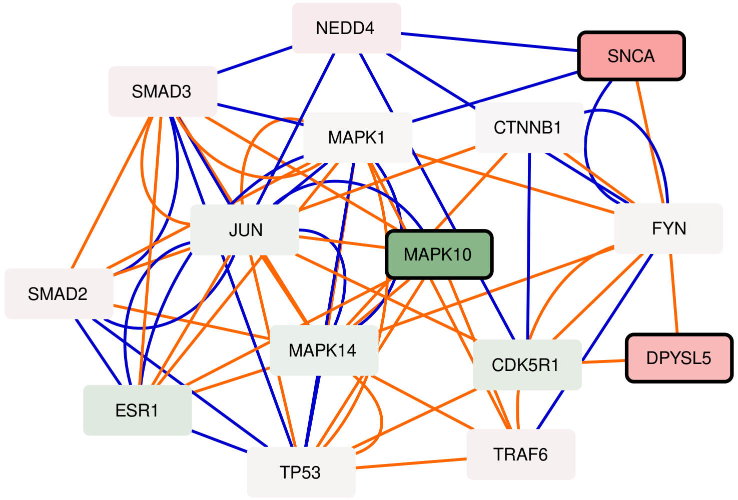

Fig S13.8: Banerji's 2019 dataset: Active module 8

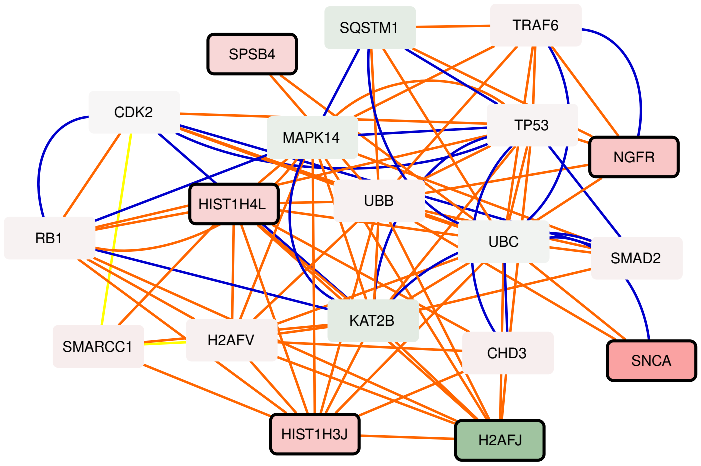

Fig S13.9: Banerji's 2019 dataset: Active module 9

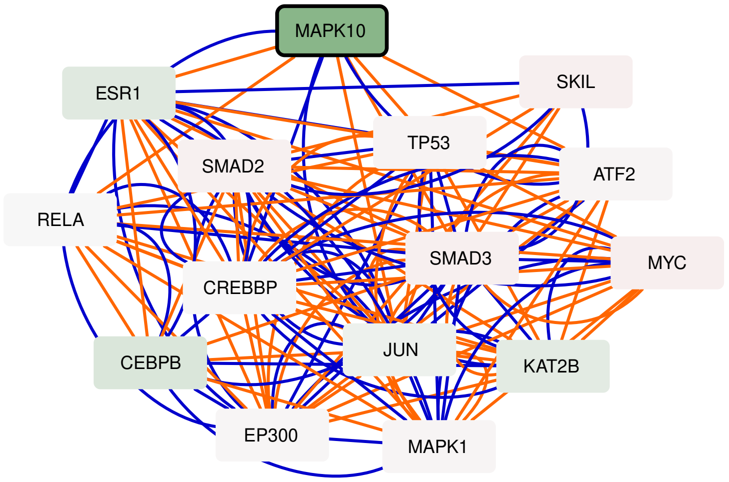

Fig S13.10: Banerji's 2019 dataset: Active module 10

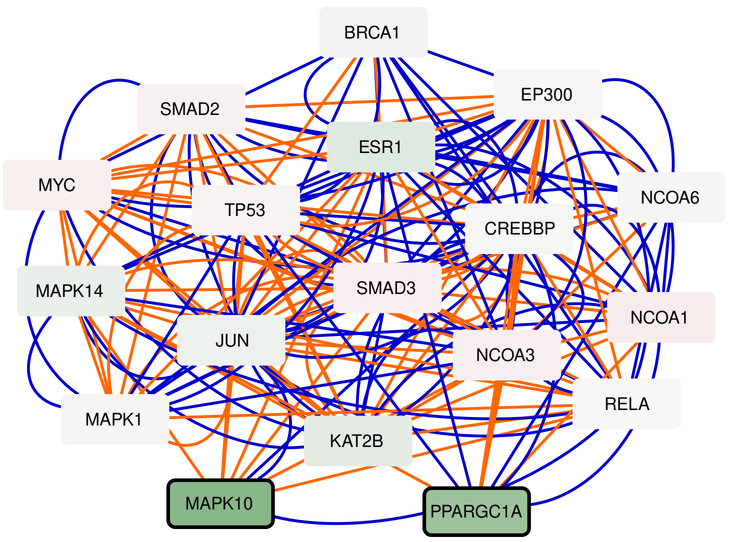

Fig S13.11: Banerji's 2019 dataset: Active module 11

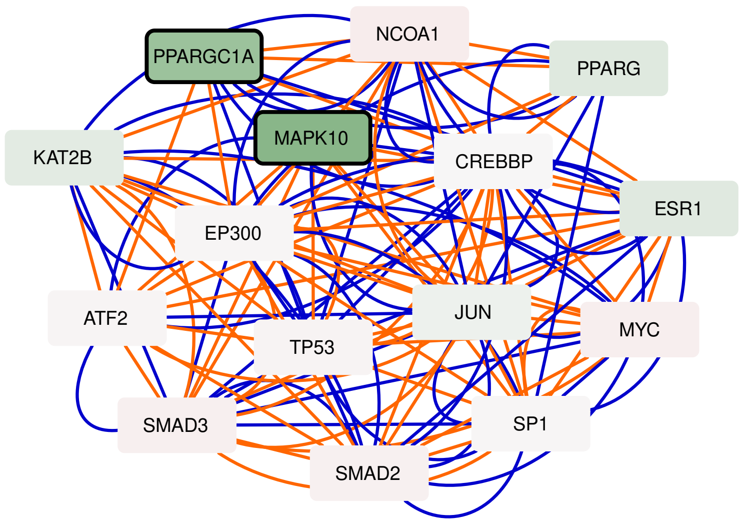

Fig S13.12: Banerji's 2019 dataset: Active module 12

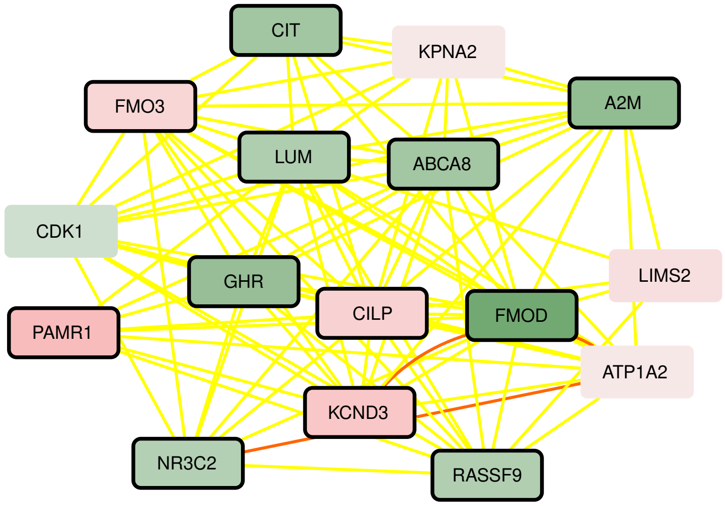

Fig S13.13: Banerji's 2019 dataset: Active module 13

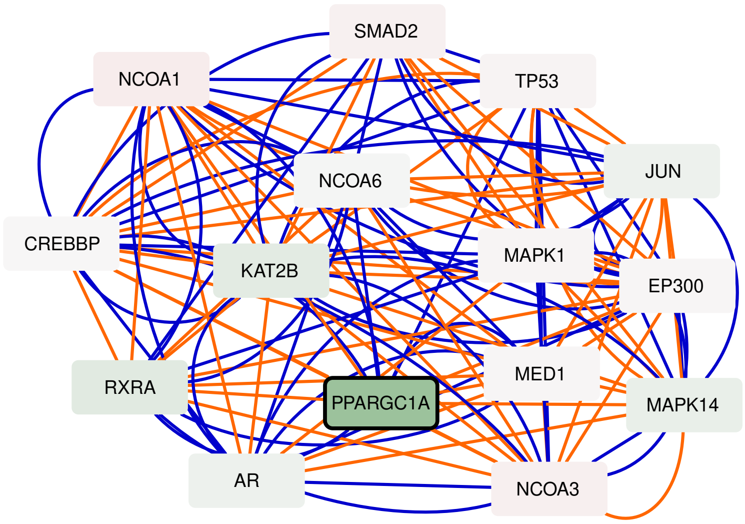

Fig S13.14: Banerji's 2019 dataset: Active module 14

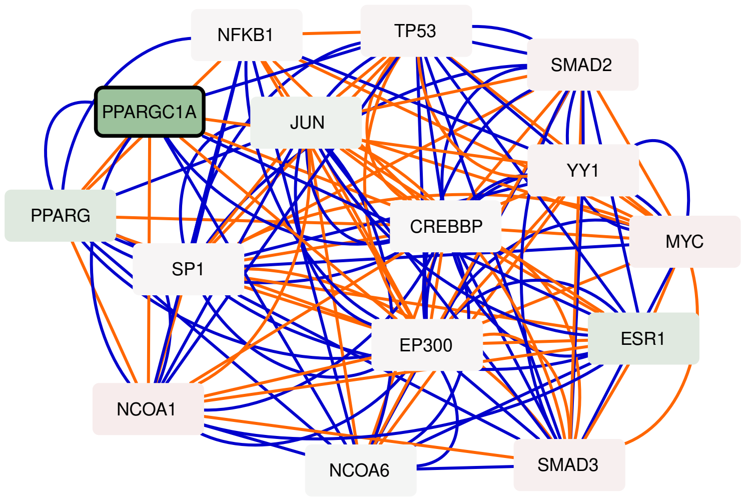

Fig S13.15: Banerji's 2019 dataset: Active module 15

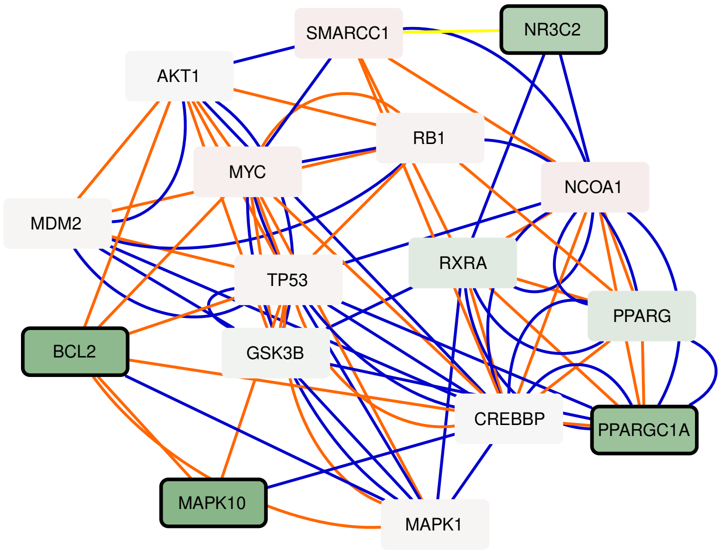

Fig S13.16: Banerji's 2019 dataset: Active module 16

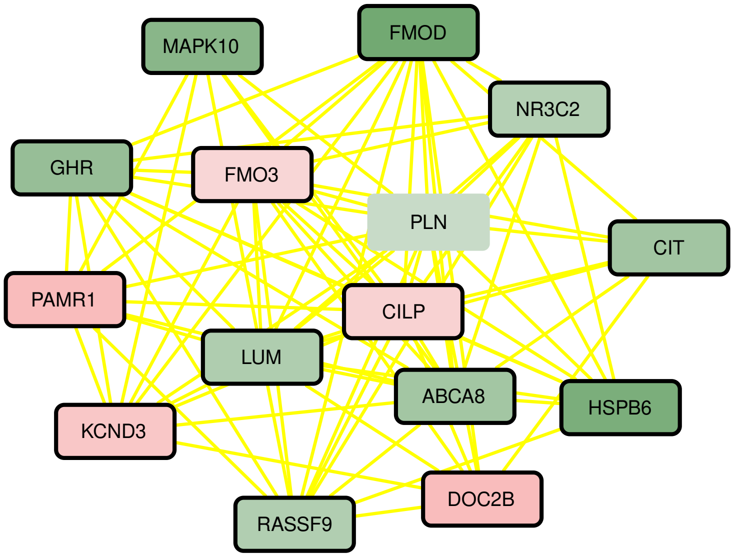

Fig S13.17: Banerji's 2019 dataset: Active module 17

2 Supplementary Tables

**Sample_ID**	**Type**	**Origin**
F1	Patient	Biopsy
F2	Patient	Biopsy
F3	Patient	Biopsy
F4	Patient	Biopsy
F5	Patient	Biopsy
F6	Patient	Biopsy
F7	Patient	Biopsy
F8	Patient	Biopsy
F9	Patient	Biopsy
C1	Control	Biopsy
C2	Control	Biopsy
C3	Control	Biopsy
C4	Control	Biopsy
C5	Control	Biopsy
C6	Control	Biopsy
C7	Control	Biopsy
C8	Control	Biopsy
C9	Control	Biopsy
F4	Patient	Myoblast
F6	Patient	Myoblast
C21	Control	Myoblast
C22	Control	Myoblast
F4	Patient	Myotube
F6	Patient	Myotube
C20	Control	Myotube
C21	Control	Myotube
C22	Control	Myotube

Table S1: Samples from Yao's datasets [1]. Downloaded from `https://www.ncbi.nlm.nih.gov/geo/query/acc.cgi?acc=GSE56787`

ID	Type	Batch
54_12_r1	Patient	1
54_12_r2	Patient	1
54_12_r3	Patient	1
54_6_r1	Control	1
54_6_r2	Control	1
54_6_r3	Control	1
54_2_r1	Patient	2
54_2_r2	Patient	2
54_2_r3	Patient	2
54_A5_r1	Patient	2
54_A5_r2	Patient	2
54_A5_r3	Patient	2
54_A10_r1	Control	2
54_A10_r2	Control	2
54_A10_r3	Control	2
12ABic_r1	Patient	3
12ABic_r2	Patient	3
12ABic_r3	Patient	3
16ABic_r1	Patient	3
16ABic_r2	Patient	3
16ABic_r3	Patient	3
12UBic_r1	Control	3
12UBic_r2	Control	3
12UBic_r3	Control	3
16UBic_r1	Control	3
16UBic_r2	Control	3
16UBic_r3	Control	3

Table S2: Samples from Banerji's 2017 dataset [2]. Downloaded from `https://www.ncbi.nlm.nih.gov/geo/query/acc.cgi?acc=GSE102812`

ID	Type	Batch
54_12_T8_r1	Patient	1
54_12_T8_r2	Patient	1
54_12_T8_r3	Patient	1
54_6_T8_r1	Control	1
54_6_T8_r2	Control	1
54_6_T8_r3	Control	1
54_2_T8_r1	Patient	2
54_2_T8_r2	Patient	2
54_2_T8_r3	Patient	2
54_A5_T8_r1	Patient	2
54_A5_T8_r2	Patient	2
54_A5_T8_r3	Patient	2
54_A10_T8_r1	Control	2
54_A10_T8_r2	Control	2
54_A10_T8_r3	Control	2
12A_T8_r1	Patient	3
12A_T8_r2	Patient	3
12A_T8_r3	Patient	3
16A_T8_r1	Patient	3
16A_T8_r2	Patient	3
16A_T8_r3	Patient	3
12U_T8_r1	Control	3
12U_T8_r2	Control	3
12U_T8_r3	Control	3
16U_T8_r1	Control	3
16U_T8_r2	Control	3
16U_T8_r3	Control	3

Table S3: Samples from Banerji's 2019 dataset [3]. Downloaded from `https://www.ncbi.nlm.nih.gov/geo/query/acc.cgi?acc=GSE123468`

3 Non-dominated Sorting Genetic Algorithm II (NSGA-II)

Non-dominated Sorting Genetic Algorithm II (NSGA-II) [4] is a well-known and widely tested multi-objective evolutionary algorithm. It is based on a genetic algorithm, and specifically designed to optimize two or more objectives simultaneously (it is particularly competitive for two and three objective functions). In MOGAMUN, we used NSGA-II to search for solutions providing a trade-off between two objective functions.

Non-domination sorting

NSGA-II sorts the population P by using a non-domination criterion, dividing the population in Pareto sets, in order to generate different Pareto fronts $\mathcal{F}$, as shown in Algorithm S1 [4]. From the current population P, non-dominated solutions are labeled with rank 1, i.e., they belong to the first Pareto front ($\mathcal{F}_1$), and are therefore the best solutions.

Crowding distance

An important concept in NSGA-II is the crowding distance, which allows evaluating the density of solutions around a given point in the objective space. In other words, the crowding distance evaluates the proximity of a solution to its neighbors from the same Pareto front $\mathcal{F}_j$. In Supplementary Fig S14, the crowding distance of the point i is an estimate of the size of the cuboid formed by its neighbors $i-1$ and $i+1$, considering the two objective functions f_1 and f_2.

Algorithm S1 Fast non-dominated sorting [4]

procedure FAST-NON-DOMINATED-SORT(P)
    for each $p \in P$ **do**
        $S_p = \emptyset$
        $n_p = 0$
        for each $q \in P$ **do**
            if p dominates q **then**
                $S_p = S_p \bigcup \{q\}$ ▷ Add q to the set of solutions dominated by p
            else
                if q dominates p **then**
                    $n_p = n_p + 1$ ▷ Increment the domination counter of p
                end if
            end if
        end for
        if $n_p == 0$ **then** ▷ p belongs to the first Pareto front
            $p_{rank} = 1$
            $\mathcal{F}_1 = \mathcal{F}_1 \bigcup \{p\}$
        end if
    end for
    $i = 1$ ▷ Initialize the front counter
    while $\mathcal{F}_i \neq \emptyset$ **do**
        $\mathcal{Q} = \emptyset$
        for each $p \in \mathcal{F}_i$ **do**
            for each $q \in S_p$ **do**
                $n_q = n_q - 1$ ▷ Decrease the domination counter of q
                if $n_q == 0$ **then** ▷ q belongs to the next front
                    $q_{rank} = i + 1$
                    $\mathcal{Q} = \mathcal{Q} \bigcup \{q\}$
                end if
            end for
        end for
        $i = i + 1$
        $\mathcal{F}_i = \mathcal{Q}$
    end while
end procedure

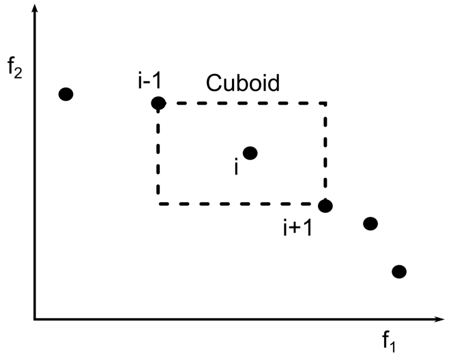

Fig S14: Crowding distance concept. Image adapted from [4]

The crowding distance calculation is presented in Algorithm S2, where $\mathcal{I}$ is the set of solutions in a given Pareto front, and $\mathcal{I}[i].m$ refers to the value of the m-th objective function of the i-th individual in set $\mathcal{I}$.

Algorithm S2 Crowding distance assignment [4]

procedure CROWDING-DISTANCE-ASSIGNMENT($\mathcal{I}$)
  l = number of solutions in $\mathcal{I}$
  for each $i \in \{1, ..., l\}$ **do**
    $\mathcal{I}[i]_{distance} = 0$
  end for
  for each objective m in list of objectives **do**
    $\mathcal{I}$ = sort $\mathcal{I}$ with respect to m
    $\mathcal{I}[1]_{distance} = \mathcal{I}[l]_{distance} = \infty$ to always select boundary points
    for $i = 2$ to $(l-1)$ **do**   for all other points
      $\mathcal{I}[i]_{distance} = \mathcal{I}[i]_{distance} + (\mathcal{I}[i+1].m - \mathcal{I}[i-1].m)/(f_m^{max} - f_m^{min})$
    end for
  end for
end procedure

Crowded comparison operator

Once each solution i has a non-domination rank i_{rank} (corresponding to the Pareto front the solution belongs to), and a crowding distance $i_{distance}$, the crowded comparison operator, denoted as $\geq_n$, helps determine which one out of two solutions is better. More specifically, $i \geq_n j$ if $(i_{rank} < j_{rank})$ or $((i_{rank} == j_{rank})$ and $(i_{distance} > j_{distance}))$. In other words, if two solutions have different ranks, we choose the one with the better (i.e., lower) rank, otherwise, we choose the one with the bigger crowding distance (i.e., the one with the largest cuboid).

Main loop

In NSGA-II, the initial population of solutions P_0 of size N is generated at random and sorted by non-domination. The fitness of a solution is the number of Pareto front in which it was ranked, where 1 is the best ranking. Using P_0 as parents, a new population Q_0 of size N is generated by tournament selection of size two, crossover and mutation operators. The new population P_{t+1} for a particular generation t is selected with elitism, from the populations P_t and Q_t, as shown in Algorithm S3.

The evolution continues using P_{t+1} as parents to generate a new population of solutions, and the process ends when the stopping condition is met.

Algorithm S3 Elitist selection of a new population

procedure ELITIST-SELECTION(P_t, Q_t)
  $R_t = P_t \bigcup Q_t$
  $\mathcal{F}$ = fast-non-dominated-sort(R_t)
  $P_{t+1} = \emptyset$
  $i = 1$
  while $|P_{t+1}| < N$ **do**
    crowding-distance-assignment($\mathcal{F}_i$)
    $P_{t+1} = P_{t+1} \bigcup \mathcal{F}_i$
    $i = i + 1$
  end while
  sort P_{t+1} in descending order using $\geq_n$
  P_{t+1} = the first N elements of P_{t+1}
end procedure

4 MOGAMUN Genetic Algorithm parameter tuning

We applied MOGAMUN with different combinations of parameters to the Yao's dataset, myotubes [1] and a 3-layer multiplex network. The multiplex network is made of a protein-protein interaction network, a pathway network, and a correlation of expression network (see Materials and Methods in the main manuscript). We used the aforementioned RNA-seq expression data as it is the FSHD1 dataset with the highest number of significant DEGs. We ran MOGAMUN, testing all combinations of crossover rates from 0.7 to 1, and mutation rates from 0.1 to 0.3, both with increments of 0.1. These values correspond to common values and combination of values used for genetic algorithms in problems related to our framework [5–7].

Supplementary Fig S15 shows the converge plots of the two objective functions. At each generation, we show the mean of the highest values of the average nodes score (A) and density (B) of the 30 runs for the different sets of parameters. We can observe that the algorithm converges for all combinations of parameters, even if the rate of convergence can be variable. Supplementary Fig S16 shows the average nodes score (A) and density (B) values, and the corresponding overlapping nodes (C) of the active modules obtained in the *accumulated Pareto fronts* of the 30 runs for the different combinations of parameters. We can observe that the achieved values of the two objective functions are very similar in all scenarios. Such behavior suggests that MOGAMUN is not significantly sensitive to its input parameters, which is confirmed by the high overlap between active modules from the different *accumulated Pareto fronts*.

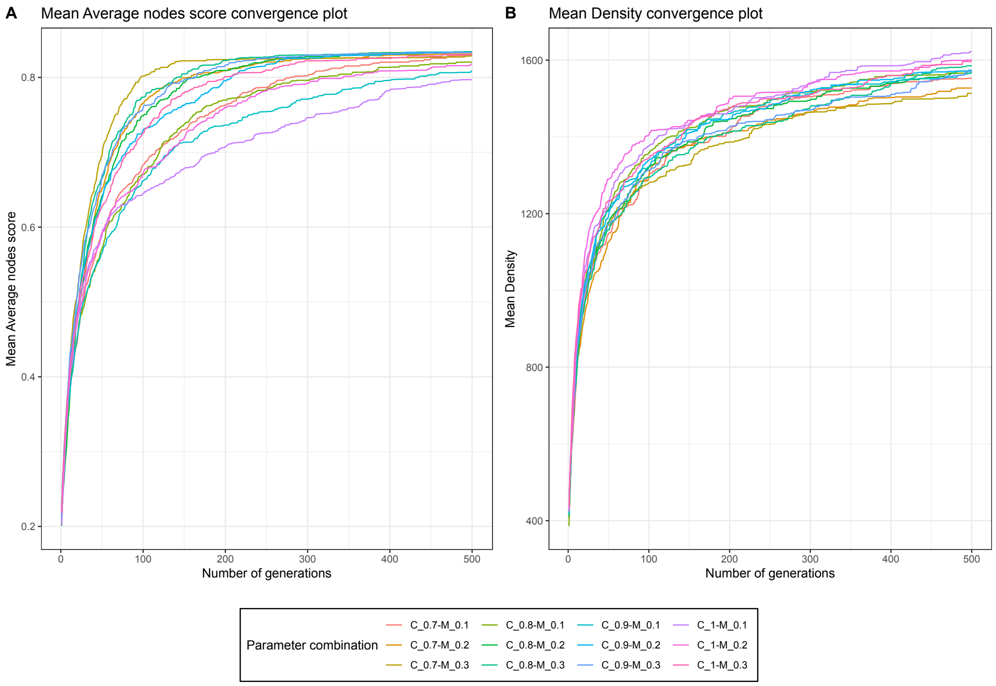

Fig S15: Convergence plots of the average nodes score (A) and density (B). At each generation, the best values for the average nodes score and density of the 30 runs are averaged and plotted

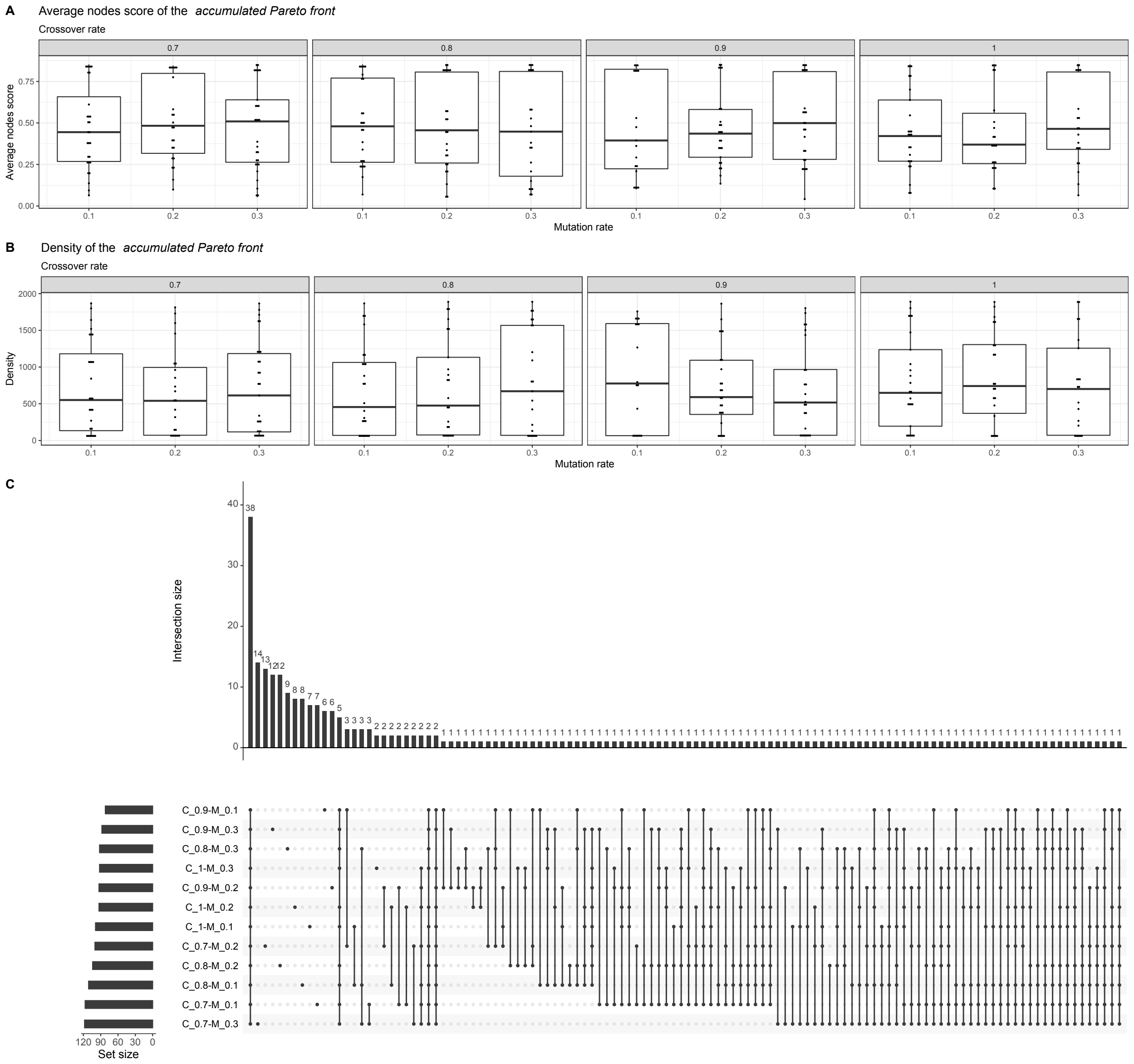

Fig S16: Average nodes score (A), density (B), and overlapping nodes (C) of the active modules obtained in the *accumulated Pareto fronts* of 30 runs of MOGAMUN with different combinations of parameters

5 Application to Facio-Scapulo-Humeral muscular Dystrophy type 1 (FSHD1)

We applied the different active module identification approaches (COSINE, jActiveModules, PinnacleZ and MOGAMUN) to the three RNA-seq datasets of FSHD1 (biopsies, myoblasts and myotubes) obtained from Yao's publication [1], downloaded from `https://www.ncbi.nlm.nih.gov/geo/query/acc.cgi?acc=GSE56787` (see Table S1). We used biological interactions from a multiplex network composed of three layers (protein-protein interactions, pathways, and correlation of expression). We first aggregated the three layers of the multiplex network into a single-layer network because COSINE, jActiveModules, and PinnacleZ can only handle a single monoplex network as input. We removed duplicated edges from the aggregated network, so that, if an edge is present in the three layers of the multiplex, a single edge is representing it in

the aggregated single-layer network. We decided to do so because none of the tested methods (MOGAMUN included) could handle weighted networks as an input. For the sake of comparison, we additionally applied MOGAMUN to the aggregated network, using the same parameters as the ones used for MOGAMUN on the multiplex network in the main manuscript. We hence have two versions of MOGAMUN, MOGAMUNaggr (applied on the aggregated network) and MOGAMUNmulti (applied on the multiplex network). Note that MOGAMUNmulti corresponds to the settings and results presented in detail in the main manuscript (sections 2.3 and 3.2, respectively) and for which we provide biological interpretation.

We ran each algorithm 30 times on each FSHD1 RNA-seq expression dataset. We modified some of the parameters that were used for COSINE and jActiveModules in the benchmark experiments we present in the main manuscript, as follows:

COSINE. We reduced the number of generations from 5 000 to 500 because i) it's the number of generations we use for MOGAMUN, and ii) 5 000 generations would have lasted ∼33 hours per run with such a big network, and we needed to run it 90 times to analyse the different expression datasets.

jActiveModules. We increased the number of active modules to be retrieved from 1 to 5, which is the default value in [8]. The running time for each run (with quenching) was about 10-15 minutes.

For PinnacleZ, we used the same parameters as in the two benchmark experiments from the main manuscript, which are the same reported in [9] (i.e., distance from the seed = 2 nodes, minimal mutual information score improvement threshold = 0.05), and we set the maximum size per subnetwork = 50 (the same size that we allowed for MOGAMUN).

We then compared the results obtained by the five approaches (jActiveModules, COSINE, PinnacleZ, MOGAMUN on multiplex networks -MOGAMUNmulti-, and MOGAMUN on the aggregated network -MOGAMUNaggr-). Please note that *active modules* or *subnetworks* are used to refer to the outputs of the different approaches indifferently.

5.1 Distribution of size, density and average nodes score obtained by the different methods

The results obtained on the three expression datasets are compared based on the size, average nodes score, and density of the obtained modules. Note that the density is computed both on the aggregated network (aggregated density) using the classical density formula (Equation 4 in section 2.1.1 of the main manuscript), and on the multiplex network (multiplex density), using the normalized density presented in the manuscript (Equation 3 in section 2.1.1).

5.1.1 Analyses of Yao's dataset, biopsies

On Yao's dataset, biopsies (Supplementary Figs S17, S18 and S19), PinnacleZ retrieved many small modules with less than 15 nodes, as observed in the benchmark experiments (see section 3.1 of the main manuscript). COSINE outputs very large modules, each with more than 500 nodes. The size distribution of the active modules retrieved by jActiveModules is quite peculiar, with more than one hundred very small modules (corresponding to modules composed of one node) and a few large modules. In each run, jActiveModules typically identified one large module (the aggregated node score is biased towards the retrieval of large modules, as demonstrated in [10]) and four singletons (i.e., modules composed a single node). The two versions of MOGAMUN retrieved modules of sizes between 15 and 19 nodes, but overall MOGAMUNaggr identified fewer active modules than MOGAMUNmulti. The average nodes score of the subnetworks retrieved by jActiveModules is slightly higher than MOGAMUN's. Yet, such score is achieved because modules composed of a single node are usually significant DEGs. PinnacleZ seems to retrieve dense subnetworks but, again, these subnetworks are numerous, and PinnacleZ overall does not identify modules composed of at least 15 nodes. The two other methods (jActiveModules and COSINE), do not identify dense modules; this is expected as these methods do not optimize for the density of the subnetworks to be retrieved.

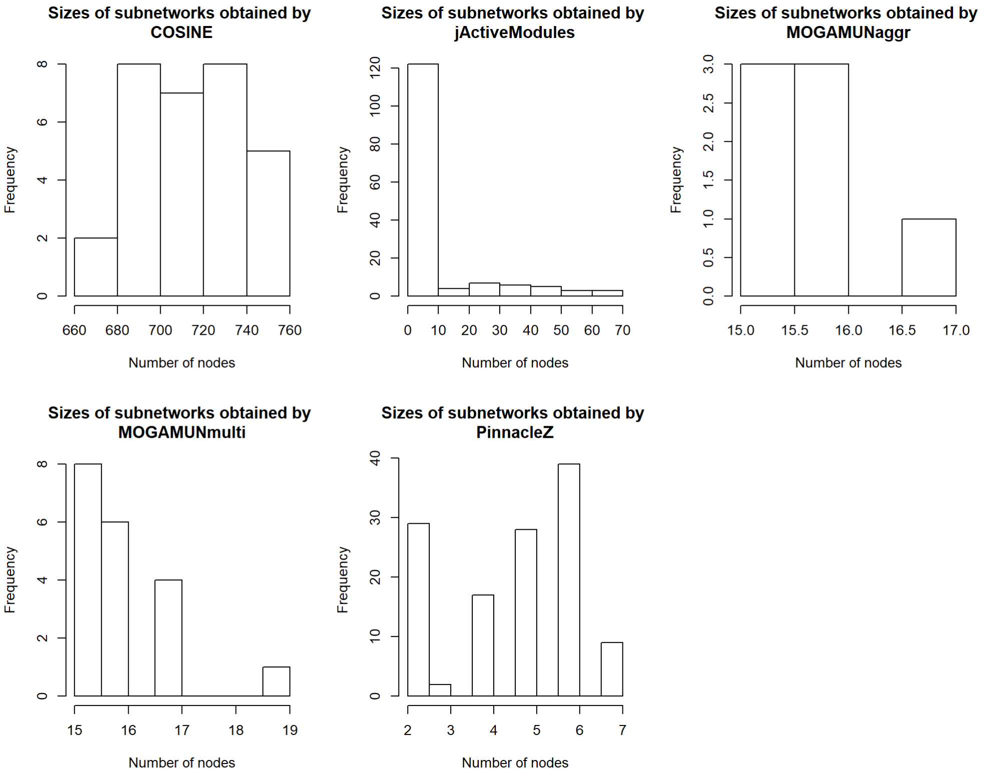

Fig S17: Sizes of the subnetworks identified by the five approaches in Yao's dataset, biopsies

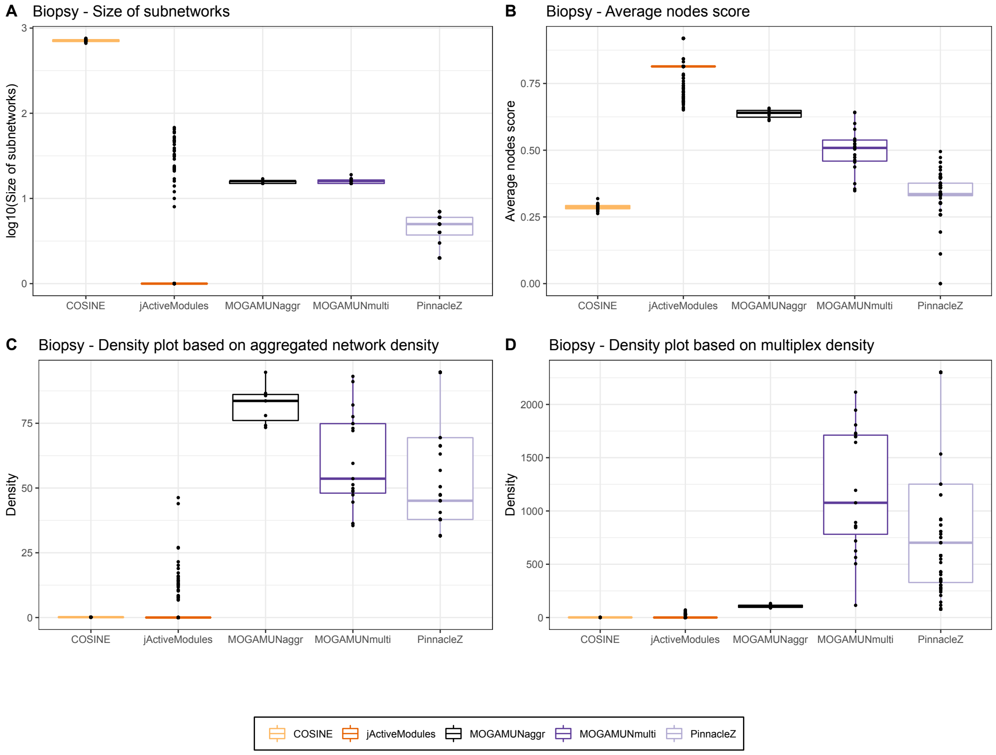

Fig S18: Size, average nodes score and density of the subnetworks obtained by the different methods using Yao's dataset, biopsies. The sizes of the subnetworks are represented on a log scale. The density is computed either on the aggregated network, corresponding to the union of the three biological networks used in this study, or using the multiplex-normalized density, proposed in the main manuscript

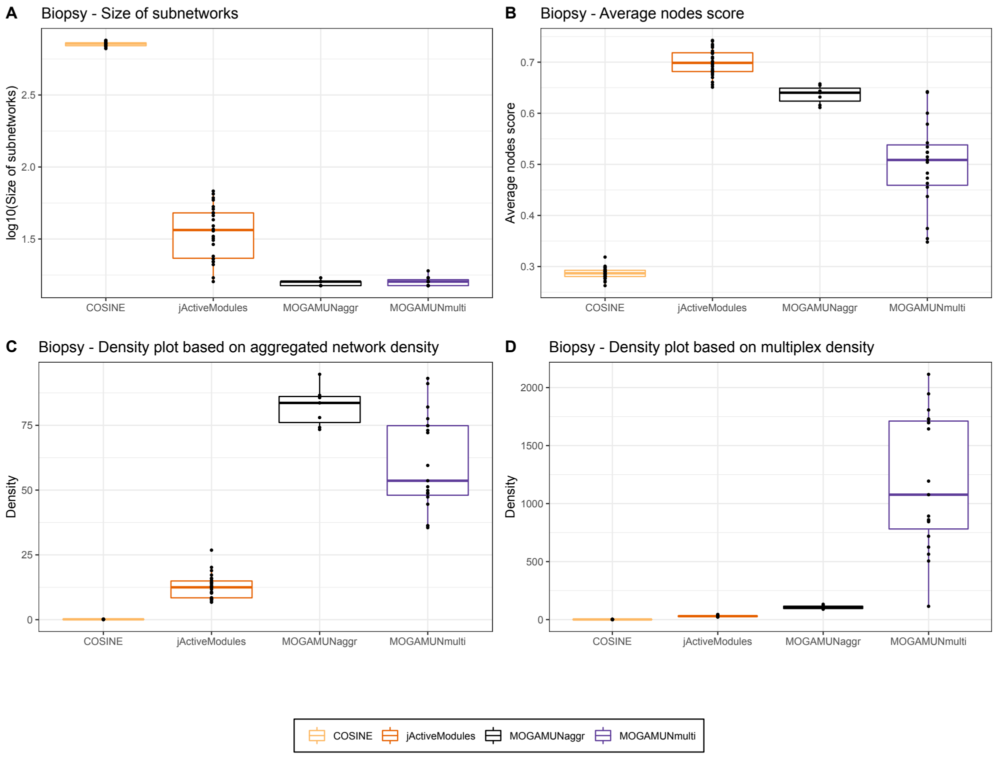

Fig S19: Size, average nodes score and density of the subnetworks obtained by the different methods using Yao's dataset, biopsies, selecting only the subnetworks containing at least 15 nodes. The sizes of the subnetworks are represented on a log scale. The density is computed either on the aggregated network, corresponding to the union of the three biological networks used in this study, or using the multiplex-normalized density, proposed in the main manuscript

5.1.2 Analyses of Yao's dataset, myotubes

A similar pattern to the biopsies dataset is observed for the application of the methods on Yao's dataset, myotubes (Supplementary Figs S20, S21 and S22), except that in this case, the average nodes score of the active modules retrieved by MOGAMUN is comparable to those retrieved by jActiveModules.

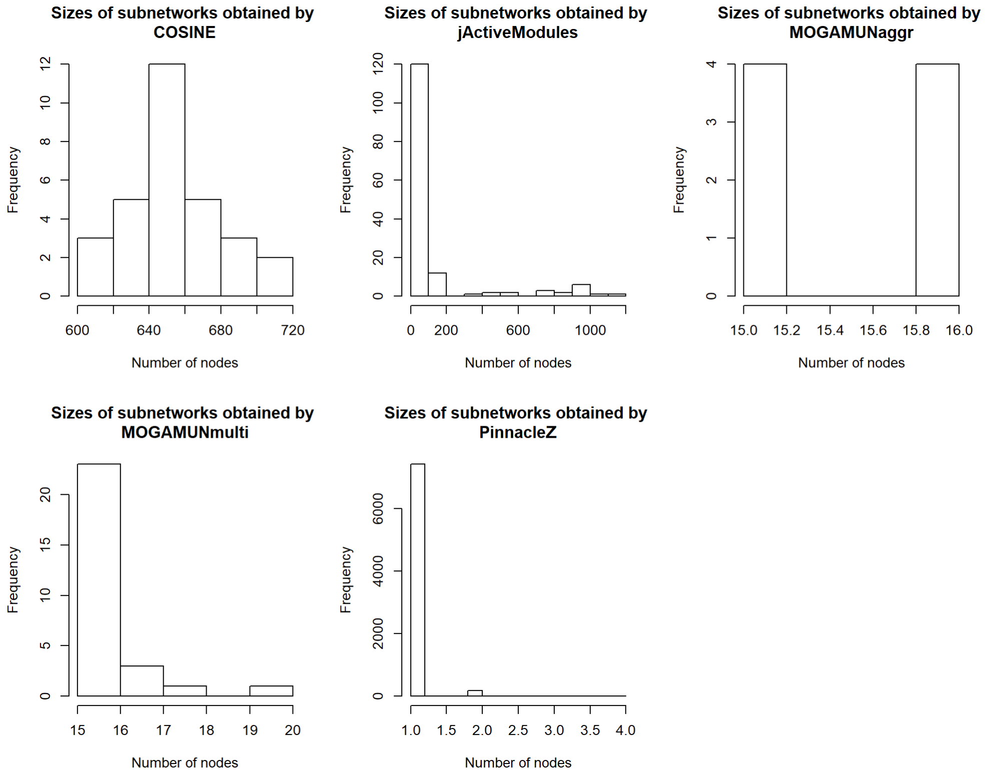

Fig S20: Sizes of the subnetworks identified by the five approaches on Yao's dataset, myotubes

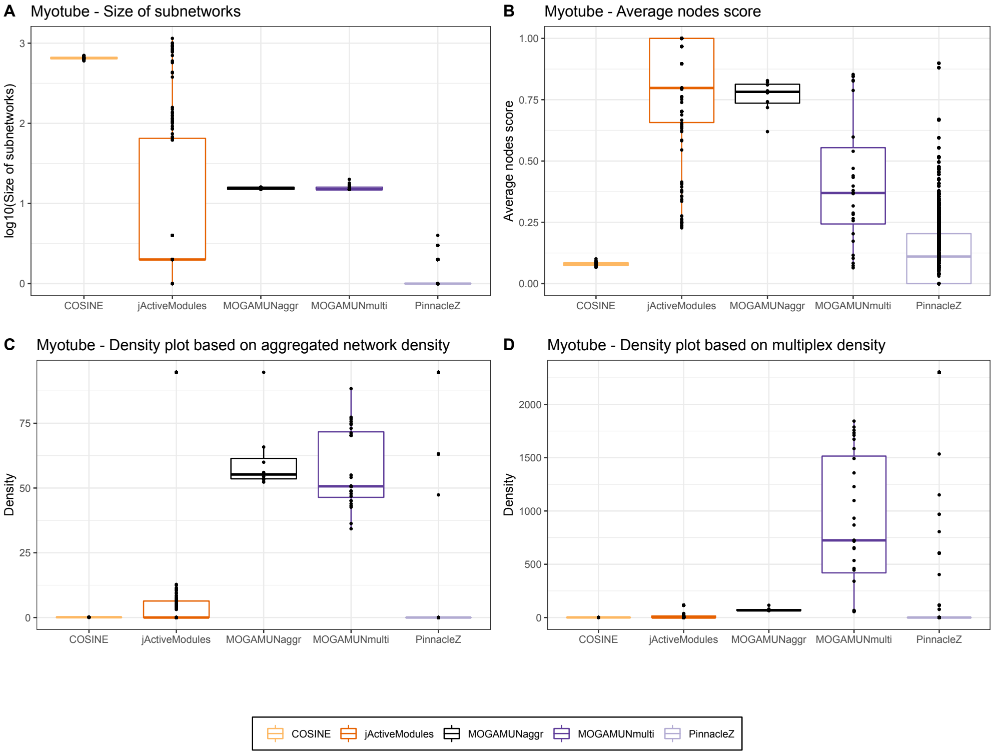

Fig S21: Size, average nodes score and density of the subnetworks obtained by the different methods using Yao's dataset, myotubes. The sizes of the subnetworks are represented on a log scale. The density is computed either on the aggregated network, corresponding to the union of the three biological networks used in this study, or using the multiplex-normalized density, proposed in the main manuscript

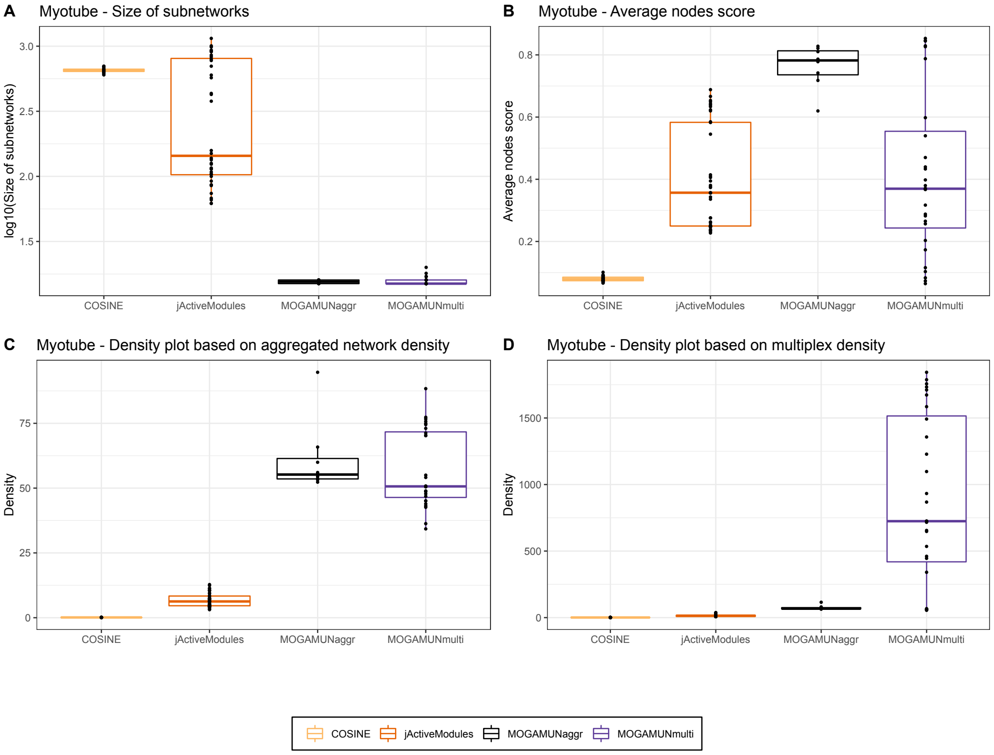

Fig S22: Size, average nodes score and density of the modules obtained by the different methods using Yao's dataset, myotubes, selecting only the subnetworks containing at least 15 nodes. The sizes of the subnetworks are represented on a log scale. The density is computed either on the aggregated network, corresponding to the union of the three biological networks used in this study, or using the multiplex-normalized density, proposed in the main manuscript

5.1.3 Analyses of Yao's dataset, myoblasts

Finally, we observed that the identification of active modules from Yao's dataset, myoblasts might be a more complex task (Supplementary Figs S23, S24). Indeed, in this case, only MOGAMUN and COSINE retrieved modules containing at least 15 nodes (Supplementary Fig S25). The global behavior of the different approaches is overall similar to the one observed for the other two Yao's datasets (biopsies and myotubes).

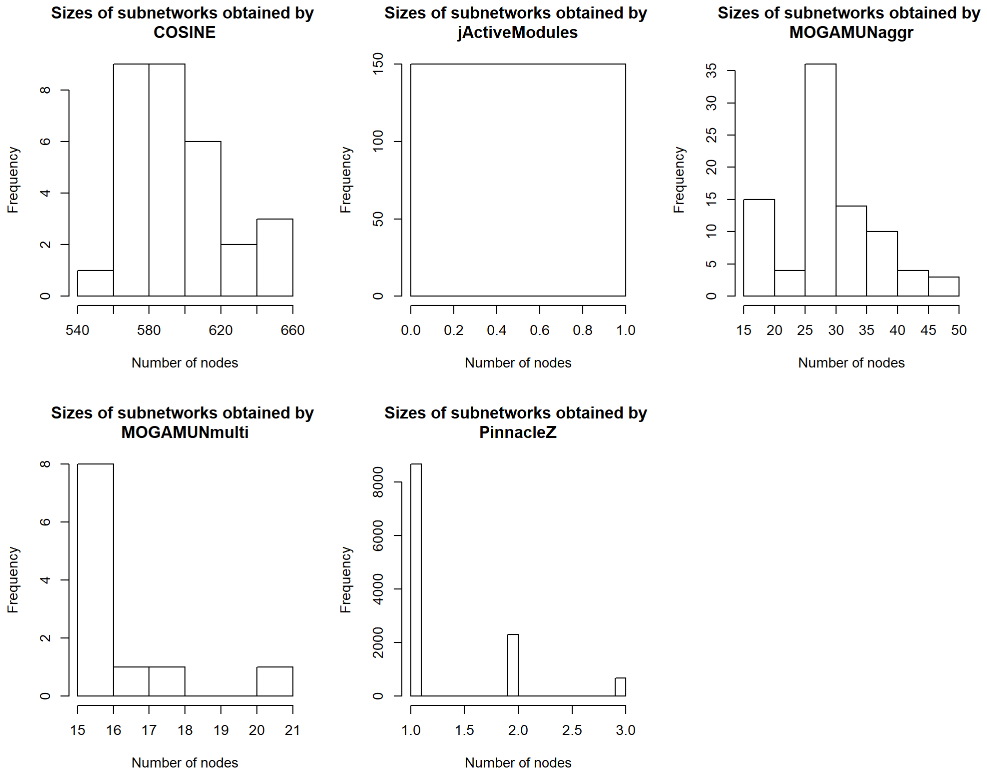

Fig S23: Sizes of the subnetworks identified by the five approaches on Yao's dataset, myoblasts

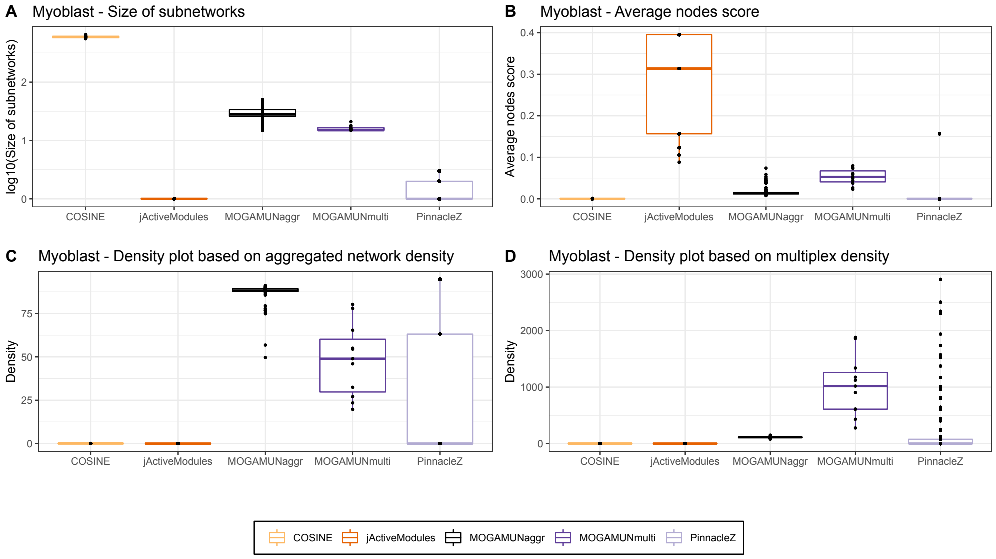

Fig S24: Size, average nodes score and density of the subnetworks obtained by the different methods, using Yao's dataset, myoblasts. The sizes of the subnetworks are represented on a log scale. The density is computed either on the aggregated network, corresponding to the union of the three biological networks used in this study, or using the multiplex-normalized density, proposed in the main manuscript

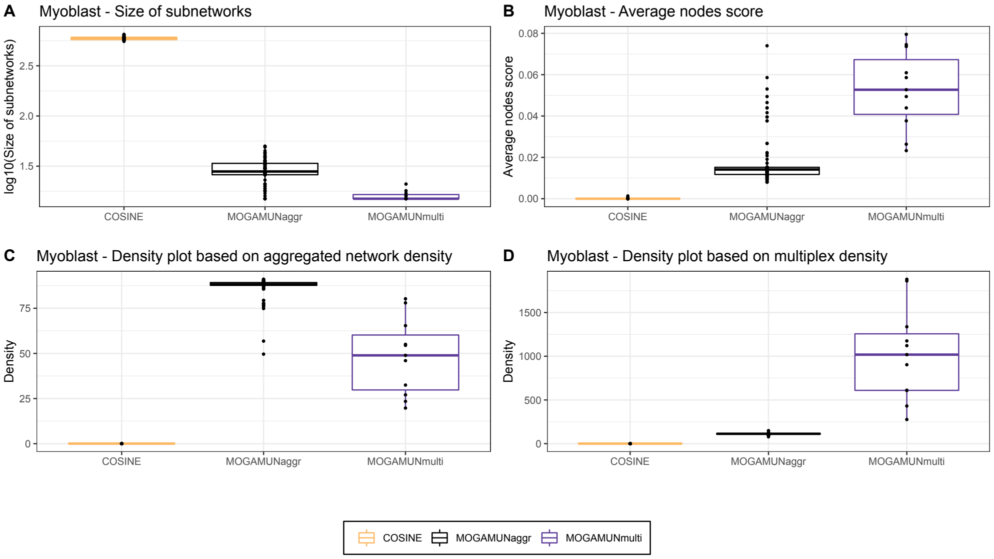

Fig S25: Size, average nodes score and density of the subnetworks obtained by the different methods, using Yao's dataset, myoblasts, selecting only the modules containing at least 15 nodes. The sizes of the subnetworks are represented on a log scale. The density is computed either on the aggregated network, corresponding to the union of the three biological networks used in this study, or using the multiplex-normalized density, proposed in the main manuscript

5.2 Active modules nodes statistics

We finally compared the total number of nodes and DEGs in the active modules retrieved (in 30 runs) by the different methods, in the three Yao's datasets (Supplementary Table S4). We did the same comparison focusing on the active modules containing at least 15 nodes (Supplementary Table S5). We observed that, in the myoblasts dataset, COSINE retrieved the highest proportion of DEGS, but this is at a cost of a very large number of nodes present in the identified active modules ($> 10\,000$ nodes), limiting the interpretability of the result. PinnacleZ, as previously observed, did not retrieve active modules composed of at least 15 nodes, and in most cases did not pick any DEGs. jActiveModules presented different behaviors, depending on the dataset under consideration. On the biopsies dataset, jActiveModules behaved similarly to MOGAMUN, with a total number of nodes > 100, most of which were ub active modules composed of at least 15 nodes. On the myoblasts dataset, jActiveModules identified active modules mainly composed of less than 15 nodes, mostly fewer than three nodes. Contrarily, on the myotubes dataset, jActiveModules identified very large active modules of about 2 000 nodes. MOGAMUN applied on the aggregated network (MOGAMUNaggr) also behaved differently depending on the dataset, with 55, 584 and 29 total nodes in the active modules retrieved in the biopsies, myoblasts and myotubes datasets, respectively. MOGAMUN using the multiplex network (MOGAMUNmulti) retrieved about 100 nodes in the active modules active modules of the three datasets.

Dataset	Method	Number of genes in active modules	Number of DEG in active modules	Total number of DEG in the dataset	% of DEGs retrieved in active modules
Biopsies	COSINE	14 178	2	6	33.33
	jActiveModules	131	4		66.66
	MOGAMUNaggr	55	3		50.00
	MOGAMUNmulti	109	4		66.66
	PinnacleZ	169	0		0
Myoblasts	COSINE	11 853	5	7	71.43
	jActiveModules	7	2		28.57
	MOGAMUNaggr	584	2		28.57
	MOGAMUNmulti	89	2		28.57
	PinnacleZ	1 867	0		0
Myotubes	COSINE	12 904	141	343	41.11
	jActiveModules	1 910	149		43.44
	MOGAMUNaggr	29	19		5.54
	MOGAMUNmulti	121	29		8.45
	PinnacleZ	899	16		4.66

Table S4: Number of genes and number and percentage of differentially expressed genes (DEGs) retrieved in the active modules by the different methods in 30 runs

Dataset	Method	Number of genes in active modules	Number of DEG in active modules	Total number of DEG in the dataset	% of DEGs retrieved in active modules
Biopsies	COSINE	14 178	2	6	33.33
	jActiveModules	127	4		66.66
	MOGAMUNaggr	55	3		50.00
	MOGAMUNmulti	109	4		66.66
	PinnacleZ	0	0		0
Myoblasts	COSINE	11 853	5	7	71.43
	jActiveModules	0	0		0
	MOGAMUNaggr	584	2		28.57
	MOGAMUNmulti	89	2		28.57
	PinnacleZ	0	0		0
Myotubes	COSINE	12 904	141	343	41.11
	jActiveModules	1 905	144		41.98
	MOGAMUNaggr	29	19		5.54
	MOGAMUNmulti	121	29		8.45
	PinnacleZ	0	0		0

Table S5: Number of genes and number and percentage of differentially expressed genes (DEGs) retrieved in the active modules by the different methods in 30 runs. Only the statistics of the subnetworks with at least 15 nodes are reported here

References

1. Yao Z, Snider L, Balog J, Lemmers RJ, Van Der Maarel SM, Tawil R, et al. DUX4-induced gene expression is the major molecular signature in FSHD skeletal muscle. Hum Mol Genet. 2014; 23: 5342-5352.

2. Banerji CR, Panamarova M, Hebaishi H, White RB, Relaix F, Severini S, et al. PAX7 target genes are globally repressed in facioscapulohumeral muscular dystrophy skeletal muscle. Nat Commun. 2017; 8: 1-13.

3. Banerji CR, Panamarova M, Pruller J, Figeac N, Hebaishi H, Fidanis E, et al. Dynamic transcriptomic analysis reveals suppression of PGC1 α/ERR α drives perturbed myogenesis in facioscapulohumeral muscular dystrophy. Hum Mol Genet. 2019; 28: 1244-1259.

4. Deb K, Pratap A, Agrawal S, Meyarivan TAMT. A fast and elitist multiobjective genetic algorithm: NSGA-II. IEEE Trans Evol Comput. 2002; 6: 182-197.

5. Eiben AE, Smit SK. Evolutionary algorithm parameters and methods to tune them. In: Hamadi Y, Monfroy E, Saubion F, editors. Autonomous search. Springer, Berlin, Heidelberg; 2011. pp. 15-36.

6. Eiben AE. Smith JE. Introduction to Evolutionary Computing. 2nd ed. Springer; 2015: 245-258..

7. Hamblin S. On the practical usage of genetic algorithms in ecology and evolution. Methods Ecol Evol. 2013; 4: 184-194.

8. Ideker T, Ozier O, Schwikowski B, Siegel AF. Discovering regulatory and signalling circuits in molecular interaction networks. Bioinformatics. 2002; 18: S233-S240.

9. Chuang HY, Lee E, Liu YT, Lee D, Ideker T. Network-based classification of breast cancer metastasis. Mol Syst Biol. 2007; 3: 140.

10. Nikolayeva I, Guitart Pla O, Schwikowski B. Network module identification—A widespread theoretical bias and best practices. Methods. 2018; 132: 19-25.
