# Supplementary material for: A multi-objective genetic algorithm to find active modules in multiplex biological networks
Source: PLoS Comput Biol. 2021 Aug 30;17(8):e1009263. doi: 10.1371/journal.pcbi.1009263 (PMC8452006; doi:10.1371/journal.pcbi.1009263)
Supplement: Attachment — Submitted filename: Response to reviewers -MOGAMUN-.pdf [file pcbi.1009263.s002.pdf]

Reviewer # 1:

The work claims to present a first of its kind way to identify active sub-networks in a network where there are multiple types of edges-- multiplex networks:

The objective of MOGAMUN is to integrate expression and network data to identify active modules. To the best of our knowledge, the originality of our work is to propose a multi-objective algorithm for this very task. Our method has also been specifically designed to leverage RNA-seq data, whereas the majority of previous approaches used microarray data. Finally, we allow the users to input more than one biological network and thereby leverage multiple complementary sources of interactions. This last point is not considered by other currently available approaches.

There is a disconnect between the paragraphs where the explanation of Equation (4) ends and the explanation of the flowchart begins.

We enhanced the organisation of this section. We divided it into 2 subsections and added the following subtitles to smoothen the transition: i) Definition of the objective functions and ii) General workflow of MOGAMUN.

There needs to be a brief overview of what exactly NSG-II does, and why it is possible and viable to adapt it to work with multiplex networks.

We agree that the description of the NSGA-II algorithm was too sparse in the initial version of the paper. We added the following description of NSGA-II in the manuscript on page 4, in Section 2.1:

“NSGA-II is a widely used multi-objective genetic algorithm-based optimization method. NSGA-II uses non-dominated sorting to rank the solutions, which allows optimizing two objective functions simultaneously (see Supplementary Section 3 for details).”.

In addition, we created a Supplementary Methods section where the NSGA-II algorithm is described in detail (Supplementary Section 3).

Concerning our adaptations of NSGA-II to deal with networks, importantly, this adaptation was possible because we formulated the search for active modules as a multi-objective optimization problem. In addition, NSGA-II allows a precise control of the individuals' generation. In our case, we generate the individuals using the Depth-First-Search algorithm, and we specifically extended this algorithm to work on multiplex networks. Moreover, we customized NSGA-II genetic operators of crossover and mutation. We designed those operators so as to guarantee that the individuals always remain connected subnetworks, as described in our method section.

There are contractions, expansions of which are nowhere in the text. The first usage of a contraction needs to have the full name and explanation.

We carefully checked the paper for acronym definitions, and extended them (highlighted in blue in the manuscript). We kept RNA, CRISPR, GWAS, NP-hard in short acronym forms to avoid making the text heavier as these acronyms are commonly used in the community.

Another work, a brief evaluation of which should be in this work: DIRAC: Identifying Tightly Regulated and Variably Expressed Networks by Differential Rank Conservation (DIRAC) by James A. Eddy, Leroy Hood, Nathan D. Price, and Donald Geman, published in PLOS Computational Biology in 2010.

We thank the reviewer for pointing out the DIRAC publication. DIRAC is a method dedicated to the identification of perturbed pathways between phenotypes. It makes pairwise comparisons of the expression of the genes in a pathway to find their ranking (ordering), from the most to the least expressed, without using network topology data. DIRAC then compares this ranking to find those pathways that are affected across phenotypes. Overall, DIRAC can be considered as a method belonging to the field of pathway enrichment analysis. However, DIRAC would not be able to handle large-scale networks nor consider their topology together with expression data in the same way as active module identification methods such as MOGAMUN, COSINE, jActiveModule or PinnacleZ do.

Indeed, MOGAMUN and the methods we are focusing on in our manuscript belong to the category of active module identification methods. Even if the ultimate goal of such active module identification approaches is also to identify perturbed biological processes, they are different from pathway enrichment approaches. To review the state-of-the-art on active module identification and select the approaches we used for comparison, we took advantage of a review from Trey Ideker's lab specifically dedicated to active module identification (Mitra et al. 2013). These methods are considered as “Active-module detection through network projection of omics data" (see Table 1 of Mitra et al. 2013).

In the field of network biology, in addition to active module identification approaches, we acknowledge that other types of approaches are widely used. This includes the pathway enrichment approaches, including DIRAC and many others. We have for instance been previously working on such pathway enrichment approaches using network information (Glaab, Baudot et al. 2012). These approaches are mentioned in our introduction and associated with the Reimand et al. 2019 review (reference [1] in our manuscript). The exercise for MOGAMUN and the other methods targeted in our current manuscript is different as the goal is to identify active modules by integrating expression and network information.

References

Glaab, E., Baudot, A., Krasnogor, N., Schneider, R., & Valencia, A. (2012). EnrichNet: network-based gene set enrichment analysis. *Bioinformatics*, *28*(18), i451-i457.

Mitra, K., Carvunis, A. R., Ramesh, S. K., & Ideker, T. (2013). Integrative approaches for finding modular structure in biological networks. *Nature Reviews Genetics*, *14*(10), 719-732.

Reimand,J.et al. (2019) Pathway enrichment analysis and visualization of omics data using g:Profiler, GSEA, Cytoscape and EnrichmentMap. Nat Protoc, 14, 482.

Were tests done to explore the application to FSHD1, using the three methods for comparison: jActive, PinnacleZ and COSINE? If yes, how do those compare with MOGAMUN?

In the initial version of our manuscript, we compared the different methods using the simulated benchmark experiments 1&2, but applied only MOGAMUN to FSHD1 to provide an illustration of the approach. We made this choice mainly because i) the three other approaches (jActiveModule, PinnacleZ and COSINE) cannot handle multiplex networks, and ii) on the FSHD1 use-case, we do not have ground-truth modules to be retrieved. FHSD1 is a poorly deciphered disease from a molecular pathophysiology perspective, and the evaluation of the relevance of the identified modules was done by checking the literature with biologists experts of the disease. We thereby initially decided to compare the methods in a controlled setting, i.e., using a monoplex network and one module to identify in simulated benchmark experiments.

We however agree with the reviewer that a comparison of the results obtained by the different methods on FSHD1 data could be of interest to apprehend the behavior of the different approaches in real-case settings. We hence decided to conduct additional experiments following the reviewer's suggestion. In addition to MOGAMUN, we ran the three other active module identification approaches as well as MOGAMUN on the aggregated version of the multiplex network on the three datasets from Yao et al. RNA-seq study. These additional experiments necessitated more than 3,000 hours of computing in total. Overall, we can observe that MOGAMUN is the best approach to output active modules that can be interpreted from a biological point of view, as the other approaches have a tendency to either give very small modules composed of very few nodes or very large modules composed of thousands of nodes on this real-case setting. These experiments are now described in a new section of the supplementary material (Supplementary Section 5), and referenced in the Results section of our manuscript.

Overall, the paper is well-written and is easy to follow.

We thank the reviewer for this encouraging comment.

Reviewer # 2:

The paper deals with a multi-objective optimization and presents a new algorithm, called MOGAMUN, for searching active modules in multiplex networks.

Line 267: "We elected" (We selected??)

We corrected the sentence to “We selected the total number of generations empirically, after running the algorithm several times in different contexts and monitoring its convergence.”

I suggest to merge subsections 2.2.3 - 2.2.6 into one subsection.

We think the reviewer was referring to subsections 2.3.3 to 2.3.6. We took the reviewer's suggestion on board to improve the logical flow of the section and merged the sections into a single one (2.3.3).

Put the caption in Table 4 on the top of the table.

Done.

Reviewer # 3:

In this manuscript, the authors proposed MOGAMUN, a multi-objective genetic algorithm to find active modules in multilayer networks. The initial population is generated from a set of selected genes such as differentially expressed genes, then in each step optimization is done using two objective functions that optimize node scores and density of subnetworks. Node scores are defined based on p-values obtained mainly from differential expression analysis. The proposed method is evaluated by comparing against three other methods on simulated single-layer networks.

General comments:

1. Authors claim “MOGAMUN is the first method able to use multiplex networks”. This is not an accurate claim. There are previous studies that have already covered this topic. Several such references are given below in the specific comments section. Therefore, it needs to be mentioned clearly what are the novel aspects of this work.

MOGAMUN and the methods we are focusing on in our manuscript are categorized as active module identification approaches. These methods are specifically dedicated to the identification of subnetworks of interest thanks to the integration of expression data (or other types of omics quantitative information) and biological networks. To review the field and identify methods for comparison, we leveraged in particular the authoritative review from Trey Ideker’s lab (Mitra et al. 2013), where these methods are named “Active-module detection through network projection of omics data". Importantly, such approaches are different from the approaches dedicated to clustering/community identification, which rely only on network topology as an input to identify modules. The fact that the same semantic “module” is used in the literature to discuss both topological modules and active modules is a point of confusion.

We have been involved for many years in the development of clustering algorithms for the identification of communities from the network topology alone, and we also developed clustering algorithms that can leverage multiplex networks for this task (Didier et al. 2015, Didier et al. 2018). We are moreover participants and authors of the DREAM disease module identification challenge mentioned by the reviewer. The goal of this DREAM challenge was to identify clusters/communities from network topology alone: during the challenge, we were not provided and did not integrate any context-specific expression data into the networks. We carefully checked all the references provided by the reviewer (summaries of the publications are provided in the answer to specific comments), and we think they all belong to the category of methods

dedicated to clustering/community identification. Their goal is thereby different from the focus of our paper here which is active module identification.

We went back to check the literature for active module identification algorithms, and did not identify any method integrating expression/omics data with multiple sources of interactions to identify active modules. We hence think that MOGAMUN is novel for the task considered in this manuscript. Very recently (beginning of 2021), Ron Shamir's lab published an evaluation of 6 active module identification algorithms using gene expression and GWAS data (Levi et al. 2021). None of the methods evaluated in this paper exploit multiple network sources as inputs.

References

Didier, G., Brun, C., & Baudot, A. (2015). Identifying communities from multiplex biological networks. *PeerJ*, *3*, e1525.

Didier, G., Valdeolivas, A., & Baudot, A. (2018). Identifying communities from multiplex biological networks by randomized optimization of modularity. F1000Research, 7.

Choobdar,S… DREAM module identification challenge consortium et al. (2019) Assessment of network module identification across complex diseases. Nat. methods, 16, 843-852.

Levi, H., Elkon, R., & Shamir, R. (2021). DOMINO: a network‐based active module identification algorithm with reduced rate of false calls. *Molecular Systems Biology*, *17*(1), e9593.

Mitra, K., Carvunis, A. R., Ramesh, S. K., & Ideker, T. (2013). Integrative approaches for finding modular structure in biological networks. *Nature Reviews Genetics*, *14*(10), 719-732.

2. Using density of subnetworks does not seem to be a good choice for objective function. Based on definition of objective function, a group of small dense subnetworks of a module may have higher score than the actual module. As a result, the algorithm might get stuck in local optima split the actual module to smaller submodules. A module should satisfy both conditions: i) dense connections among nodes in the module; ii) fewer connections with other nodes in the graph. However, in this work it seems like little attention is paid to the second condition.

We think that the density function is a good choice to solve the question we are asking with our algorithm, namely the identification of active modules. Regarding the first part of the reviewer's comment concerning module size, we completely agree with the reviewer. The normalized density that we defined is indeed higher for smaller modules, and the MOGAMUN algorithm will thereby have a tendency to promote the identification of smaller subnetworks. This is the reason why we provide a customizable parameter for the minimum allowed size of the modules to be reported.

In our case, after discussing with our biologist colleagues, we decided to select modules that contain 15 or more nodes for biological interpretation. As shown in the results, most of the

subnetworks identified by MOGAMUN have about 15 nodes, which correspond to the minimum size we allowed, and confirm the reviewer's observation. We are aware that users could have different needs, and we added a sentence to clarify this point and invite the readers to tune this parameter according to their needs. The parameter section 2.2 now states: "The minimum size parameter is important as real biological networks are sparse and the density definition will tend to favor smaller subnetworks. The final subnetworks will tend to have sizes equal to the minimum size allowed."

Regarding the second part of the reviewer's comment concerning the dual condition of high number of internal edges and low number of external edges, we consider that this is not the case for active module identification algorithms. Indeed, these two conditions should both be fulfilled for community identification or clustering algorithms, where the goal is to cluster the network nodes based on topology alone. Clustering methods for community detection have been widely used to identify modules in networks and define cellular building blocks. But they provide a static view of the cellular systems and miss the variations that can occur in different cellular conditions (Mitra et al. 2013). Methods for active module identification were developed to tackle this challenge and identify subnetworks relevant for the molecular profiles under study, but not to find "isolated" subnetworks from a topological point of view. According to Mitra et al. 2013, the terms network hotspots or responsive subnetworks are also used, but active modules seem the most commonly used term. It is to note however that some approaches can be considered as in between topological and active module identification algorithms. This is the case for instance of the recently published method DOMINO (Levi et al. 2021).

Most of the active module identification approaches we checked did not try to optimise the density or even the number of interactions of the subnetworks to be identified, but only checked for the connectivity of the output subnetworks. While developing MOGAMUN, we also wanted to use the density as one of the two objectives, in order to enrich our results for regions containing a lot of interactions that might be easier to interpret from a biological point of view. In addition, active module identification approaches based on the aggregation of a normalized score are known to be biased towards the identification of very large modules (Nicolayeva et al. 2018). Introducing the density as a second objective allows controlling this bias. But if these network regions are also densely connected with other regions is not a problem for the task of active module identification.

References

Mitra, K., Carvunis, A. R., Ramesh, S. K., & Ideker, T. (2013). Integrative approaches for finding modular structure in biological networks. *Nature Reviews Genetics*, *14*(10), 719-732.

Nikolayeva, I., Guitart Pla, O. & Schwikowski, B. Network module identification—A widespread theoretical bias and best practices. *Methods* **132**, 19–25 (2018).

Levi, H., Elkon, R., & Shamir, R. (2021). DOMINO: a network-based active module identification algorithm with reduced rate of false calls. *Molecular Systems Biology*, *17*(1), e9593.

3. The evaluation of the proposed method is not rigorous. For example, comparison with other methods is based on two networks with only one subnetwork. More realistic datasets with multiple active modules are needed. In addition, in Figure 2, methods are compared based on density and node scores which are the metrics that have been specifically optimized using MOGAMUN, and therefore not a fair comparison. More importantly, since this method is dedicated to work on multiplex networks the comparison should also be on multiplex network, not on single network.

We acknowledge that our benchmark has different limitations, we were aware of them and made different choices that we will explain below to make our benchmark as pertinent and fair as possible. After having developed our algorithm, we checked the literature for existing benchmarks in the field of active module identification algorithms. We identified two types of strategies to evaluate the performances of the different algorithms: i) benchmarks using simulated datasets and ii) benchmarks using biological proxies. We decided to devise a benchmark based on simulated data, as in Batra et al. (2017), in order to have a ground-truth to evaluate the outputs from the different approaches. We are discussing this choice below in light of the different comments made by the reviewer.

Point #1 => only one module to detect

This is a limitation we indeed considered when developing our benchmark. However, we had two major obstacles when trying to generate two or more ground-truth subnetworks with differential expression signals to be detected by the different approaches. First, the COSINE approach is not designed to retrieve more than one module in a given run. Second, we tried to generate multiple ground-truth subnetworks disconnected from each other. However, we are using real protein-protein interaction networks in our benchmark (PPI_1 and PPI_2). Biological networks are small-world, and our attempts to simulate completely separated sets of foreground genes were unfruitful, as we systematically obtained one merged subnetwork. For these two reasons, we decided to simulate only one subnetwork.

Point #2 => unfair comparisons in Figure 2 since it's our objective functions that are plotted

First, we acknowledge that the plot represents our two objective functions, and that the overall strategy of the other methods we used intend to optimise only one of these functions, namely the overall deregulation of the subnetwork represented here by the Average nodes score. Figure 2 (now Figure 3 in the revised version) was initially dedicated to represent the diversity of the solutions obtained by MOGAMUN rather than providing a metric to promote the performance of MOGAMUN. Indeed, as MOGAMUN is a multi-objective approach, it allows exploring a large diversity of trade-off solutions and Figure 2 (now Figure 3) allows checking that the method is behaving as expected: in multi-objective optimization, we cannot say that a solution is better than another if it has a better value for one of the two objectives only.

In the manuscript, the comparison of the performance of the different approaches was relying mainly on the F1-scores of the solutions provided by the different approaches, considering both

precision and recall. These results were described in the text and were available in Supplementary Figure S9. MOGAMUN performs better than the other three tested methods in terms of F1-scores in the two benchmark experiments. In order to clarify this point, we now highlight these results by moving Supplementary Figure S9 into the main manuscript (now Figure 2). We also clarify the legend of the Figure 2 (now Figure 3) and specify in the text that MOGAMUN is the only method with a density optimisation, and thereby is expected to be better in terms of density of the obtained modules, by adding the sentence “This is expected as MOGAMUN is the only approach also optimizing subnetwork densities".

Point #3 => benchmarking of multiplex networks

This is another important point we considered while designing our benchmark. We faced two obstacles. First, MOGAMUN is the only method specifically designed to handle multiple sources of interactions. Indeed, jActiveModules, COSINE and PinnacleZ (and, to the best of our knowledge, all other active module identification methods) can only consider one network as an input. We would have been able to circumvent this issue by feeding MOGAMUN with a multiplex network, and aggregating this multiplex in a unique ‘monoplex’ network to feed the three other tools. However, this strategy raises an important issue: how to simulate a set of foreground genes from a multiplex network? On the one hand, if we had simulated the foreground genes from the aggregated monoplex network, we would not have accounted for the complementarity of the different interaction sources and would have favoured any monoplex methods. On the other hand, if we had simulated the foreground genes from a multiplex network (using the multiplex Depth First Search we created, for instance), it would have favored the only multiplex method, MOGAMUN. Overall, comparing multiplex and monoplex methods would in any case favor some approaches. To avoid unnecessary complications and potential difficulties in the interpretation of the results, we decided to implement a straightforward and simple benchmark using one network and one module to be retrieved.

However, in the revised version of our manuscript, we extended our evaluations and used the FSHD1 datasets to evaluate the results obtained by the different methods in several real-case settings. In addition to the classical MOGAMUN approach, we ran the three other active module identification approaches as well as MOGAMUN on the aggregated version of the multiplex network on the three datasets from Yao et al. RNA-seq study. These additional experiments necessitated more than 3,000 hours of computing in total. In FSHD1, we do not have ground-truth modules available, but we compared the active modules identified by the different methods in terms of different features such as their number, sizes, density, Average nodes score and the number of DEGs retrieved in the subnetworks. Overall, we can observe that MOGAMUN is the approach providing the best trade-off, as it allows retrieving modules that can be used for further biological interpretation. The other approaches either give very small modules composed of very few nodes (typically two to three) and/or a very large module composed of thousands of nodes for each run. These experiments are now described in detail in a new section of the Supplementary Material (Supplementary Section 5), with in particular 9 new Supplementary Figures and two new Supplementary Tables, and referenced in the Results section of our manuscript.

Reference

Batra R, Alcaraz N, Gitzhofer K, Pauling J, Ditzel HJ, Hellmuth M, et al. On the performance of de novo pathway enrichment. NPJ Syst Biol Appl. 2017 Mar 3.

Specific comments:

1. The results of “community identification dream challenge” showed that dedicated methods for complex network fail to significantly improve the task of module discovery compared to methods that considered each network individually. I am interested to see how your method works on multiplex networks compared to methods that are designed to work on single networks. Two possible strategies for single network methods to be applied on multiplex networks are merging networks before module discovery or consensus analysis after applying method on individual network. In fact, the second objective function seems to be equivalent to pre-merging the networks after normalizing the edge weights in each network by the overall network density. With the way that the networks are simulated, it may be easy to simply compute differential expression, and then looking for densely connected subnetworks from the top DE genes.

Point #1 Methods for complex network fail to significantly improve the task of module discovery compared to methods that considered each network individually

First of all, we (AB, LT) were participants of the DREAM challenge dedicated to disease module identification and we are authors of the resulting consortium paper. In fact, we were amongst the best performers of the multiplex sub-challenge (tied second). This challenge was dedicated to the identification of communities based on network topology alone, which is a different task to the one targeted in the current paper by MOGAMUN and the three other approaches.

In the DREAM challenge, we agree that the different multiplex approaches using more than one network as input did not significantly outperform the best monoplex approach on a single network. However, we can qualify the discussion. First, the best performers in the multiplex sub-challenge identified 21 communities as a result of using all the layers of the multiplex networks, whereas the best performer of the monoplex network identified 20 communities. The organisers decided to compare the monoplex results to random predictions, but the multiplex results to the best performers of the monoplex, hence comparing very different mathematical frameworks. Indeed, our “internal” comparison on the DREAM challenge benchmark, that is, the comparison of our own algorithm (based on the optimisation of the modularity or the multiplex-modularity with the Louvain algorithm), revealed that we identified more significant disease modules with the multiplex approach than with the monoplex approach using the same mathematical framework. These results are published in our companion paper to the DREAM challenge (Didier et al. 2018). Another point of interest is that the results of the DREAM challenge were highly sensible to very small changes in the submitted modules, because the

evaluations were based on enrichment analyses, which are highly sensitive to module size and multiple testing corrections. Finally, our main conclusion is that a multiplex strategy is optimal when the networks represent “instances” of the same community structure, which was not the case in the DREAM challenge as some of the networks represented different biological information (such as protein-protein interactions that occur more inside signaling pathways and epistatic interactions that occur more in between different signaling pathways).

Overall, multiplex and multilayer frameworks have been shown to provide more complex but richer information using different network algorithms (Kivelä et al. 2014). We have shown the pertinence of the multiplex approaches in biology for community identification using simulated networks with Stochastic Block Models (Didier et al. 2015), with Random Walk with Restart (Valdeolivas et al. 2019), and many teams, in particular in the physics community, are working on this topic and established the pertinence of the multilayer framework in different contexts (De Domenico et al. 2014, De Domenico et al. 2016, Halu et al. 2019).

On the topic of active module identification, - the very goal of MOGAMUN and of the other methods used in the current manuscript - it would indeed be interesting to evaluate the added value of using multiple sources of interactions as compared to either merging the networks or applying the method on different layers and finding a consensus approach. As explained in a previous point of the answer to the reviewer, we were not able to derive a benchmark from simulated data that would generate a fair ground-truth “active module” for comparing multiplex and monoplex approaches in a single benchmark. In addition, consensus methods would imply the definition and testing of consensus approaches, which are beyond the scope of the current work.

Yet, we took on board the idea suggested by the reviewer to test the methods with multiplex networks. In the new Supplementary Section 5 of our manuscript, we tested the different active module identification approaches on an aggregated version of the multiplex network. In addition, we also compared MOGAMUN applied on this aggregated network (MOGAMUNaggr) to MOGAMUN applied on the multiplex network (MOGAMUNmulti). The only difference between the two versions of MOGAMUN will be in the computation of the density function, which is the classical density on the aggregated network and the normalized density on the multiplex network. As expected, the density of the obtained active module is different for the two versions of MOGAMUN: MOGAMUNaggr retrieves denser subnetworks than MOGAMUNmulti if the classical density is computed, whereas MOGAMUNmulti retrieves denser subnetworks than MOGAMUNaggr if the normalised density is computed (Supplementary Figures S17.2C-D, S18.2C-D, S19.2C-D). We observed that MOGAMUNaggr also identifies modules with better Average nodes score than MOGAMUNmulti in 2 of the 3 datasets. We hypothesise that this comes from the fact that the aggregated network can more easily connect DEGs with good values as it contains much more interactions, but that these interactions will come in majority from the co-expression layer and thereby provide results less interpretable from a biological point of view, as we observed for community detection (Didier et al. 2015).

References

Choobdar,S… DREAM module identification challenge consortium et al. (2019) Assessment of network module identification across complex diseases. Nat. methods, 16, 843-852.

De Domenico, M., Solé-Ribalta, A., Gómez, S., & Arenas, A. (2014). Navigability of interconnected networks under random failures. *Proceedings of the National Academy of Sciences*, *111*(23), 8351-8356.

De Domenico, M., Granell, C., Porter, M. A., & Arenas, A. (2016). The physics of spreading processes in multilayer networks. *Nature Physics*, *12*(10), 901-906.

Didier, G., Brun, C., & Baudot, A. (2015). Identifying communities from multiplex biological networks. *PeerJ*, *3*, e1525.

Didier, G., Valdeolivas, A., & Baudot, A. (2018). Identifying communities from multiplex biological networks by randomized optimization of modularity. *F1000Research*, *7*.

Halu, A., De Domenico, M., Arenas, A., & Sharma, A. (2019). The multiplex network of human diseases. *NPJ systems biology and applications*, *5*(1), 1-12.

Kivelä, M., Arenas, A., Barthelemy, M., Gleeson, J. P., Moreno, Y., & Porter, M. A. (2014). Multilayer networks. *Journal of complex networks*, *2*(3), 203-271.

Valdeolivas, A., Tichit, L., Navarro, C., Perrin, S., Odelin, G., Levy, N., ... & Baudot, A. (2019). Random walk with restart on multiplex and heterogeneous biological networks. *Bioinformatics*, *35*(3), 497-505.

Point #2 The second objective function seems to be equivalent to pre-merging the networks after normalizing the edge weights in each network by the overall network density.

Indeed, we fully agree with the reviewer that the normalized density function we are using is equivalent to pre-merging the networks after normalizing the edge weights in each network by the overall network density. We compute the densities in each layer, weigh these densities (weighted by 1/layer_density) and then sum up the densities. This is equivalent to weighting all the edges of every layer (by 1/layer_density), merging all the layers (i.e. adding the weights) and computing the density. From a biological point of view, this allows us to consider the different sources of information without hiding small and curated networks inside big and noisy networks (such as the co-expression network). Such different strategies have been reviewed by Huang et al. 2021 (a paper mentioned by the reviewer) in the specific case of community identification algorithms.

In the context of active module identification, a good avenue for future research would be to consider specific edge weights for all the multiplex layers simultaneously. The different methods we are using in our manuscript for comparison however cannot handle edge weights.

Reference

Huang, X., Chen, D., Ren, T. & Wang, D. A survey of community detection methods in multilayer networks. Data Mining and Knowledge Discovery (2020) doi:10.1007/s10618-020-00716-6.

Point #3 With the way that the networks are simulated, it may be easy to simply compute differential expression, and then looking for densely connected subnetworks from the top DE genes.

We are not sure about the underlying idea of the reviewer here, as our networks are not simulated but real-case networks, and as we do not know any existing network algorithms that would allow extracting dense subnetworks around seeds/DEGs. Algorithms such as random walk with restart can extract subnetworks around seeds, but they do not intend to find dense subnetworks considering the global network topology. In addition, for RNA-seq datasets associated with many DEGs, extracting subnetworks around seeds/DEGs (for instance with random walk with restart strategies) would lead to a large number of subnetworks that would require post-processing. The final subnetworks would probably contain a lot of nodes but not necessarily be dense. In addition, the final subnetworks would not be optimal from a differential expression point of view. This is why the majority of the active module identification approaches try to optimise an aggregated node score (Nikolayeva et al. 2018).

In order to dig into the idea of local approaches focusing on top DE genes, we first checked the number of interactions from the different networks connecting the top DE (i.e. the significant DEGs). We focused on the three RNA-seq expression datasets from Yao et al. 2014 and the 3-layers multiplex network used in our FSHD1 application (see Table below).

Dataset from Yao et al. 2014	DEGs	Number of DEGs in the multiplex network	Number of DEGs connected together	Nb of edges per layer of the multiplex network		
				PPI	Pathways	Co-expression
Biopsies	6	4	0	0	0	0
Myoblasts	7	2	0	0	0	0
Myotubes	343	180	96	3	28	180

We identify only few direct interactions between DEGs.

We further explored the idea of an algorithm that would detect dense subnetworks around/starting from a given set of seeds, or maybe finding the densest subnetwork containing a given seed. This does not seem trivial. We could imagine devising a greedy search or simulated annealing around a seed, trying to maximize the density. Greedy search or simulated

annealing are actually used by PinnacleZ and jActiveModule in order to maximise the average nodes score. We could also make use of a community identification algorithm, for instance a method based on density such as CODENSE mentioned by the reviewer (Haiyan et al. 2005), to partition the full networks into clusters and then identify the clusters that contain the seeds. But this is different to the active module identification task, which aims to find subnetworks that are perturbed in different conditions. Overall, we did not identify any satisfactory solution to the proposal of the reviewer, or at least, it would implicate complex scenarios beyond the scope of the present work.

Reference

Haiyan Hu, Xifeng Yan, Yu Huang, Jiawei Han and Xianghong Jasmine Zhou, Mining coherent dense subgraphs across massive biological networks for functional discovery, Bioinformatics 2005.

Nikolayeva, I., Guitart Pla, O. & Schwikowski, B. Network module identification—A widespread theoretical bias and best practices. *Methods* **132**, 19–25 (2018).

2. What is the running time of MOGAMUN and how does it compare to other methods?

The execution times per run of each algorithm, in a desk computer with Intel processor i7 at 3.60GHz and 32GB of RAM, were approximately 30 min, 8 hours, 30 min, and 12 hours for jActiveModules, COSINE, PinnacleZ and MOGAMUN, respectively, as reported in section 3.1.

3. COSINE is another method based on genetic algorithm. In evaluation section some discussion on why MOGAMUN outperforms COSINE should be added.

MOGAMUN outperforms COSINE mainly because COSINE aims to identify highly deregulated and co-expressed genes, without considering the interactions in the network. Importantly, COSINE is a mono-objective optimisation method, which retrieves only a single subnetwork in a given run, whereas MOGAMUN is a multi-objective approach, which outputs subnetworks satisfying a trade-off between nodes scores and edges density at each run.

In the new version of our manuscript, we applied COSINE to three FSHD1 RNA-seq datasets, using as input an aggregated version of the multiplex network. These results are presented in the new Supplementary Section 5. We observed that, overall, COSINE identifies very large modules, containing thousands of nodes (see new Figures S17.1, S17.2A, S18.1, S18.2A, S19.1, S19.2A in the Supplementary Section 5). The union of the nodes retrieved by COSINE in 30 different runs is indeed in all cases of more than 10,000 nodes. In addition, we checked in detail the nodes identified by COSINE in the output subnetworks. We discovered that these subnetworks are composed of nodes found in the aggregated network, but also of nodes that are not part of the network, i.e. nodes that do not have any interaction in the biological network given as input. Furthermore, COSINE is using the raw expression data file and not the differential expression data file obtained from edgeR. We also found that some of the genes

identified by COSINE in the output subnetworks are not even considered as expressed according to edgeR. These details are shown in the pie charts below for the three considered expression data sets. For each COSINE module, the pie chart displays the proportion of genes that are retrieved in the network (GenesinAggregatedNetwork, in black), not retrieved in the network but found in the Differential Expression file (GenesinDEFile, in dark grey), and those genes that are neither in the network nor in the Differential Expression file but only in the raw expression data (GenesNotinDEFile, in light grey). We overall think that COSINE is not adapted to be used for RNA-seq data in real-case settings.

Pie charts Biopsy:

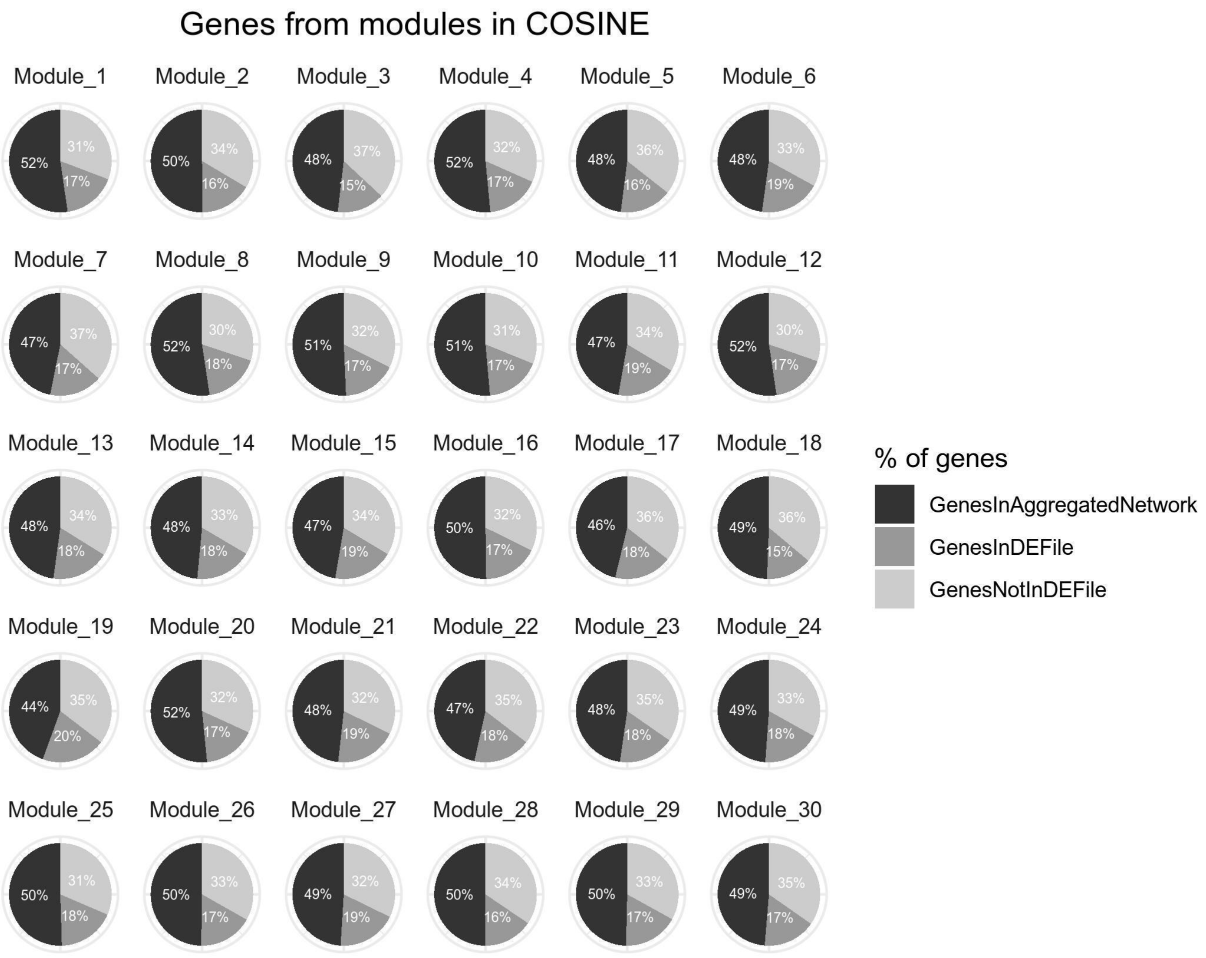

Pie charts Myoblast :

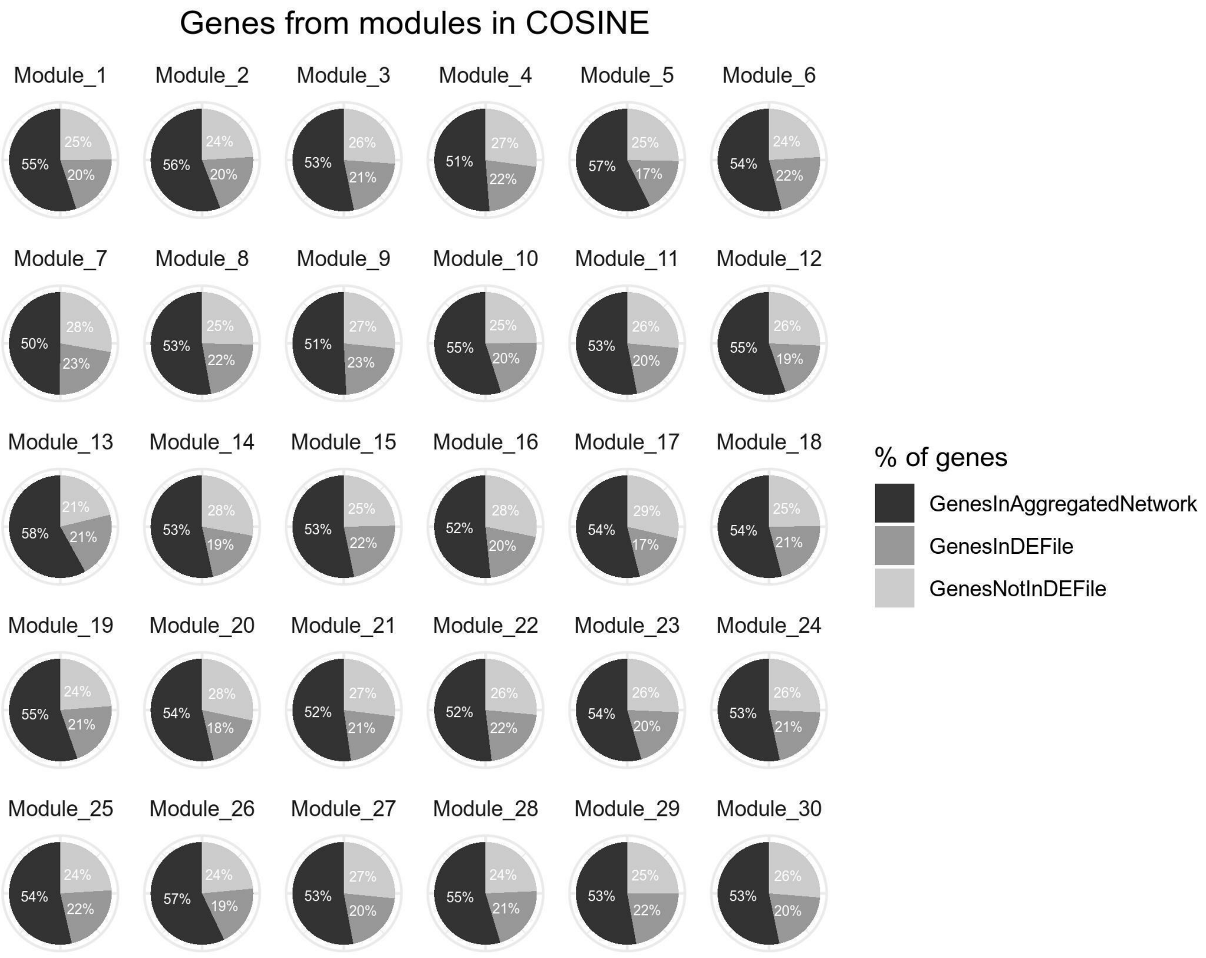

Pie charts Myotubes :

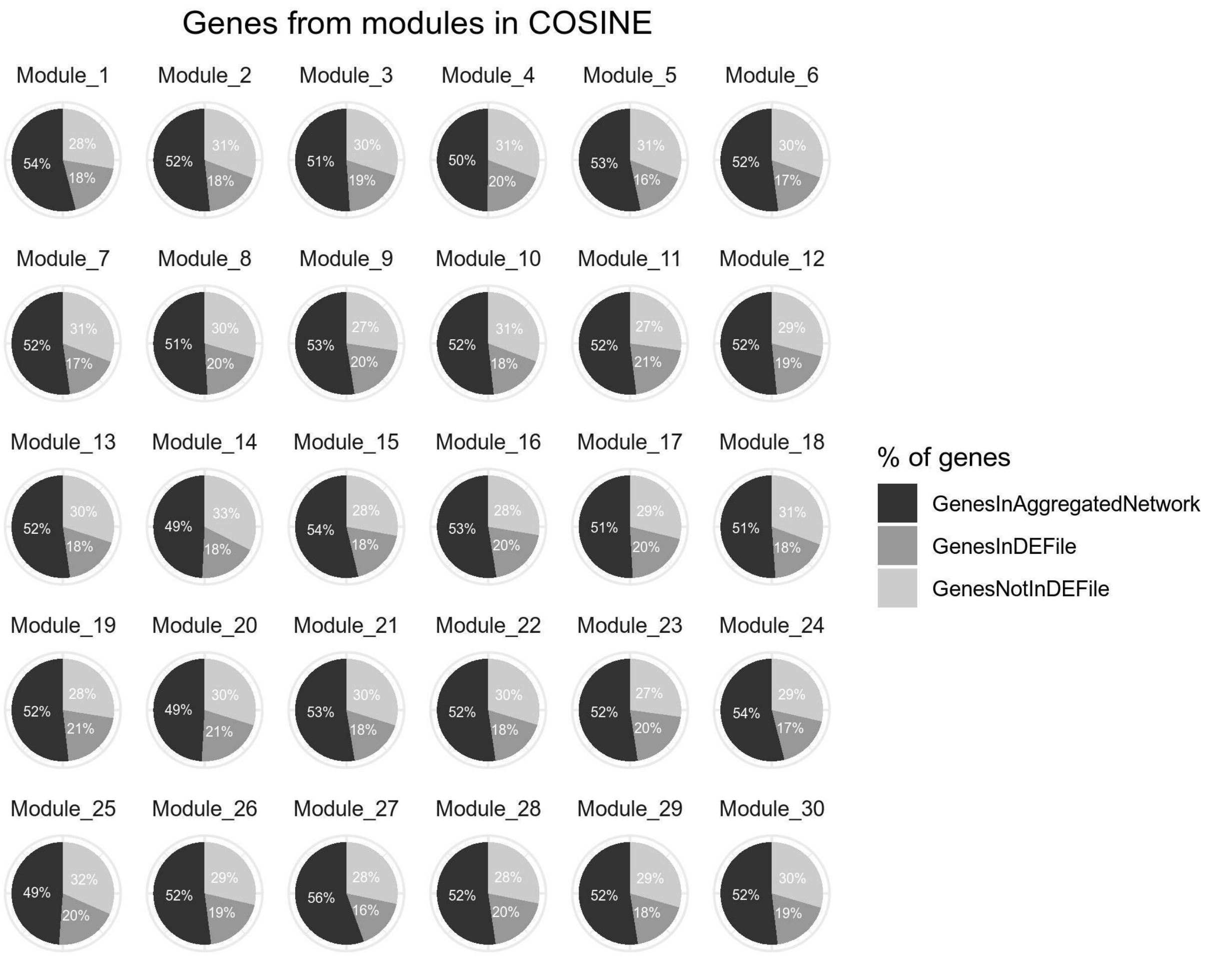

4. How the hyper parameters were tuned? Given the small dataset tested, it is unclear how these set of parameters can be selected for other networks with different structures and topology.

To answer this question, in the revised version of our manuscript, we applied MOGAMUN with different combinations of parameters on the myotube FSHD1 RNA-seq dataset (Yao et al. 2014). We used this dataset as it is the one associated with the highest number of DEGs. We ran MOGAMUN 30 times for each combination of parameters, testing all combinations of crossover rates from 0.7 to 1, and mutation rates from 0.1 to 0.3, both with increments of 0.1. These values correspond to the most common values and combination of values used for genetic algorithms (the crossover is the main operator while mutation is the secondary operator) (Eiben A. E. and Smith J. E., 2015; Eiben A. E., Smit S. K., 2011; Hamblin, S. 2013) . The first figure (Figure S15) presents the mean density and mean nodes score over 30 runs for the different sets of parameters. We empirically observe that the algorithm converges for all combinations of parameters, even if the rate of convergence varies.

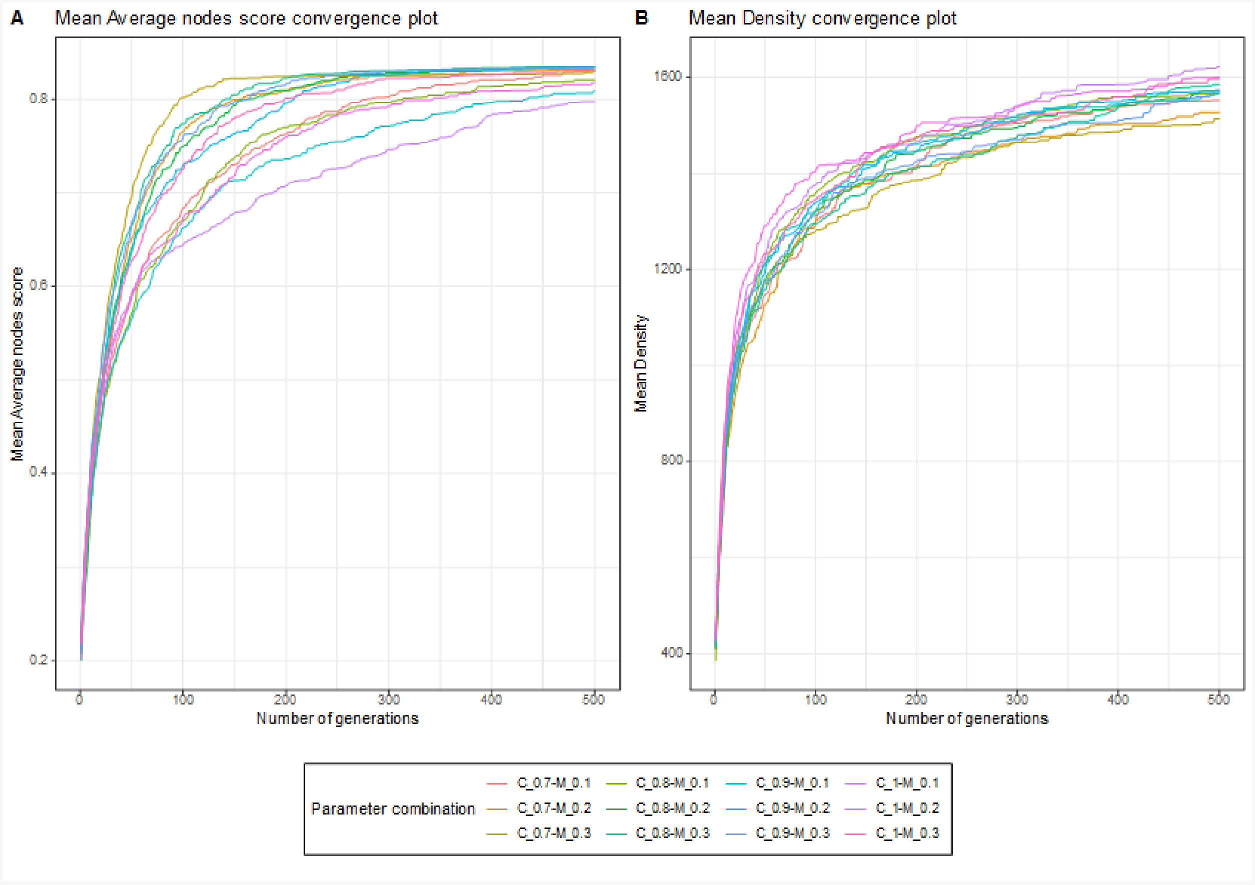

Figure S15

In addition, we represented the density and Average nodes score values obtained running MOGAMUN 30 times for these different sets of parameters as boxplots (Figure S16). We observe that the values of the two objective functions of the final subnetworks (active modules) are stable. Such behavior suggests that MOGAMUM is not significantly sensitive to its input parameters.

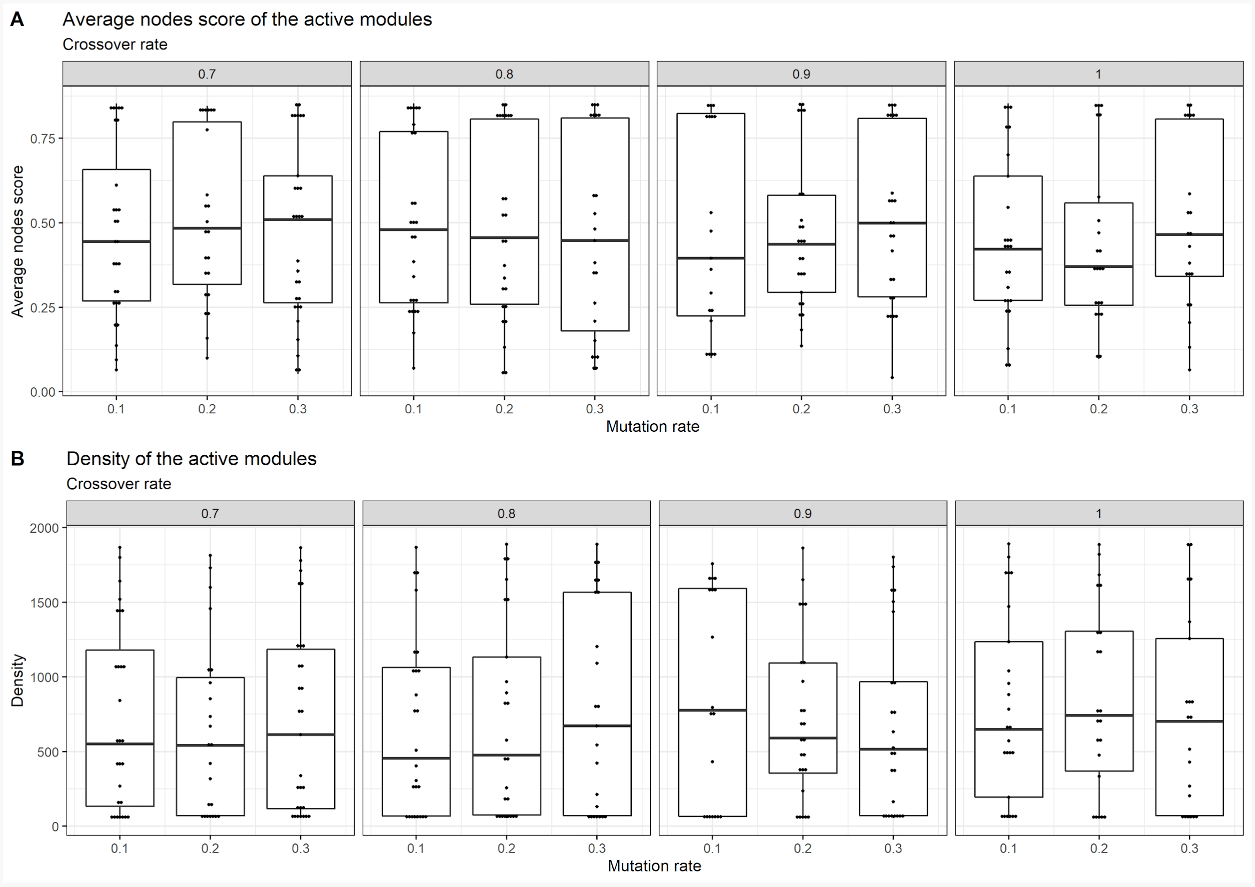

Eiben, A.E., Smit, S.K. (2011), Evolutionary algorithm parameters and methods to tune them. In: Autonomous search. Springer, pp 15–36

Eiben, A.E. and Smith J.E. (2015), Introduction to Evolutionary Computing, 2nd Edition, Springer.

Hamblin, S. (2013). On the practical usage of genetic algorithms in ecology and evolution. Methods in Ecology and Evolution, 4(2), 184-194.

5. How close different layers of the networks should be? Should they come from same context and same tissue? It seems the relation of different layers may have a big impact on the performance of the method. Do you have any elaboration on this?

This is an important point, which also relates to the previous discussion about the DREAM challenge. Our opinion is that the network layers should be complementary, meaning that they should emerge from the same underlying biological system. Each layer captures a different snapshot of the signal that occurs in the system. For instance, we expect that proteins in interaction will have a higher tendency to belong to the same signaling pathways and will be co-expressed. The biological interactions should not represent conflicting information such as protein-protein interactions (which have a tendency to belong to a single pathway) and epistasis (which often arise in between different signaling pathways). This point is (lightly) discussed in the DREAM challenge manuscript.

We are here working with “generic” networks, i.e., networks initially devoid of any spatio-temporal context, and providing static snapshots of the cellular system. The goal of active module identification is indeed to overlay expression data (or other types of molecular profiles, such as genomic, proteomic...) to these generic networks to evaluate context-specific subnetworks (Mitra et al. 2013, Levi et al. 2021).

The use of tissue-specific networks is an interesting point too. It could be relevant to apply the method for active module identification to different tissue-specific (multiplex or monoplex) networks, and analyse how the active modules detected vary according to the tissues, and maybe relate this point to the tissues affected by the pathology. A bottleneck in this approach is the construction of tissue-specific networks. Indeed, the methods for protein-protein interaction detection are not resolved at the tissue level, nor are pathway databases for instance. Different computational approaches have been developed to predict tissue-specific networks, but they are applied to protein-protein interactions only and, to the best of our knowledge, no multiplex networks with a tissue resolution exist so far (Greene et al. , 2015).

References

Choobdar,S… DREAM module identification challenge consortium et al. (2019) Assessment of network module identification across complex diseases. Nat. methods, 16, 843-852.

Greene, C. S., Krishnan, A., Wong, A. K., Ricciotti, E., Zelaya, R. A., Himmelstein, D. S., ... & Troyanskaya, O. G. (2015). Understanding multicellular function and disease with human tissue-specific networks. *Nature genetics*, *47*(6), 569-576.

Levi, H., Elkon, R., & Shamir, R. (2021). DOMINO: a network-based active module identification algorithm with reduced rate of false calls. *Molecular Systems Biology*, *17*(1), e9593.

Mitra, K., Carvunis, A. R., Ramesh, S. K., & Ideker, T. (2013). Integrative approaches for finding modular structure in biological networks. *Nature Reviews Genetics*, *14*(10), 719-732.

6. Some critical details are missing in the paper. For example, in section 2.3.2 describes how the expression levels for the FG genes are sampled, but did not describe how the FG genes are selected in the first place. Are they random genes in the PPI network? Most connected ones? The choices of the FG genes will directly affect the ability of the algorithms to find the module. For TCGA data, it is also confusing that you have to "sample" some gene expression data while there is (almost) a one to one correspondence between the PPI network and the RNAseq data.

Point #1 Selection of the FG genes

The FG (foreground genes) are selected as a connected subnetwork, using a seed-and-select algorithm, which generates a random connected subnetwork of a given size (section 2.3). The seed-and-select algorithm selects an initial seed and iteratively adds nodes one at a time, ensuring the subnetwork is connected, but ignoring the density. By doing so, we ensure that we do not favor MOGAMUN when creating the set of FG genes, as it is the only method also considering the density of the subnetwork. The FG genes are then associated with expression values simulated from a normal distribution.

We expanded the description of the seed-and-select algorithm in the devoted section:

"A *seed-and-select* algorithm is defined to randomly select the FG as a connected subnetwork. Such an algorithm selects a random seed and proceeds to iteratively add one node at a time to the subnetwork, by picking it up from the list of neighbors, such that the subnetwork remains connected. The process ends when the subnetwork reaches the desired size."

Point #2 TCGA sampling

In the particular case of TCGA, we did not do a one-to-one mapping of the gene expressions, because our goal was to simulate a ground-truth active module, i.e. a set of FG associated to differential expression that the methods would need to retrieve. We initially followed the same protocol as for the sim_normal dataset. First, we used the seed-and-select algorithm and selected randomly a connected 20-node subnetwork. The nodes from such subnetwork are the FG (foreground genes), while the rest of the nodes in the network are the BG (background genes). We then sampled expression data from TCGA for the FG and BG so that the expression data for the FG corresponded to significantly differentially expressed genes only, and that of the BG corresponded to non-significantly differentially expressed genes. By sampling from TCGA data, we ensured a correct distribution of the expression values of our FG and BG genes. Indeed, in our first scenario, the expression values of the FG genes and BG are sampled from normal distributions. But the RNA-seq expression values are known to not be normally distributed. We hence decided to add a second scenario, where the expression data of the FG and BG genes would be sampled from real RNA-seq expression data.

7. For general audience not familiar with genetic algorithms, it would also be helpful to add some details about tournament size, and crossover rate and how these parameter values impact the algorithm's behavior.

In order to provide more information for the general audience not familiar with genetic algorithms, we have added a full section in the supplementary material (Supplementary Section 3) describing the NSGA-II algorithm. In addition, as described previously, we now have added different tests to evaluate the behavior of MOGAMUN with regard to parameter tuning (see answer to specific comment #4). The results of the different tests on parameter tuning have also been added as a Supplementary Section (supplementary section 4) to provide these results to the reader.

8. The definition of multiplex network provided by the authors includes C, coupling links between layers. This is not explained, and seems to have not been used in the paper at all.

The coupling links C link every node with themselves across the different layers of the multiplex network (section 2.1). We use these links in two steps: (i) when switching between the different layers of the multiplex network in the Depth-First-Search (MakeDFS function, https://github.com/elvanov/MOGAMUN/blob/master/R/MOGAMUN_FUN.R) to generate the initial population and the children (line 9 in algorithm 1), and (ii) in the Breadth-First-Search (algorithm not shown in the manuscript, but code available in the function MakeBFS, in https://github.com/elvanov/MOGAMUN/blob/master/R/MOGAMUN_FUN.R) when generating the children, as mentioned in Section 2.1.

Since all the nodes are present in all the layers and since each node is connected to itself across the layers, the multiplex-DFS allows visiting different layers. When picking a layer from the multiplex network (see Algorithm 1, line 9), there is a 0.5 probability that the search will continue in the same layer. If the layer is to be changed, a new one is randomly picked up from a uniform probability distribution over the layers.

9. Table 3, why for sim_normal there are 483 DE genes? This suggests multiple testing correction is needed to pick the DE genes.

We indeed did not state clearly the reasons why using the DEGs with non-corrected p-values in this first experiment benchmark. In MOGAMUN, our goal is to optimise a node score (the Average nodes score) that would be able to use both corrected and uncorrected p-values. As a matter of fact, this is the reason why we coined it "Average nodes score" and not "average z-score", so that the user can decide to use FDR or uncorrected p-values as inputs. For the benchmark, we wanted to test different settings. Sim_normal used uncorrected p-values and Samp_TCGA was using a sampling of DEG from FDR corrected p-values. In addition, MOGAMUN uses the significant DEGs as seeds to generate the initial population of individuals. In the current benchmark, using 483 potential seeds instead of 20, when using the raw p-values instead of the FDR-corrected p-values, respectively, allows building a more challenging setting for our tests. We added a sentence in the manuscript to state this point.

We however had tested MOGAMUN on the Sim_normal experiment using corrected p-values. MOGAMUN finds 6 modules, all the modules are composed of 15 nodes, and the mean Jaccard similarity between the modules is 0.46 on average. MOGAMUN retrieves 18/20 DEGs, as when

p-values are not corrected, but the F1 score is 0.64. This confirms that this was indeed an easier task.

10. Figure 2 has very poor quality.

We checked the figure used in the initial submission: we used a high-quality svg image, we don't understand why it finally appeared with low quality. We changed the format to png, hoping it will be better.

11. Some references for module discovery in multiplex networks:

Haiyan Hu, Xifeng Yan, Yu Huang, Jiawei Han and Xianghong Jasmine Zhou, Mining coherent dense subgraphs across massive biological networks for functional discovery, Bioinformatics 2005.

CODENSE, the algorithm developed by Hu et al. can be used to find dense subnetworks in any number of networks processed simultaneously. These networks could indeed be organized as a multiplex network. The main difference between MOGAMUN and CODENSE is that, besides finding dense subnetworks, MOGAMUN also considers the overall deregulation of the subnetworks, which helps contextualizing them.

Yu, L., Yao, S., Gao, L., & Zha, Y. (2019). Conserved Disease Modules Extracted From Multilayer Heterogeneous Disease and Gene Networks for Understanding Disease Mechanisms and Predicting Disease Treatments. Frontiers in genetics, 9, 745.

Yu et al. used the tensor-based computational framework proposed by Li et al. in 2011 to extract conserved disease modules from multilayer networks. Each layer represents disease-disease relationships, where the weight of the edges corresponds to the similarity between disease pairs. Their method is based on community detection, and each conserved disease module is a recurrent heavy subnetwork, whose nodes are consistently connected in the different layers.

Huang, X., Chen, D., Ren, T. et al. A survey of community detection methods in multilayer networks. Data Min Knowl Disc (2020). https://doi.org/10.1007/s10618-020-00716-6

This paper presents several community detection methods. As stated previously, community detection and active modules identification are two different tasks tackled by the biological network community. Huang et al. define that the purpose of community detection is to use the topology of the network(s) to find the most reasonable partitions (i.e., communities), where the nodes belonging to a community are expected to be more connected with themselves than to other nodes outside the community. In contrast, the identification of active modules is about 1) integrating networks and expression data (or any metric that can be used to set the weights of the nodes), and 2) finding subnetworks with deregulated nodes.

Huang et al. highlight the importance to reformulate and/or adapt the metrics that are normally defined to evaluate the subnetworks in monolayer networks. They grouped the different evaluation functions in 1) single score metrics, 2) evaluation using generated networks, and 3)

evaluation using real networks with known ground truth. Huang et al. remark that early approaches for community detection in multilayer networks were criticized for ignoring the connections across the layers, by either merging all the layers into a single weighted layer or identifying the communities in each layer independently and then making a consensus clustering to merge the results. On the other hand, direct methods analyze the multilayer network considering the inter and intra-layer connections.

De Domenico, M., Lancichinetti, A., Arenas, A. & Rosvall, M. Identifying modular flows on multilayer networks reveals highly overlapping organization in interconnected systems. Phys. Rev. X 5, 011027 (2015).

This paper is mainly an extension of [Rosvall and Bergstrom, 2008] to multilayer networks. The method aims at identifying modules in directed multilayer networks by compressing the flow network (network whose arcs are weighted, the sum of incoming flows having to be equal to that of outgoing flows). The approach is based on random walks. Intuitively communities are groups of connected nodes that capture a flow over a relatively long time (i.e. where the particle of the random walk spends a significant amount of time). The method uses Huffmann coding to rename the nodes. The length of the name associated with a node being inversely proportional to the time the walker spent on it (i.e. highly visited nodes have a short name, those visited rarely have a long name). It also seeks (above all) that the set of names reflect the structures of the network, i.e. the communities. The compression is therefore made at two different levels, while minimizing a map equation. The problem being NP-hard, the space of solutions is explored by a greedy heuristic, and then refined by a simulated annealing procedure. The numerical experiments performed by the authors show that the method can accurately find modules that can not be identified in an aggregated network, thus confirming that a multilayer approach better captures a network organization.
