# Supplementary material for: A multi-objective genetic algorithm to find active modules in multiplex biological networks
Source: PLoS Comput Biol. 2021 Aug 30;17(8):e1009263. doi: 10.1371/journal.pcbi.1009263 (PMC8452006; doi:10.1371/journal.pcbi.1009263)
Supplement: Attachment — Submitted filename: Response to Reviewers.pdf [file pcbi.1009263.s003.pdf]

Reviewer's Responses to Questions

Comments to the Authors:

Please note here if the review is uploaded as an attachment.

Reviewer #1: My major concerns with the work regarding comparison with other algorithms that have a similar goal and more robust testing of the proposed method, were addressed.

We thank the reviewer for their comments that enhanced the robustness of our analyses.

Reviewer #2: Minors:

Line 462: "the F_1" ==> "The F_1"

We corrected it, thanks.

In the caption of Figure 4: "2014 cite34," Is it correct?

It was indeed an error. Thank you for noticing it.

Check the last author's name in ref.[22], i.e. ",T..A.M.T. " ??

We corrected it, thanks. We thoroughly checked all the references and also corrected ref. [26].

Reviewer #3: I appreciate the authors' effort in addressing my comments which have significantly improved the clarity of the manuscript. However, I feel a number of major issues have not been resolved.

We thank the reviewer for their remarks that enhanced the robustness of the evidence we show in our manuscript.

Three major issues:

1. The evaluation of the proposed method lacks rigor and is not a fair comparison, as stated in the original comments. The proposed method, MOGAMUN, relies on a critical parameter, min_module_size. In fact, most modules identified by MOGAMUN has size very close to or slightly above min_module_size. For simulated data, this is a huge advantage for the proposed method as the authors can choose a min_module_size (15) close to the actual size (20). In contrast, the module size by the three competing methods (avg = 6, 640, and 6952, respectively) are VERY different from the actual size. I understand that the authors used default parameters from the competing methods; however, I believe the authors need to put some extra effort here to tune the parameters to make sure the module sizes are at least somewhat closer

to the actual size for the simulated data. The size difference may be the most important reason that the competing methods had a much lower F-1 score and density (Fig 2, 3).

We disagree that our protocol lacks rigor, and we intended to provide as fair as possible comparisons with the other methods. In the benchmark, the choice of the number of foreground genes was guided by the protocol we used as a reference, which defines 20 foreground genes (Batra et al. 2017). We in addition used the following parameters:

- PinnacleZ has 3 parameters: the maximal distance from the seed, the maximum module size, and the minimal mutual information score improvement. We used default parameters, except for the maximum module size that we set to 50, to match the maximum module size selected for MOGAMUN.
- COSINE has 3 parameters: lambda, the zero to one ratio, and the number of iterations. We used the default parameters, except for the lambda parameter that we set to 0.5 to give the same importance to the retrieval of high-scoring nodes and edges, which could be considered as equivalent to the optimization of the two objective functions we defined in MOGAMUN.
- jActiveModules has 5 general parameters: the number of modules to be retrieved, the overlap threshold (useful when the number of modules > 1), the number of iterations, and the start and final temperature values. We used the default parameters except that we set the number of active modules to be retrieved to 1 in the benchmark, because the benchmark task was to retrieve a single active module. This is a clear advantage for jActiveModules, as it is the only method for which the number of modules to be retrieved can be defined *a priori*. For the real-case application of jActiveModules, as the real number of active modules to be retrieved is unknown, we used the default parameter of 5 modules to be retrieved.

As shown in our manuscript and noticed by the reviewer, jActiveModules and COSINE tend to find very large active modules. In fact, it has been shown that the aggregated node score used in some active module identification algorithms (such as jActiveModules) is biased toward the identification of very large subnetworks (Nikolayeva et al. 2018). Importantly, MOGAMUN is the only method that has both minimum and maximum module sizes parameters. Setting such module size parameters in jActiveModules, PinnacleZ and COSINE is not trivial. Indeed, it would imply to modify the code and even deeply change the algorithms. For instance, for the greedy search used in PinnacleZ, one node is added in each iteration as long as it improves the score of the module. But if a minimum size were to be defined, it might imply including nodes with non-optimal scores, and it would therefore no longer be a greedy algorithm. Similarly, adding a parameter to control the module sizes in jActiveModules and COSINE would also imply to modify their code to, for instance, keep iterating until reaching the desired size.

We however tested if we can modify the sizes of the modules obtained by COSINE, PinnacleZ and jActiveModules using the available parameters. We considered that by changing the number of iterations of COSINE and jActiveModules, both algorithms would have more time to adjust the sizes of the subnetworks they retrieve to potentially reduce their sizes. Similarly, if the distance from the seed is bigger, PinnacleZ would be able to explore further the network and

potentially increase the size of the modules it retrieves. We thus increased the maximal distance from the seed from 2 (default value) to 5 in PinnacleZ and found that the modules are indeed bigger, but most of them have sizes of 4-6 nodes, and only one out of the ~3,000 obtained subnetworks had 14 nodes. We also increased the number of iterations (2x, 3x and 4x) in jActiveModules and COSINE. We found that the subnetworks are smaller than with the default number of iterations, but they are still very large (>1000 nodes for jActiveModules and >100 nodesfor COSINE). The longest running time of COSINE was ~25 hours (20,000 iterations = 4x the default value of 5,000). Surprisingly, the running time of jActiveModules was constant even for one million iterations, but the results were always very large modules. In summary, tuning the available parameters did not drastically change the results obtained: the subnetworks retrieved by COSINE and jActiveModules are still very large, and those retrieved by PinnacleZ are still small.

In order to emphasize this point, we added the following paragraph to the discussion:

"In the benchmark analyses, we observed that jActiveModules and COSINE tend to retrieve very large subnetworks (up to hundreds or even thousands of nodes), whereas PinnacleZ tends to retrieve very small ones (mostly composed of 2 or 3 nodes). Although jActiveModules, COSINE and PinnacleZ were able to retrieve most or all of the simulated foreground genes, their F_1 scores were lower than those of MOGAMUN because they selected more background genes (i.e., false positives). Importantly, only PinnacleZ and MOGAMUN have a user-defined threshold to limit the maximum size of the modules, and MOGAMUN is the only method having a user-defined threshold to limit the minimum size of the modules. Importantly, only PinnacleZ and MOGAMUN have a user-defined threshold to limit the maximum size of the modules, and MOGAMUN is the only method having a user-defined threshold to limit the minimum size of the modules."

REFERENCES

Barabási, A. L., Gulbahce, N., & Loscalzo, J. (2011). Network medicine: a network-based approach to human disease. *Nature reviews genetics*, *12*(1), 56-68.

Batra R, Alcaraz N, Gitzhofer K, Pauling J, Ditzel HJ, Hellmuth M, et al. On the performance of de novo pathway enrichment. NPJ Syst Biol Appl. 2017 Mar 3.

Nikolayeva,I., Guitart-Pla,O. and Schwikowski,B. (2018) Network module identification—A widespread theoretical bias and best practices. Methods, 132

2. I am also concerned with the fact that most modules have size very similar to the predefined min_module_size, which means a relatively larger module will be reported as MANY smaller modules with significant overlaps. The reported modules in the supplementary file indeed show that. For example, in supplementary Fig 13, the 17 active modules seem to be subnetworks of two larger modules.

In MOGAMUN, our choice of the default parameters for module sizes, in the 15-50 range, was selected after discussion with biologists as allowing the easiest visualisation and biological interpretations. We know that the active modules found by MOGAMUN tend to have the minimum size allowed by the parameter. This bias arises from the sparsity and small-world property of biological networks (Barabási et al., 2011). This point is mentioned explicitly in the parameter values (now section 2.3 of the Materials and Methods): "The minimum size parameter is important as real biological networks are sparse and the density definition will tend to favor smaller subnetworks. The final subnetworks will tend to have sizes equal to the minimum size allowed.".

Biology is multiscale and smaller biological processes (i.e., potentially small active modules) are encompassed in bigger biological processes (i.e., bigger active modules). Moreover, if a gene is present in various modules, it might be because it is an important gene and/or because it has various functions (Chapple et al., 2015). We consider that small modules allow a better visualisation of the genes and their interactions, which eases biological interpretation, but we are aware that needs of a user can vary. In order to give more flexibility to the users, we implemented a post-processing step to merge overlapping active modules according to a user-defined overlapping threshold. This post-processing step was already part of the MOGAMUN Bioconductor package (*mogamun_postprocess* function).

We added a section 2.2 in the Materials and methods to describe the post-processing of the modules using the set of modules obtained in the accumulated Pareto front. In this post-processing step, small overlapping modules can be merged into bigger modules if their Jaccard similarity coefficient is above a given threshold. The higher the threshold, the bigger the overlap needed in order to merge two subnetworks. It is to note that, if the threshold is low and due to the small-world property of biological networks, the user might obtain a unique large module. The new section 2.2 states:

" 2.2 Post-processing the results
We designed a post-processing step to remove redundancy in the individuals obtained in the *accumulated Pareto front*. We calculated the Jaccard similarity coefficient (equation 5) between every pair of individuals and merged them if it is higher than a given threshold J_{t2}."

We also added a paragraph to the discussion to mention the importance of the minimal module size parameter:

"However, MOGAMUN tends to find subnetworks of the minimum size allowed (or very close to it). This is expected, given the sparse nature of biological networks. This bias towards minimum size active modules could lead to having a large big active module cut into several smaller subnetworks, which are nonetheless easier to visualize and interpret. Overall, the *MinS* parameter has to be chosen carefully, depending on the user's needs. We implemented in addition a post-processing step to merge overlapping subnetworks, according to a user-defined threshold. It is to note that, given the small-world property of biological networks, if this threshold is too low, the result might be a single (large) active module. We therefore recommend trying different thresholds values."

REFERENCES

Barabási, A. L., Gulbahce, N., & Loscalzo, J. (2011). Network medicine: a network-based approach to human disease. *Nature reviews genetics*, *12*(1), 56-68.

Chapple, C. E., Robisson, B., Spinelli, L., Guien, C., Becker, E., & Brun, C. (2015). Extreme multifunctional proteins identified from a human protein interaction network. *Nature communications*, *6*(1), 1-9.

3. The authors claim that their method is the first to deal with multiplex networks. It may be true that no other method has utilized multiple networks the same way as the authors did, but the authors, in my opinion, failed to demonstrate the benefit of their approach of combining multiple networks. First, the simulated data has only a single network (which the authors has given a reason, so not the point to discuss here.). Then, on the real-world data, the authors compared two different ways of using multiple networks: (1) their original MAGAMUN, which is equivalent to precomputing a weighted merge of the networks and then trying to optimize subnetwork density on the weighted network, and (2) compute an UNWEIGTHED merge of the networks, which is then used by the competing algorithms and MAGAMUN to identify active modules. The results are presented as Sec 5 of the supplementary file and very briefly mentioned in manuscript. Similar to point 1 above, these results mostly shows that the module size from the other algorithms are either "too small" or "too large", which again, potentially could be addressed by the competing methods fairly easily. There is also not sufficient comparison of the two versions of MAGAMUN (with weighted or unweighted aggregated networks).

As mentioned in the response to the first specific comment of the reviewer from the previous revision, multiplex and multilayer frameworks have been demonstrated to provide overall more complex but richer information frameworks (Kivelä et al. 2014), in various contexts (De Domenico et al. 2014, De Domenico et al. 2016, Halu et al. 2019). For instance, we showed the pertinence of the use of multiplex approaches to identify communities (Didier et al. 2015, Didier et al. 2018) or rank nodes using random walk with restart (Valdeolivas et al. 2019). In the context of MOGAMUN, with the goal of finding active modules, we did not identify any metrics allowing us to compare the active modules obtained using a multiplex versus an aggregated network. Indeed, the objective functions cannot be used for this comparison. The multiplex or aggregated densities will by definition favor the modules obtained from the multiplex or aggregated networks, respectively. Moreover, since the density of edges is higher in the aggregated network as compared to the density of the individual layers of the multiplex, high-scoring nodes have higher chances to be connected. As a result, both the density (obtained with the classical density measure) and the average nodes score of the results of MOGAMUN can be higher in the aggregated network than in the multiplex (Supplementary Section 5). However, using the aggregated networks, the obtained modules will mainly reflect the interactions of the largest network layer. In our case, this corresponds to the co-expression network, which is much larger but also more noisy. The observed interactions between high-scoring nodes and the obtained active modules will hence be dominated by prone-to-noise

interactions, and more difficult to interpret from a biological point of view. To better evaluate the differences between the active modules obtained on the multiplex versus aggregated networks, it would be interesting to implement a benchmark based on biological proxies, using for instance Gene Ontology enrichments to evaluate the pertinence of the obtained active modules. Such a strategy has been developed recently in the DOMINO paper (Levi et al. 2021). It is, however, beyond the scope of the current manuscript.

We added the following paragraphs to the discussion to clarify this:

“In addition to the benchmark with artificial expression data, we also applied the four methods to the three RNA-Seq datasets of FSHD1 from [34]. MOGAMUN is the only method that can take as input more than one network. Hence, in order to apply all the approaches, we aggregated the three network layers of the multiplex biological network (Table 4). The behaviour of jActiveModules, COSINE and PinnacleZ is similar in this real-case experiment as in the benchmark analyses (see supplementary section 5 for detailed results). jActiveModules and COSINE tend to retrieve large subnetworks, and PinnacleZ finds only very small ones (Supplementary Figures S17-S19). We further observed that, as expected, the simple density (equation 4) is higher for MOGAMUN's active modules obtained from the aggregated networks, whereas the normalized density (equation 3) is higher for MOGAMUN's active modules obtained from the multiplex network (Supplementary Figures S17-S19). In addition, since the aggregated network is denser than any of the individual network layers, the chances of connecting high-scoring nodes is also higher. In the future, a benchmark based on biological proxies, using for instance Gene Ontology annotation enrichments [48], as recently proposed in the DOMINO approach [49], could be used to compare the active modules obtained from aggregated versus multiplex networks.”

Regarding changing the sizes of the obtained modules by jActiveModules, PinnacleZ and COSINE and as mentioned in our response to the first comment of the reviewer, our efforts to tune the available parameters of the three methods were in vain. Modifying the core code of the competing methods, introducing new parameters and providing sensitivity analyses for these methods is beyond the scope of this manuscript. It is to note that none of the four methods used in our manuscript (jActiveModules, PinnacleZ, COSINE and MOGAMUN) can handle weighted networks. We added the following sentences to discussion to clarify this:

“Both the weights of the nodes and edges in COSINE are calculated from the expression data (section 2.4.3), and they cannot be manipulated by the user. Importantly, none of the four methods compared in this manuscript can handle weighted networks.”

References

De Domenico, M., Solé-Ribalta, A., Gómez, S., & Arenas, A. (2014). Navigability of interconnected networks under random failures. *Proceedings of the National Academy of Sciences*, *111*(23), 8351-8356.

De Domenico, M., Granell, C., Porter, M. A., & Arenas, A. (2016). The physics of spreading processes in multilayer networks. *Nature Physics*, *12*(10), 901-906.

Didier, G., Brun, C., & Baudot, A. (2015). Identifying communities from multiplex biological networks. *PeerJ*, *3*, e1525.

Didier, G., Valdeolivas, A., & Baudot, A. (2018). Identifying communities from multiplex biological networks by randomized optimization of modularity. *F1000Research*, *7*.

Halu, A., De Domenico, M., Arenas, A., & Sharma, A. (2019). The multiplex network of human diseases. *NPJ systems biology and applications*, *5*(1), 1-12.

Kivelä, M., Arenas, A., Barthelemy, M., Gleeson, J. P., Moreno, Y., & Porter, M. A. (2014). Multilayer networks. *Journal of complex networks*, *2*(3), 203-271.

Levi, H., Elkon, R., & Shamir, R. (2021). DOMINO: a network-based active module identification algorithm with reduced rate of false calls. *Molecular Systems Biology*, *17*(1), e9593.

Valdeolivas, A., Tichit, L., Navarro, C., Perrin, S., Odelin, G., Levy, N., ... & Baudot, A. (2019). Random walk with restart on multiplex and heterogeneous biological networks. *Bioinformatics*, *35*(3), 497-505.

Two relatively minor issues:

4. Fig S16 does NOT show that the algorithm is insensitive to parameter tuning. A boxplot only shows that the distribution of node scores and densities are somewhat similar across different network parameters. It does not show that the actual network results are similar.

We agree with the remark of the reviewer about Figure S16. To complement the results obtained previously, we made an UpSet plot to show the overlap between the active modules obtained using different sets of parameters. We observed that the overlap of all the combinations of crossover and mutation rates is indeed large, proving that MOGAMUN retrieves similar results for high crossover rates and low mutation rates. This UpSet plot is now Figure S16 C, and we extended the explanation in the supplementary material to mention this new Figure and results.

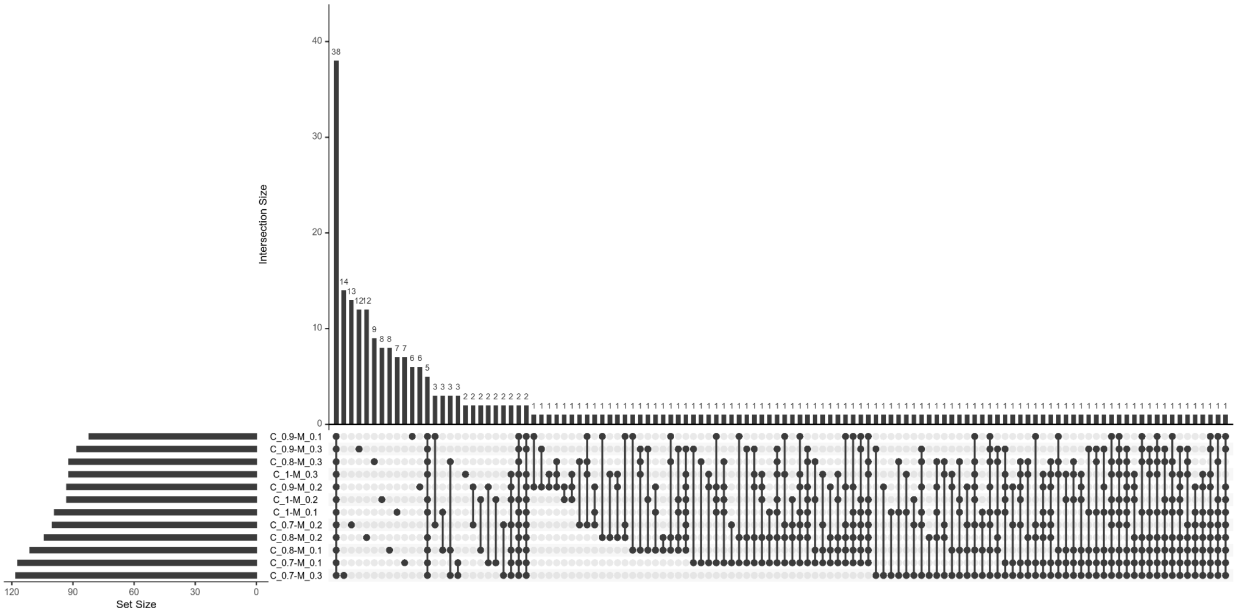

5. The authors have included a lot of suppl figs and tables, but many of them are not referred / discussed in the manuscript.

The full set of new figures and tables are part of the supplementary section 5. We indeed do not discuss each figure and table individually in the manuscript, but refer twice to the full supplementary section indicating that the readers can find there the detailed analyses of the application of all the methods to the FSHD1 datasets.
